# Antimicrobial Activity of 1,3,4-Thiadiazole Derivatives

**DOI:** 10.3390/ph18091348

**Published:** 2025-09-08

**Authors:** Sebastian Górecki, Agnieszka Kudelko, Monika Olesiejuk

**Affiliations:** Department of Chemical Organic Technology and Petrochemistry, The Silesian University of Technology, Krzywoustego 4, PL-44100 Gliwice, Poland; sg301135@student.polsl.pl (S.G.); agnieszka.kudelko@polsl.pl (A.K.)

**Keywords:** 1,3,4-thiadiazoles, biological activity, antibacterial, antifungal, medicine, agriculture

## Abstract

The 1,3,4-thiadiazole core has attracted significant attention due to its unique electronic structure, physicochemical properties, and wide-ranging pharmacological potential. This heterocyclic scaffold exhibits a broad spectrum of biological activities, often attributed to its capacity to modulate enzyme function, interact with receptors, and disrupt key biochemical pathways in both pathogens and host cells. Additionally, 1,3,4-thiadiazoles typically display favorable pharmacokinetic properties, including high metabolic stability and appropriate lipophilicity, which enhance their drug-likeness and bioavailability. This review presents an overview of antibacterial and antifungal compounds bearing the 1,3,4-thiadiazole scaffold that have been reported over the past five years. This publication details the chemical structures of novel 1,3,4-thiadiazole derivatives and reports the results of antibacterial and antifungal activity assays conducted against a range of microbial strains. Furthermore, it provides conclusions regarding the structural features that influence the observed biological activity of the synthesized compounds. Antimicrobial activity assessments conducted against ten Gram-negative and nine Gram-positive bacterial strains revealed that 79 newly synthesized 1,3,4-thiadiazole derivatives exhibited either superior inhibitory efficacy relative to standard reference antibiotics or achieved a high level of bacterial growth suppression, defined as 90–100% inhibition. In antifungal assays, the compounds were evaluated against 25 fungal species representing 15 genera. Among the tested derivatives, 75 compounds demonstrated antifungal potency exceeding that of reference antifungal agents or produced growth inhibition within the 90–100% range. The information provided herein may serve as a valuable resource for medicinal and agricultural chemists engaged in the development of novel drug candidates and plant protection agents.

## 1. Introduction

**Figure 1:**
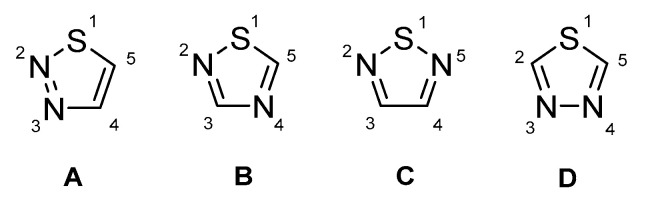
Possible isomers of thiadiazole core: **A**: 1,2,3-thiadiazole; **B**: 1,2,4-thiadiazole; **C**: 1,2,5-thiadiazole; **D**: 1,3,4-thiadiazole.

An important class of five-membered heterocyclic compounds is represented by thiadiazoles, which contain one sulfur atom and two nitrogen atoms in their structure [1,2,3]. This system gives rise to four possible isomers (Figure 1), among which the 1,3,4-thiadiazole isomer is the most widely studied and utilized due to its extensive applications in industry [4,5,6,7,8,9,10,11], medicine [12,13,14,15,16,17], and agriculture [18,19,20,21,22,23,24].

**Scheme 1:**
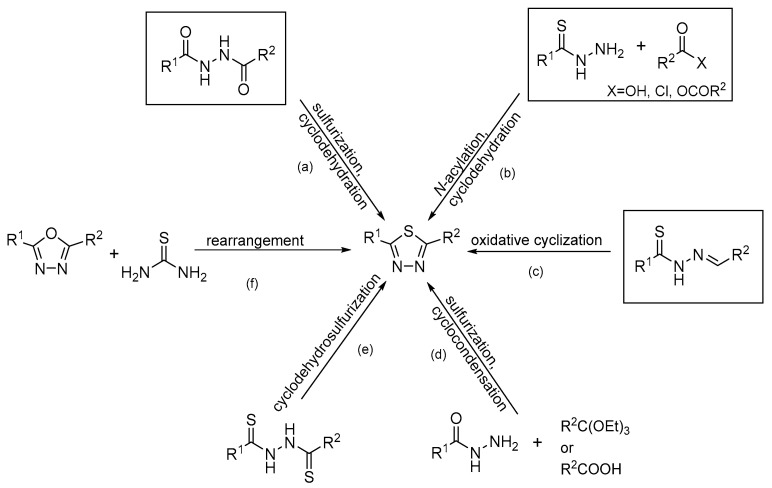
The most popular synthetic routes for the disubstituted 1,3,4-thiadiazole derivative: (**a**): sulfurization and cyclodehydration step; (**b**): *N*-acylation and cyclodehydration step; (**c**): oxidative cyclization step; (**d**): sulfurization and cyclocondensation step; (**e**): cyclodehydrosulfurization step; (**f**): rearrangement step.

The chemistry of heterocyclic compounds employs various methods for the construction of the 1,3,4-thiadiazole ring [1,2,14]. The most widely used approach is undoubtedly cyclodehydration, typically preceded by a sulfurization step, of diacylhydrazine derivatives (Scheme 1a) or monothiodiacylhydrazines, which are most often formed as intermediates in the reaction of thiohydrazides with carboxylic acid derivatives (Scheme 1b). Another frequently applied method for the synthesis of 2,5-disubstituted 1,3,4-thiadiazoles involves the oxidative cyclization of thioacylhydrazones (Scheme 1c). Additional strategies include the sulfurization of hydrazides followed by cyclocondensation, most commonly with ortho-esters (Scheme 1d), cyclodehydrosulfurization of dithioacylhydrazine derivatives (Scheme 1e), or ring rearrangement reactions, most often involving 1,3,4-oxadiazoles (Scheme 1f). All sulfurization-based methods are typically carried out in the presence of P_4_S_10_ or Lawesson’s reagent.

**Figure 2:**
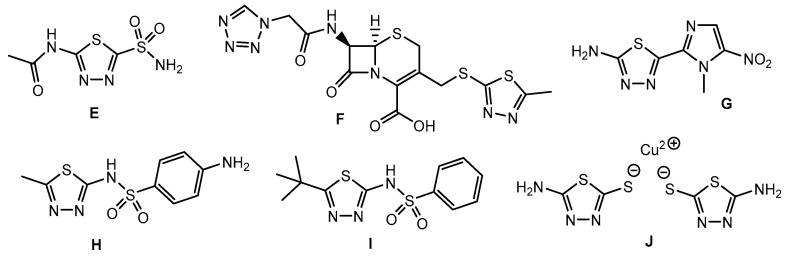
Representative drugs and reference standards characterized by the presence of the 1,3,4-thiadiazole ring. **E**: Acetazolamide—diuretic, antiglaucoma, anticonvulsant, and treatment for altitude sickness. **F**: Cefazolin—treats bacterial infections (especially Gram-positive). **G**: Megazol—antiparasitic agent (investigated for Chagas disease and sleeping sickness). **H**: Sulfamethizole—treats urinary tract infections (UTIs) and other bacterial infections. **I**: Gludiase—treats type 2 diabetes mellitus by stimulating insulin secretion from pancreatic beta cells. **J**: Thiodiazole copper—a type of copper-based antimicrobial compound often used to control a wide range of plant diseases caused by bacteria and fungi.

The emergence of heterocyclic compounds as key pharmacophores in modern medicinal chemistry has fueled extensive research into novel scaffolds with potent and selective biological activities. The favorable pharmacokinetic properties of 1,3,4-thiadiazole derivatives are attributed to their unique chemical structure and mesoionic nature. These compounds exhibit enhanced lipophilicity and membrane permeability, facilitating effective interactions with biological targets [25,26]. The ring structure offers a balance between hydrophilicity and lipophilicity, which supports membrane permeability and bioavailability. Furthermore, studies on drug–1,3,4-thiadiazole conjugates have assessed their compliance with Lipinski’s Rule of Five, a set of guidelines predicting good oral bioavailability. These compounds demonstrated appropriate molecular weight, hydrogen bond donor and acceptor counts, and lipophilicity (log P values), all of which suggest favorable pharmacokinetic properties [26]. In addition, the electron-rich nature of thiadiazoles and their ability to form hydrogen bonds or coordinate with metal ions allow them to function as enzyme inhibitors or receptor ligands [27,28,29]. These attributes make 1,3,4-thiadiazole derivatives promising candidates for drug development, offering an optimal balance between efficacy and safety (Figure 2).

When evaluating antimicrobial activity, several standard methods and parameters are commonly used across microbiology, pharmacology, and materials science. These tests help determine the effectiveness of a compound, extract, or material against specific bacterial or fungal strains. Testing antifungal activity shares many similarities with antibacterial testing; however, fungi—especially yeasts and filamentous species—exhibit different growth characteristics, and methods are therefore adapted accordingly. The most commonly used parameters for assessing antimicrobial efficacy include the following:

- MIC (minimum inhibitory concentration): The lowest concentration that prevents visible bacterial or fungal growth.
- MBC (minimum bactericidal concentration): The lowest concentration that kills 99.9% of bacterial cells.
- MFC (minimum fungicidal concentration): The lowest concentration that kills 99.9% of fungal cells (confirmed by subculturing).
- Zone of inhibition: The diameter (in mm) of the clear, growth-free area around a disk or well.
- Inhibition rate: Typically used in colorimetric assays; represents the reduction in microbial growth relative to an untreated control (%).

**Figure 3:**
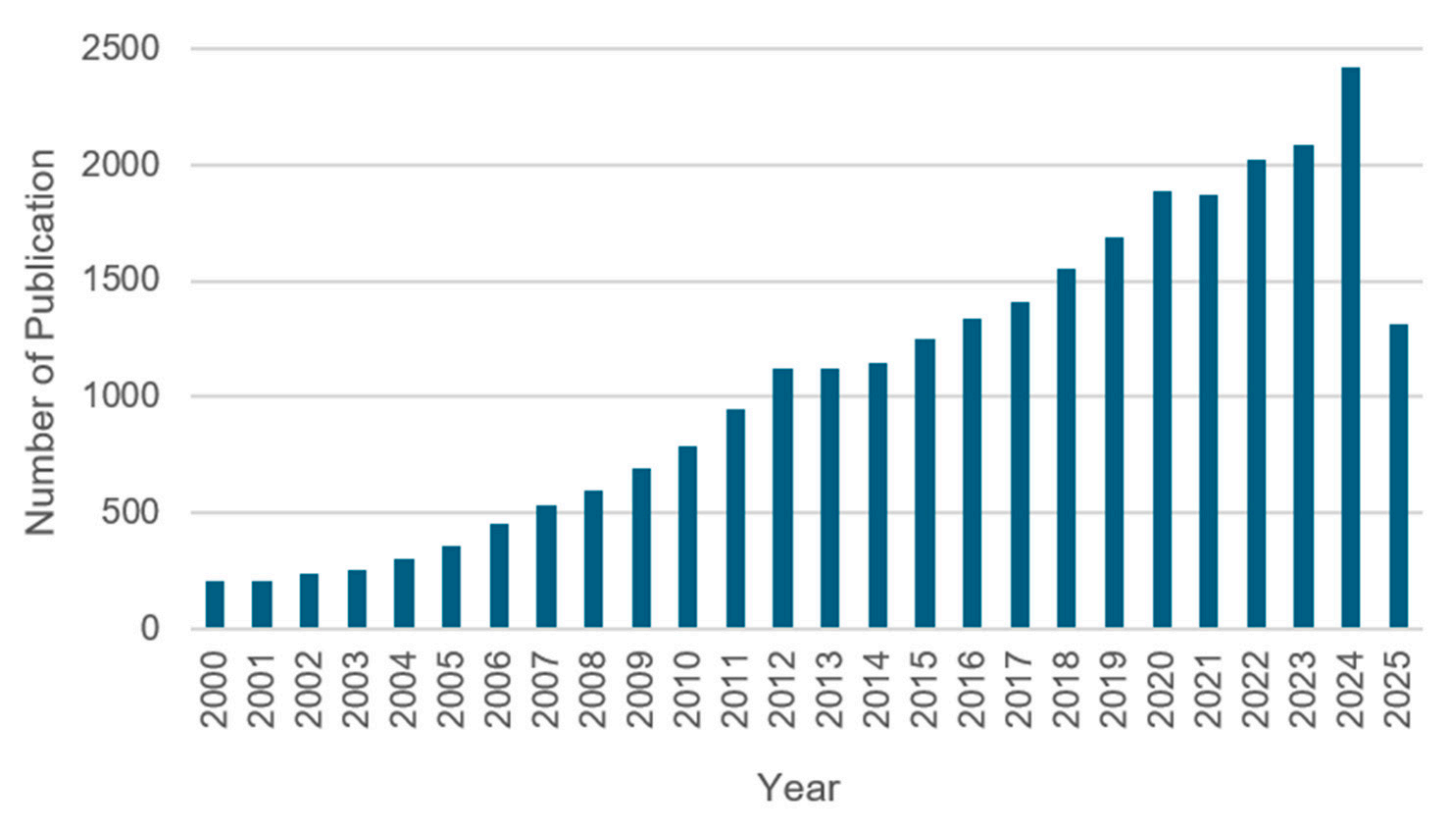
Number of relevant articles related to 1,3,4-thiadiazole published between 2000 and 2025. The data were obtained from Scopus. Search: 1,3,4-thiadiazole; all fields (accessed June 2025).

Over the years, there has been a notable surge in the synthesis and pharmacological evaluation of 1,3,4-thiadiazole derivatives (Figure 3). This growing interest is driven by their demonstrated activity across a wide range of biological domains, including antibacterial, antifungal, antiviral, anticancer, anti-inflammatory, and central nervous system (CNS) disorders [30,31,32,33,34,35]. Recent studies have also highlighted their potential as enzyme inhibitors, receptor modulators, and multi-target agents, often exhibiting favorable pharmacokinetic and safety profiles [36,37,38,39,40,41,42].

The objective of this review is to provide a systematic analysis of studies published between 2020 and 2025, highlighting the most notable advancements in the biological evaluation of 1,3,4-thiadiazole-based compounds. Particular attention is given to compounds exhibiting antibacterial and antifungal properties for antimicrobial applications. By highlighting the most recent advances, this article seeks to provide medicinal chemists and pharmaceutical scientists with an up-to-date and focused perspective on the potential of the 1,3,4-thiadiazole scaffold in drug discovery and plant protection products.

## 2. 1,3,4-Thiadiazole Derivatives with Biological Activity

### 2.1. Antibacterial Activity

#### 2.1.1. Disubstituted 1,3,4-Thiadiazole Derivatives

Among the published articles, the most frequently studied derivatives are 1,3,4-thiadiazole derivatives substituted at positions 2 and 5 of the heterocyclic ring.

**Figure 4:**
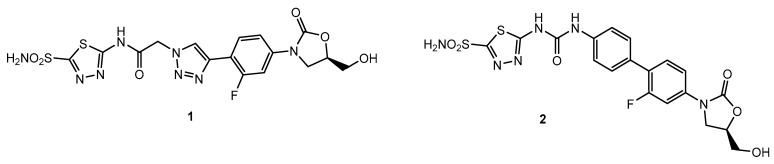
Oxazolidinones containing a 1,3,4-thiadiazole ring.

Ammara et al. synthesized oxazolidinone derivatives, two of which contained a 1,3,4-thiadiazole ring (**1**, **2**, Figure 4) [43]. The obtained compounds incorporated an oxazolidinone core in the (*S*)-configuration at the C-5 position, which, according to the authors, is essential for antibacterial activity, as well as a fluorine atom substituted at the phenylene linker, a modification that typically enhances potency by 2–8 fold. Compound **1** exhibited low activity against *Enterococcus faecalis* (31971), *Enterococcus faecalis* (31972), and *Enterococcus faecium*, with MIC values of 64 μg/mL, 32 μg/mL, and 32 μg/mL, respectively. In contrast, compound **2** showed moderate activity against *Enterococcus faecalis* (31971), *Enterococcus faecalis* (31972), and *Enterococcus faecium*, with MIC values of 2 μg/mL, 2 μg/mL, and 1 μg/mL, respectively. It showed no activity against the oxazolidinone-resistant strain *Enterococcus faecalis* (31903), with MIC > 64 μg/mL.

**Figure 5:**
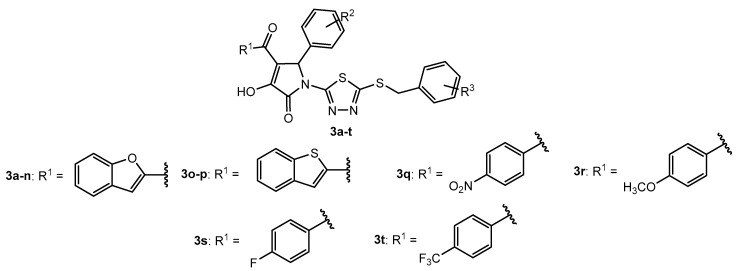
Dihydropyrrolidone derivatives with 1,3,4-thiadiazole core.

Xiong et al. synthesized a series of dihydropyrrolidone derivatives containing the 1,3,4-thiadiazole moiety (**3a**–**t**, Figure 5) and evaluated their activity against *Staphylococcus epidermidis*, *Staphylococcus aureus*, *Enterococcus faecalis*, and *Enterococcus faecium* [44]. The compounds and their corresponding MIC values are presented in Table 1. Derivatives **3c**, **3i**, and **3j**, characterized by the presence of a hydroxyl group at the *ortho* position of the benzene ring and a chlorine atom at the R^3^ site, as well as **3n**, containing two hydroxyl groups at the R^2^ site, exhibited the highest activity against Gram-positive bacteria. Moreover, all compounds demonstrated low cytotoxicity and hemolytic activity.

**Figure 6:**
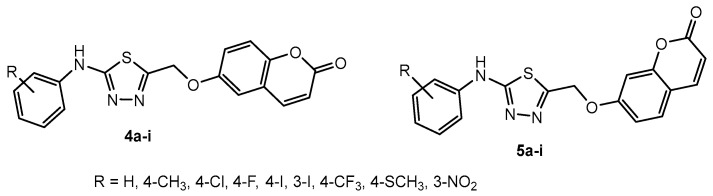
Two series of 1,3,4-thiadiazole derivatives containing (2*H)*-chromen-2-one moiety.

Gumus et al. synthesized a series of 1,3,4-thiadiazole derivatives from thiosemicarbazide-substituted coumarins, yielding compounds **4a**–**i** and **5a**–**i** (Figure 6) [45]. Selected compounds were screened for antibacterial activity against *Helicobacter pylori*. The tested compounds did not exhibit antibacterial activity (MIC > 128 μg/mL).

**Figure 7:**
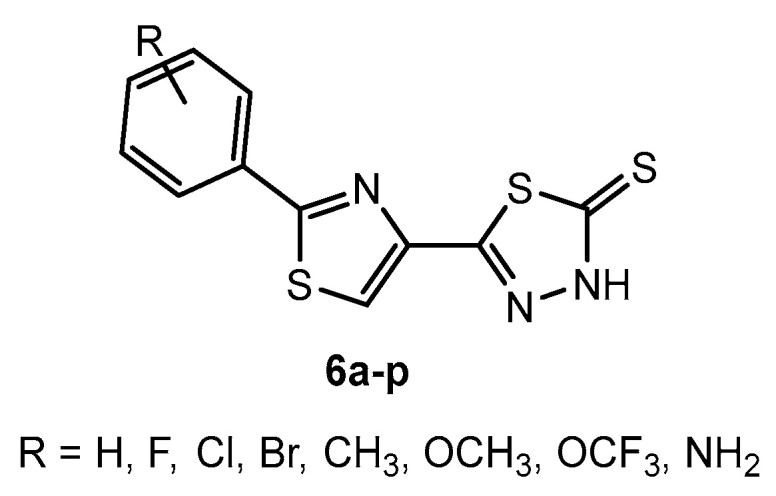
Phenylthiazole derivatives containing a 1,3,4-thiadiazole thione moiety.

Mao et al. obtained phenylthiazole derivatives containing a 1,3,4-thiadiazole thione moiety (**6a**–**p**, Figure 7) [46]. The synthesized compounds were tested for antibacterial activity against *Ralstonia solanacearum* and *Xanthomonas oryzae pv. oryzae*. The activity was evaluated at concentrations of 100 μg/mL and 200 μg/mL, with the data presented in Table 2. The highest activity against *Ralstonia solanacearum* was observed for compounds **6b** (R = 2-F), **6h** (R = 3-F), **6i** (R = 3-CH_3_), and **6k** (R = 3-OCF_3_), which showed inhibition rates of 92.00%, 93.81%, 94.00%, and 100%, respectively, at a concentration of 100 μg/mL. Moreover, compound **6k** exhibited a high inhibition rate of 72.63% against *Xanthomonas oryzae pv. oryzae* at the same concentration. The authors concluded that the antibacterial activity of the investigated phenylthiazole derivatives is strongly enhanced by electron-withdrawing substituents at the *meta*-position of the benzene ring, whereas for the *ortho*- and *para*-analogues the inhibition rate is much lower.

**Figure 8:**
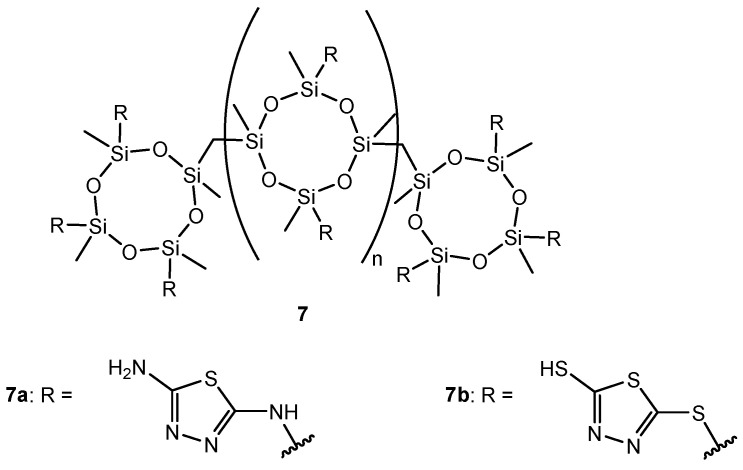
The structure of mesostructured nanohybrids of thiadiazole.

Hafidh et al. synthesized hybrid silica gels incorporating 1,3,4-thiadiazole rings in their structure (**7a**, **7b**, Figure 8) [47]. The compounds were evaluated for antibacterial activity against *Escherichia coli*, *Salmonella typhimurium*, *Staphylococcus aureus*, and *Enterococcus faecium*. The results of the biological screening are presented in Table 3.

**Figure 9:**
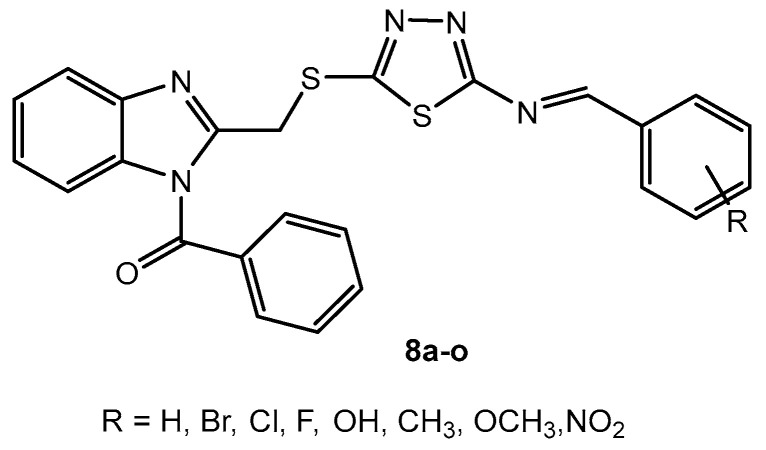
The structure of 1,3,4-thiadiazole with benzo[*d*]imidazole scaffolds.

Mehta’s group synthesized a series of 1,3,4-thiadiazole derivatives containing a benzo[*d*]imidazole scaffold (**8a**–**o**, Figure 9) [48]. The resulting compounds were evaluated for antibacterial activity against *Escherichia coli*, *Pseudomonas aeruginosa*, *Staphylococcus aureus*, and *Streptococcus pyogenes*, with the results presented in Table 4. The highest MIC values were observed for compounds **8j** (R = 4-OH) and unsubstituted **8a** (R = H) against *Pseudomonas aeruginosa*, both with values of 12.5 μg/mL. In contrast, compound **8e** (R = 4-Cl) exhibited the lowest MIC value, also 12.5 μg/mL, against *Staphylococcus aureus*. The studies demonstrated that electron-donating substituents, such as hydroxyl and methoxy groups at the *ortho* and *para* positions of the benzylidene fragment, enhanced the biological activity against *Escherichia coli*, *Pseudomonas aeruginosa*, and *Staphylococcus aureus*. Electron-withdrawing groups, such as bromo, chloro, and fluoro at the *para* and *ortho* positions, also showed significant activity against the same bacterial strains.

**Figure 10:**
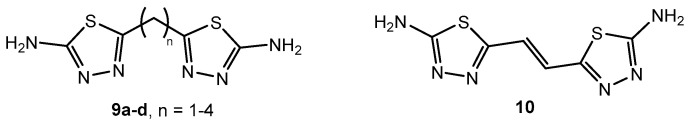
The structure of bisaminothiadiazoles.

The group of Danilova evaluated a series of bisamino-1,3,4-thiadiazoles linked via alkyl and alkenyl spacers (**9a**–**d**, **10**, Figure 10) against *Staphylococcus aureus*, *Citrobacter amalonaticus*, and *Escherichia coli* [49]. Among the tested compounds, **9a** (n = 1) exhibited activity against *Staphylococcus aureus* at a concentration of 3.36 × 10^−4^ M, with a zone of inhibition measuring 1.6 mm. In the case of *Citrobacter amalonaticus*, only compound **10** was active at a concentration of 3.01 × 10^−4^ M, also with a zone of inhibition of 1.6 mm. None of the tested compounds showed activity against *Escherichia coli*.

**Figure 11:**
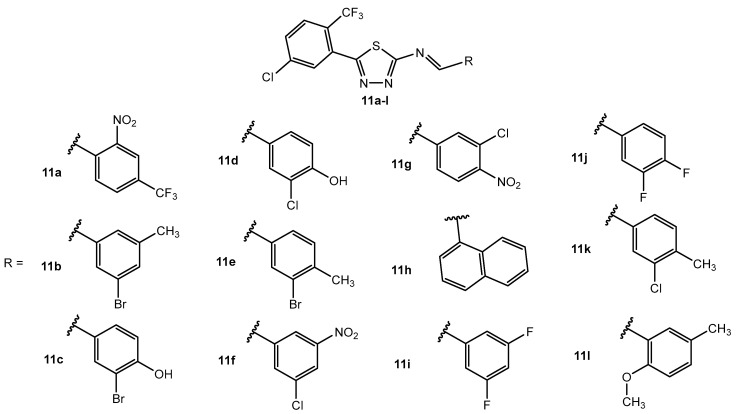
The structure of thiadiazoles bearing Schiff base moiety.

Zahoor et al. obtained Schiff base derivatives containing a 1,3,4-thiadiazole moiety (**11a**–**l**, Figure 11) [50]. The compounds were tested for antibacterial activity against *Escherichia coli*. The results are presented in Table 5. Four of the investigated compounds—**11a**, **11c**, **11d**, and **11i**—exhibited bacterial inhibition rates of 42.3%, 40.1%, 38.2%, and 36.5%, respectively, in comparison to Streptomycin (44%). The authors concluded that the presence of electron-withdrawing substituents (CF_3_, NO_2_, Cl, F) within the benzylidene fragment at favorable positions—mainly *para* and, less frequently, *ortho*—ensures strong inhibition. In contrast, weaker inhibition was observed for derivatives bearing bulky groups (CH_3_, Br, naphthyl), particularly at the *meta*-positions, due to steric hindrance.

**Figure 12:**
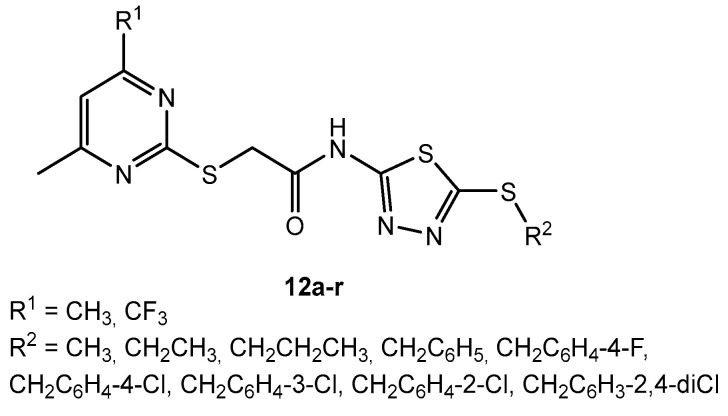
The structure of pyrimidine derivatives incorporating amide and 1,3,4-thiadiazole thioether moiety.

Li et al. obtained a series of 2,5-disubstituted 1,3,4-thiadiazole compounds (**12a**–**r**, Figure 12) [51]. The compounds were tested for antibacterial activity against *Xanthomonas oryzae pv. oryzae*. The results of the screening, performed at a concentration of 100 μg/mL, are presented in Table 6. Bioassay results demonstrated that all tested compounds exhibited superior antibacterial activity against *Xanthomonas oryzae pv. oryzae*, with inhibition rates ranging from 52% to 79%, compared to the Thiodiazole copper standard (16%) (**J**, Figure 2). Enhanced in vitro antibacterial activity against *Xanthomonas oryzae pv. oryzae* was observed for compound **12p**, which carries a trifluoromethyl group in the pyrimidine ring and a 3-chlorobenzyl unit.

**Figure 13:**
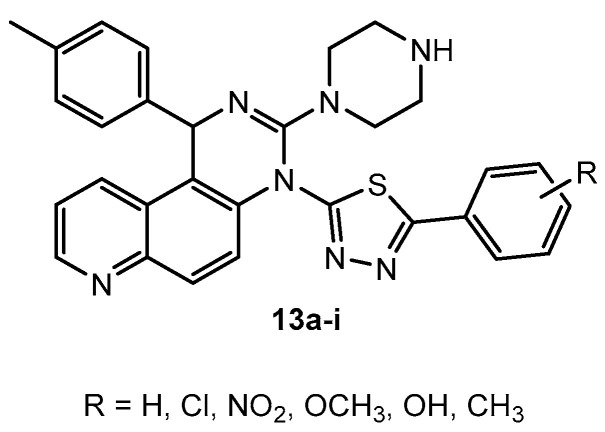
The structure of 2-(pyrido[3,2-*f*]quinazolin-4(1*H*)-yl)-1,3,4-thiadiazole derivatives.

Panwar and his coworkers obtained derivatives of [2-phenyl-1-(*p*-tolyl)pyrido[3,2-*f*]quinazolin-4(1*H*)-yl]-1,3,4-thiadiazolyl]-4-piperazine (**13a**–**i**, Figure 13) [52]. The compounds were tested for antibacterial activity against *Staphylococcus aureus*, *Escherichia coli*, and *Proteus vulgaris* at a concentration of 250 μg/mL. The results of the analysis are presented in Table 7. It was observed that chloro-substituted thiadiazoles **13b**–**d** exhibited stronger antimicrobial activity compared to other derivatives bearing nitro, methoxy, hydroxy, or methyl groups. Among the isomeric chloro-derivatives, the 2-chlorophenyl substitution was particularly favorable for antimicrobial potency. In summary, the synthesized compounds exhibited moderate antibacterial potential, although lower than that of the standard, Ampicillin trihydrate.

**Figure 14:**
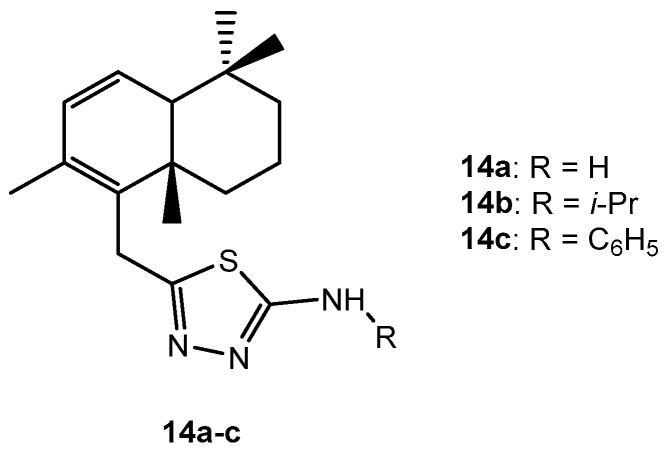
The structure of tetranorlabdane-1,3,4-thiadiazole hybrid.

Blaja et al. obtained tetranorlabdane compounds bearing 1,3,4-thiadiazole units (**14a**–**c**, Figure 14) [53]. The compounds were tested against two bacterial strains: the Gram-positive *Bacillus polymyxa* and the Gram-negative *Pseudomonas aeruginosa* (Table 8). The results indicate that compound **14a**, containing free amino group adjacent to 1,3,4-thiadiazole ring, possesses significant antibacterial activity, with an MIC value of 2.5 μg/mL.

**Figure 15:**
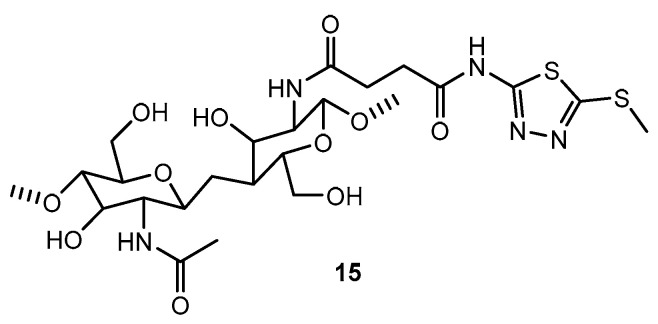
The structure of functionalized chitosan with thio-thiadiazole scaffold.

Ibrahim et al. synthesized a modified chitosan–thiadiazole conjugate (**15**, Figure 15) [54]. The antibacterial properties of this derivative were investigated against various pathogens, including *Escherichia coli*, *Pseudomonas aeruginosa*, *Bacillus subtilis*, and *Staphylococcus aureus*. Zones of inhibition for the individual bacterial strains, measured at a concentration of 50 μg/mL, are presented in Table 9. The synthesized compound exhibited moderate antibacterial potential.

**Figure 16:**
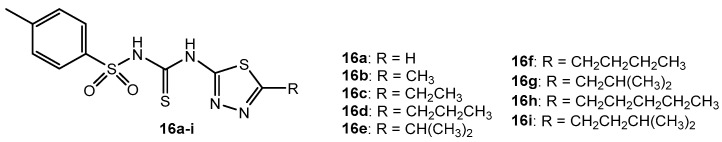
The structure of 1,3,4-thiadiazole sulfonyl thiourea derivatives.

Dinh Thanh et al. obtained a series of thioureas containing a 1,3,4-thiadiazole moiety (**16a**–**i**, Figure 16) [55]. The compounds were tested against Gram-positive bacteria (*Bacillus subtilis*, *Clostridium difficile*, *Staphylococcus aureus*, *Staphylococcus epidermidis*, *Streptococcus pneumoniae*) and Gram-negative bacteria (*Escherichia coli*, *Klebsiella pneumoniae*, *Pseudomonas aeruginosa*, *Salmonella typhimurium*). The results of the analysis are presented in Table 10. Almost all of the thioureas exhibited remarkable antibacterial activity. Among the studied compounds, **16a**, unsubstituted at the 5 position of the 1,3,4-thiadiazole ring, **16h**, bearing a pentyl substituent at the R site, and **16i**, with an isopentyl substituent, were the most effective inhibitors against *Staphylococcus aureus*, with MIC values ranging from 0.78 to 3.125 μg/mL, in comparison to the standards Ciprofloxacin and Vancomycin. Structure–activity relationship analysis led to the general conclusion that the presence of short alkyl chains, such as methyl or ethyl (**16b**, **16c**), is beneficial and results in good or moderate activity against many bacterial strains. It was also observed that chain elongation at the 5 position of the 1,3,4-thiadiazole ring increased activity, except for the *n*-butyl group (**16f**), which was completely inactive. In particular, compounds bearing five-carbon atom substituents (**16h**, **16i**) exhibited very strong activity against several Gram-positive strains (*Staphylococcus aureus*, *Staphylococcus epidermidis*, *Clostridium difficile*) and Gram-negative strains (*Klebsiella pneumoniae*, *Pseudomonas aeruginosa*). Chain branching did not strongly affect overall activity but influenced the susceptibility of specific strains.

**Figure 17:**
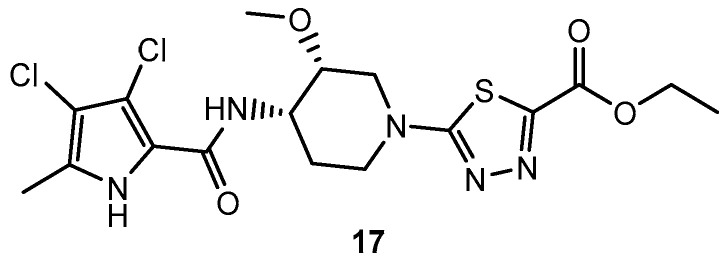
The derivative of 1,3,4-thiadiazole connected to pyrroloamide.

Zhao et al. obtained a series of pyrrolamide derivatives, one of which contained a 1,3,4-thiadiazole ring (**17**, Figure 17) [56]. The compound was tested for its inhibitory effect against *Staphylococcus aureus* (Gyrase IC_50_ = 0.137 μmol/L) and *Escherichia coli* (Gyrase IC_50_ = 6.87 μmol/L). For the investigated thiadiazole **17**, the MIC values were 0.125 μg/mL and 16 μg/mL, respectively.

**Figure 18:**
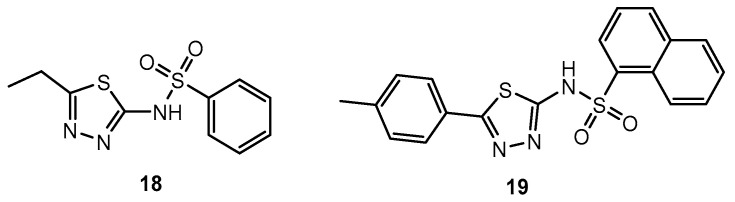
The structure of the ligands containing 1,3,4-thiadiazole moiety.

Hangan et al. obtained copper complexes of 1,3,4-thiadiazole derivatives bearing dimethylformamide (DMF) or 1,10-phenanthroline (phen) ligands (**18**, **19**, Figure 18), with the formulas [Cu_4_(**18**)_4_(OH)_4_(DMF)_2_(H_2_O)] and [Cu(**19**)_2_(phen)(H_2_O)] [57]. The biological activity of the heterocyclic ligands was evaluated against four Gram-positive bacteria (Methicillin-susceptible *Staphylococcus aureus* (MSSA), Methicillin-resistant *Staphylococcus aureus* (MRSA), *Staphylococcus lentus*, and *Enterococcus faecium*) and two Gram-negative bacteria (*Escherichia coli* and *Pseudomonas aeruginosa*) (Table 11). Compound **18** exhibited antibacterial activity against *Staphylococcus aureus* (MRSA), *Staphylococcus aureus* (MSSA), and *Escherichia coli*; however, it showed no activity against *Enterococcus faecium*, *Pseudomonas aeruginosa*, or *Staphylococcus lentus*. Coordination of compound **18** with Cu^2+^ ions led to a reduction in MIC to 2 µM/L. Compound **19** showed activity against all tested bacterial strains, and its transformation into a copper complex resulted in a significant reduction in MIC values.

**Figure 19:**
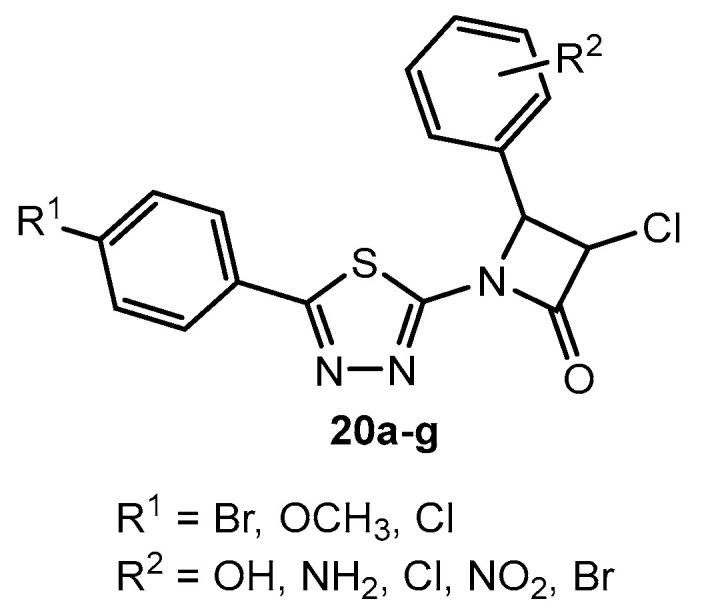
Azetidin-2-one derivatives of 1,3,4-thiadiazole ring.

Kumar et al. obtained a series of (4-substituted-phenyl-1,3,4-thiadiazol-2-yl)-4-(4-substituted-phenyl)azetidin-2-one derivatives (**20a**–**g**, Figure 19) [58]. The newly synthesized compounds were screened for antibacterial activity against *Pseudomonas aeruginosa*, *Staphylococcus aureus*, *Klebsiella pneumoniae*, *Enterococcus faecalis*, and *Escherichia coli* (Table 12). Results of the minimum bactericidal concentration (MBC) test indicated that the compounds exhibited moderate antibacterial potential. The authors demonstrated that the type and position of substituents on the phenyl rings of 4-substituted phenyl-1,3,4-thiadiazol-2-yl)-4-(4-substituted-phenyl)azetidin-2-one derivatives significantly influenced their antimicrobial activities. Electron-withdrawing groups (EWGs), such as chloro or nitro at the *para* position, enhanced antimicrobial activity. The best results were obtained for derivative **20g**, containing bromine at the R^1^ site and chlorine at the *para* position of the benzene ring (R^2^), which demonstrated notable activity against both Gram-positive and Gram-negative bacteria.

**Figure 20:**
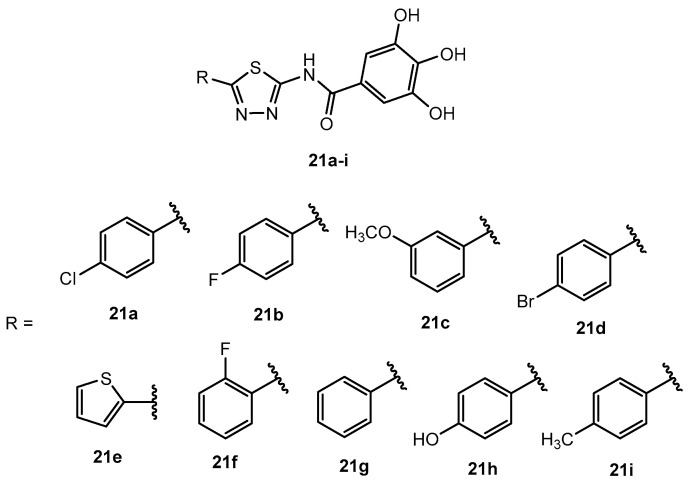
Gallic acid amide derivatives containing a 1,3,4-thiadiazole ring.

Liu’s group obtained a series of gallic acid amide derivatives containing a 1,3,4-thiadiazole core (**21a**–**i**, Figure 20) [59]. The compounds were tested for biological activity against *Vibrio harveyi* (Table 13). Among the described compounds, derivative **21b**, containing a 4-fluorophenyl group adjacent to the 1,3,4-thiadiazole ring, exhibited the most promising activity, with an MIC value of 0.0313 mg/mL.

**Figure 21:**
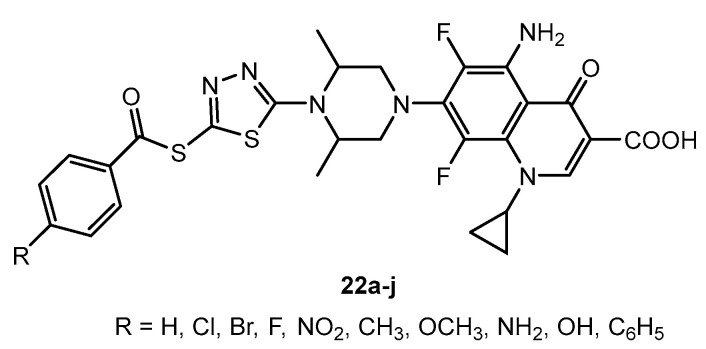
Sparfloxacin derivatives containing a 1,3,4-thiadiazole ring.

Gurunani et al. obtained Sparfloxacin derivatives containing a 1,3,4-thiadiazole ring (**22a**–**j**, Figure 21) [60]. The compounds were tested for activity against Gram-negative and Gram-positive bacteria (*Pseudomonas aeruginosa*, *Escherichia coli*, *Staphylococcus aureus*, and *Bacillus subtilis*), as well as *Mycobacterium tuberculosis* (Table 14). Almost all compounds synthesized by the authors exhibited moderate to good antibacterial activity. The highest MIC values against Gram-negative bacteria were observed for derivatives **22b**, **22e**, and **22j**, bearing chlorine, nitro, and phenyl groups, respectively.

**Figure 22:**
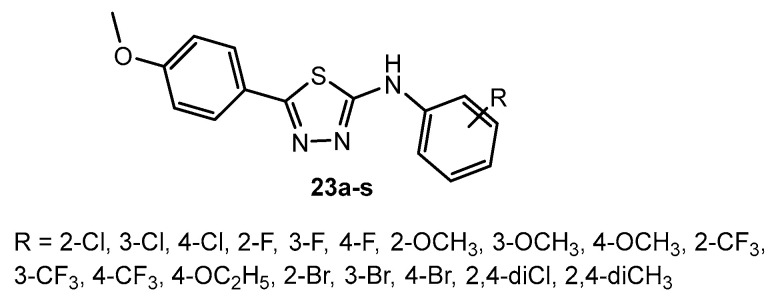
The derivatives of 5-(4-methoxyphenyl)-1,3,4-thiadiazole.

Wujec and coworkers obtained a series of 1,3,4-thiadiazole derivatives (**23a**–**s**, Figure 22) [61]. Almost all of the synthesized compounds exhibited no or only negligible antibacterial activity against Gram-positive and Gram-negative bacteria, except for compound **23p**, which contains a 4-bromophenyl substituent. This compound showed activity against *Staphylococcus epidermidis* with an MIC value of 31.25 µg/mL and *Micrococcus luteus* with an MIC value of 15.63 µg/mL.

**Figure 23:**
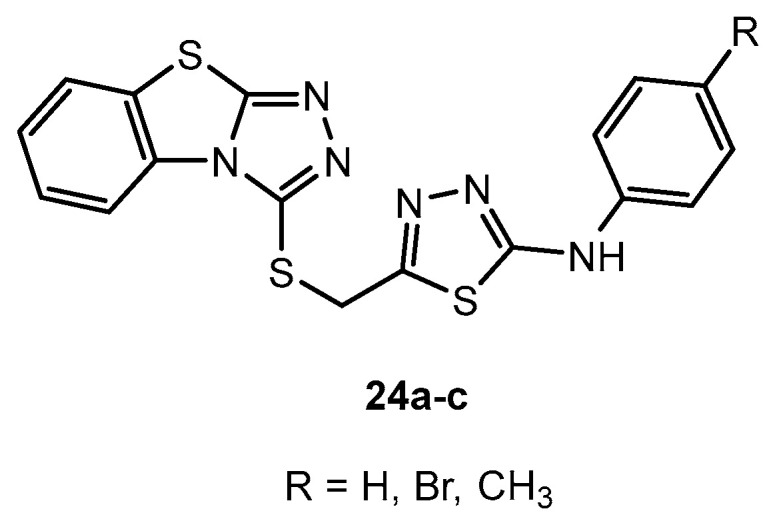
*S*-Mercaptotriazolebenzothiazole-based derivatives of 1,3,4-thiadiazole.

Alqahtani et al. obtained a series of compounds bearing a benzothiazolotriazole scaffold connected to a 1,3,4-thiadiazole ring (**24a**–**c**, Figure 23) [62]. The synthesized compounds were evaluated for their antibacterial activity against a panel of bacteria, including *Staphylococcus aureus*, *Escherichia coli*, *Klebsiella pneumoniae*, *Pseudomonas aeruginosa*, *Proteus mirabilis*, and *Acinetobacter baumannii*. Among the obtained compounds, only derivative **24b** (R = Br) showed moderate activity against *Staphylococcus aureus*, with an MIC value of 128 µg/mL.

**Figure 24:**
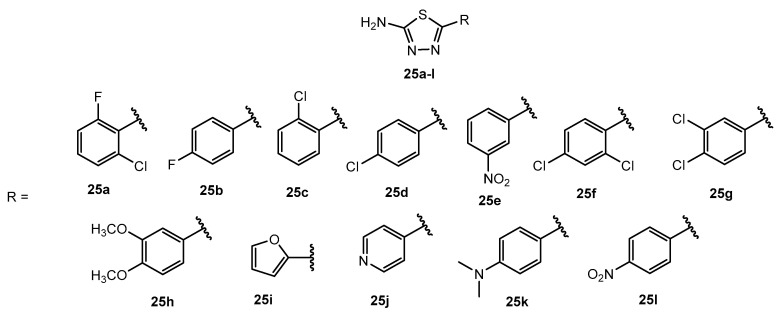
2-Amino-1,3,4-thiadiazole derivatives.

Pham’s group synthesized a series of 5-substituted-2-amino-1,3,4-thiadiazole derivatives (**25a**–**l**, Figure 24) [63]. The compounds were tested against *Escherichia coli*, *Pseudomonas aeruginosa*, *Streptococcus faecalis*, Methicillin-resistant strains of *Staphylococcus aureus*, and Methicillin-susceptible strains of *Staphylococcus aureus*. The compounds exhibited weak activity against the tested strains, with MIC values ranging from 126 to 1024 µg/mL.

**Figure 25:**
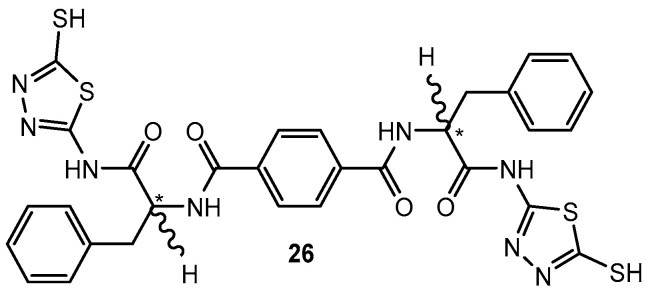
Chiral derivative of 1,3,4-thiadiazole.

Baddi et al. obtained a derivative of 1,3,4-thiadiazole (**26**, Figure 25), occurring in the form of two *L*/*D* isomers [64]. Both isomers were tested against Gram-positive (*Bacillus subtilis* and *Staphylococcus aureus*) and Gram-negative (*Pseudomonas aeruginosa* and *Escherichia coli*) bacteria. The hydrogel derived from the *D*-**26** isomer exhibited greater antibacterial activity than the *L*-**26** hydrogel, with zones of inhibition measuring 35 mm, 27.5 mm, and 24 mm for *Bacillus subtilis*, *Staphylococcus aureus*, and *Pseudomonas aeruginosa*, respectively.

**Figure 26:**
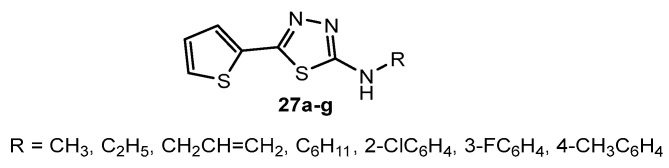
1,3,4-thiadiazole derivatives substituted with thiophene ring.

Muğlu et al. synthesized a range of 1,3,4-thiadiazole derivatives substituted with a thiophene ring (**27a**–**g**, Figure 26) [65]. The obtained compounds were tested against Gram-negative bacteria (*Escherichia coli*) and Gram-positive bacteria (*Staphylococcus aureus*, *Bacillus cereus*, *Bacillus subtilis* (6051), *Bacillus subtilis* (6633)). Among the tested compounds, only derivatives **27a** (R = CH_3_) and **27f** (R = 3-FC_6_H_4_) exhibited antibacterial activity, compound **27a** against both Gram-positive and Gram-negative bacteria and compound **27f** against Gram-positive bacteria only. Zones of inhibition at a concentration of 256 μg/mL for compound **27a** against *Bacillus subtilis* (6633), *Bacillus subtilis* (6051), *Staphylococcus aureus*, *Bacillus cereus*, and *Escherichia coli* were 14 mm, 13 mm, 14 mm, 15 mm, and 22 mm, respectively. For compound **27f**, the inhibition zones were 16 mm, 14 mm, 15 mm, and 15 mm, respectively.

**Figure 27:**
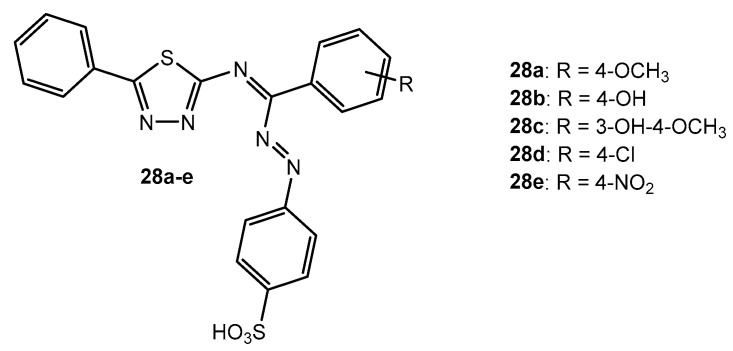
The structure of 5-phenyl-1,3,4-thiadiazole azo dyes.

Sunitha et al. obtained a series of azo-imine thiadiazoles (**28a**–**e**, Figure 27) [66]. The compounds were tested against Gram-positive bacteria (*Bacillus subtilis* and *Staphylococcus aureus*) and Gram-negative bacteria (*Escherichia coli* and *Klebsiella pneumoniae*), with Streptomycin as the standard drug (Table 15). Among the synthesized dyes, compound **28b** bearing a hydroxyl group at the *para* position of the benzene ring, **28d** with chlorine at the same position, and **28e** containing a nitro group showed good activity against *Escherichia coli*, with zones of inhibition of 13 mm, 12 mm, and 14 mm, respectively. In addition, compound **28b** was also active against *Bacillus subtilis*.

**Figure 28:**
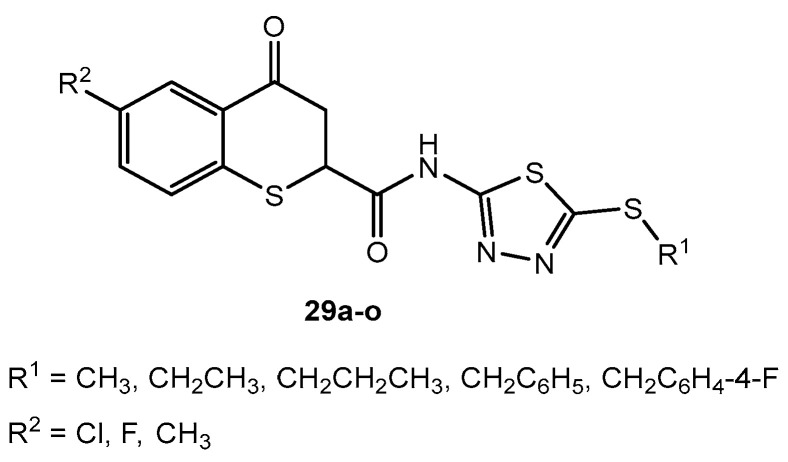
The structure of 1,3,4-thiadiazole thioether derivatives.

Yu et al. obtained a series of thiochroman-4-one derivatives incorporating carboxamide and 1,3,4-thiadiazole thioether moieties (**29a**–**o**, Figure 28) [67]. The compounds were tested against *Xanthomonas oryzae pv. oryzae* and *Xanthomonas axonopodis pv. citri* using Bismerthiazol and Thiodiazole copper (**J**, Figure 2) as standard drugs (Table 16). Compounds **29a**–**g** exhibited 74–100% and 60–94% in vitro antibacterial activity against *Xanthomonas oryzae pv. oryzae* at concentrations of 200 and 100 μg/mL, respectively. Meanwhile, compounds **29a**–**h** demonstrated 60–90% and 48–78% in vitro antibacterial activity against *Xanthomonas axonopodis pv. citri* at the same concentrations, both exceeding the activity of the Bismerthiazol and Thiodiazole copper standards.

**Figure 29:**
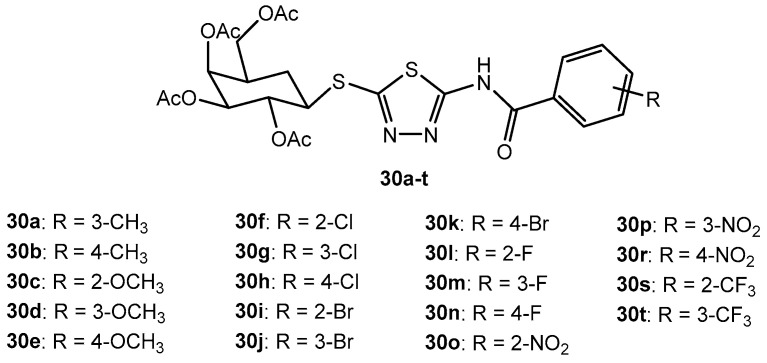
The structure of galactosides containing 1,3,4-thiadiazole moiety.

Shu et al. obtained a series of galactoside derivatives containing a 1,3,4-thiadiazole moiety (**30a**–**t**, Figure 29) [68]. The compounds were tested against *Xanthomonas oryzae pv. oryzae* and *Xanthomonas axonopodis pv. citri* at concentrations of 200 and 100 μg/mL. The inhibition rates ranged from 31.5% to 64.2% and 40.8% to 57.7% against *Xanthomonas oryzae pv. Oryzae* and from 18.3% to 36.2% and 19.8% to 36.1% against *Xanthomonas axonopodis pv. citri*, respectively. These values were lower than those observed for the Thiodiazole copper standard (**J**, Figure 2) (70.1%, 43.6%, 80.2%, and 46.1%).

**Figure 30:**
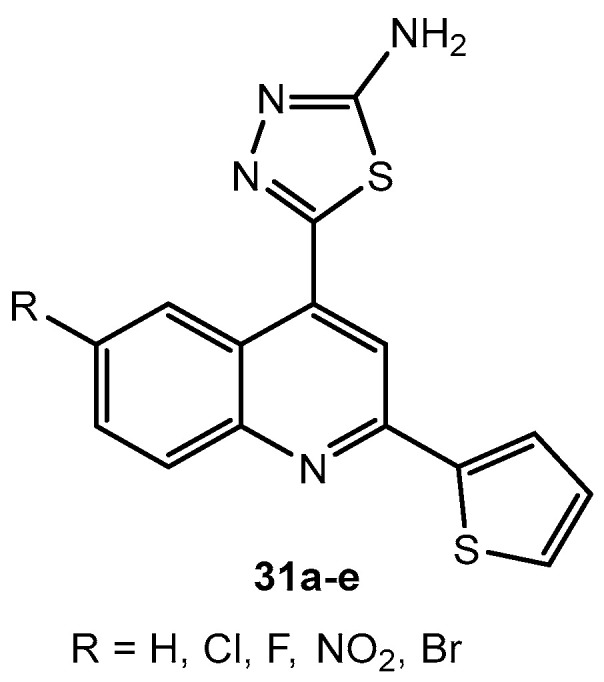
The structure of 1,3,4-thiadiazole amine derivatives.

Prasad et al. obtained a series of quinoline-bridged thiophenes connected to a 1,3,4-thiadiazole ring (**31a**–**e**, Figure 30) [69]. The compounds were tested against *Escherichia coli* and *Staphylococcus aureus*. The study found that all synthesized compounds (**31a**–**e**) exhibited notable antibacterial activity against both *Escherichia coli* and *Staphylococcus aureus*, although their activity was weaker than that of the Chloramphenicol standard.

**Figure 31:**
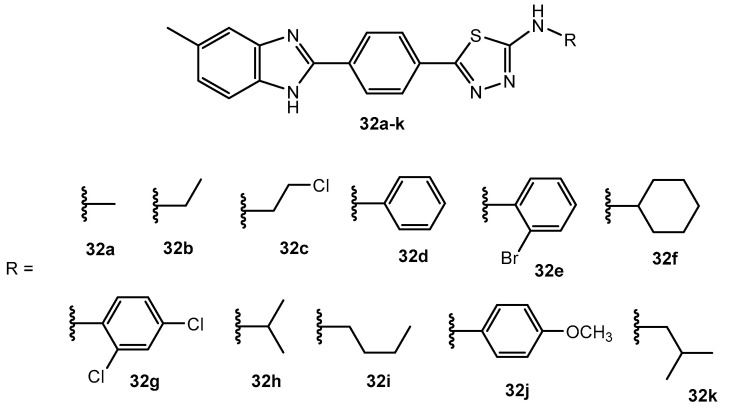
The structure of benzimidazole-1,3,4-thiadiazole conjugates.

Acar Çevik et al. obtained a series of benzimidazole derivatives containing a 1,3,4-thiadiazole scaffold (**32a**–**k**, Figure 31) [70]. The antibacterial activity of all compounds was evaluated by determining their minimum inhibitory concentration (MIC) against *Escherichia coli*, *Klebsiella pneumoniae*, *Pseudomonas aeruginosa*, *Enterococcus faecalis*, *Bacillus subtilis*, and *Staphylococcus aureus* (Table 17). Compounds **32f** and **32i** exhibited the greatest antibacterial activity against *Escherichia coli*, with MIC values below 0.97 µg/mL. In their search for structure–activity relationships, the authors found that antibacterial activity increased in compounds bearing alkylamine substituents at the 5 position of the thiadiazole ring, with longer chain or cyclic substituents (*n*-butyl, cyclohexyl) conferring higher potency. Among other derivatives exhibiting activity against the *Escherichia coli* strain, compound **32h**, containing an isopropyl group (MIC = 1.95 µg/mL), as well as compounds **32j** and **32k**, bearing a *p*-methoxyphenyl substituent and an isobutyl substituent, respectively, should be highlighted (MIC = 3.90 µg/mL). For the *Enterococcus faecalis* strain, compounds **32d**, **32i**, and **32k**, containing phenyl, *n*-butyl, and isobutyl groups, respectively, were the most active (MIC = 3.90 µg/mL).

**Figure 32:**
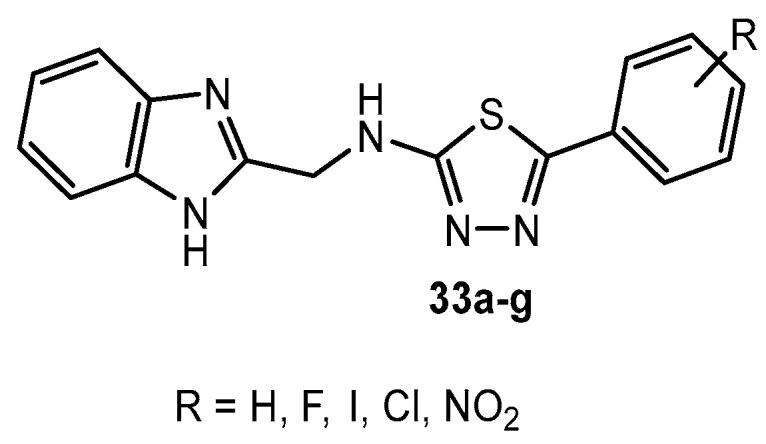
Benzimidazole derivatives containing a 1,3,4-thiadiazole ring.

Rdaiaan’s group obtained a series of 5-phenyl-1,3,4-thiadiazole derivatives containing a benzimidazole scaffold (**33a**–**g**, Figure 32) [71]. The derivatives were tested for antibacterial activity against Gram-positive (*Staphylococcus aureus*, *Staphylococcus epidermidis*) and Gram-negative (*Pseudomonas aeruginosa*, *Escherichia coli*) bacteria. Activity data at a concentration of 25 mg/mL are presented in Table 18. The synthesized compounds exhibited moderate to good antibacterial activity against these bacterial strains.

**Figure 33:**
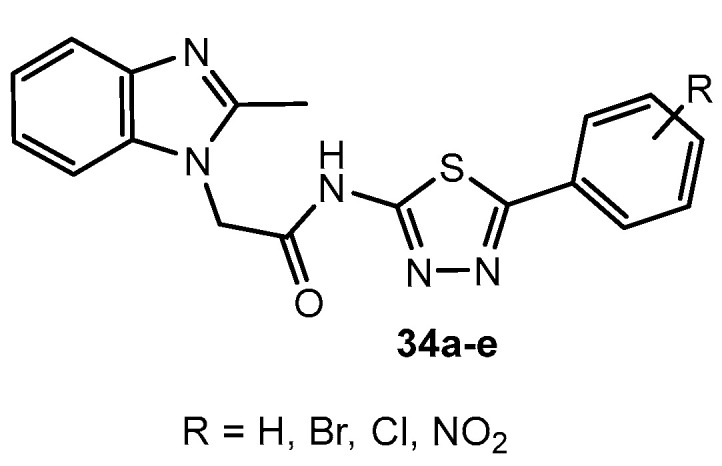
A series of benzimidazoles containing a 1,3,4-thiadiazole ring.

The same group of authors also obtained another series of benzimidazole derivatives containing a 1,3,4-thiadiazole ring (**34a**–**e**, Figure 33) [72]. The compounds were tested for antibacterial activity against Gram-positive (*Staphylococcus aureus*, *Staphylococcus epidermidis*) and Gram-negative (*Pseudomonas aeruginosa*, *Escherichia coli*) bacteria. Biological activity data, represented by zones of inhibition measured at a concentration of 100 mg/mL, are presented in Table 19.

**Figure 34:**
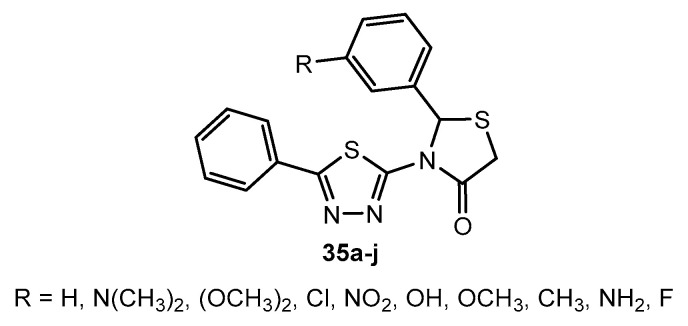
Derivatives of 1,3,4-thiadiazole attached to thiazolidinone core.

Garg and coworkers obtained derivatives of 1,3,4-thiadiazole attached to 2,3-disubstituted thiazolidinones (**35a**–**j**, Figure 34) [73]. The compounds were tested for antibacterial activity against Gram-positive (*Staphylococcus aureus*, *Streptococcus pyogenes*) and Gram-negative (*Pseudomonas aeruginosa*, *Escherichia coli*) bacteria. The results of the zone of inhibition assay at a concentration of 250 μg/mL are presented in Table 20. Biological screening showed that all tested compounds were active against all strains. Some of the compounds—**35a** (R = H), **35b** (R = N(CH_3_)_2_), **35f** (R = OH), and **35i** (R = NH_2_)—exhibited activity comparable to the standard drug Ampicillin. The authors concluded that modifying the substitution at the distal phenyl ring attached to the thiazolidinone at the 2 position enhances antimicrobial activity. This effect is likely attributable to increased lipophilicity, which facilitates permeation through microbial lipid membranes. Overall, structural features, such as thiazolidinone–thiadiazole conjugation, appropriate aromatic substitution, and lipophilic character, contribute to superior antimicrobial potency compared with reference drugs.

**Figure 35:**
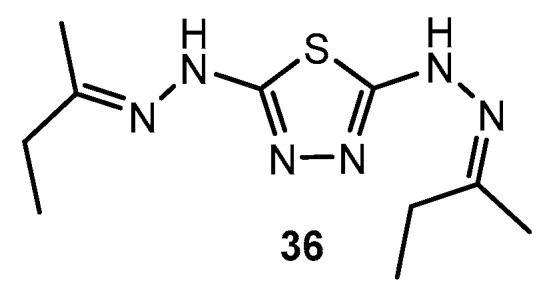
The structure of a 2,5-dihydrazinyl-1,3,4-thiadiazole ligand.

Weaam prepared three metal complexes containing a 2,5-dihydrazinyl-1,3,4-thiadiazole ligand coordinated to chromium, cobalt, and copper atoms (**36**, Figure 35) [74]. The compounds were tested for antibacterial activity against *Escherichia coli* and *Staphylococcus aureus*. The results obtained from the analysis are presented in Table 21. The synthesized complexes exhibited biological activity comparable to that of Ciprofloxacin, which was used as the standard.

**Figure 36:**
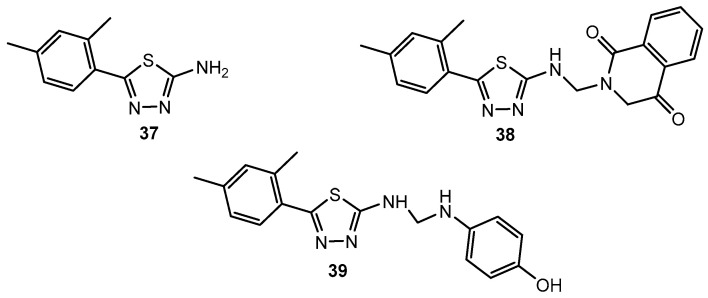
The structure of 5-(2,4-dimethylphenyl)-1,3,4-thiadiazol-2-amine (**37**) and its derivatives.

Gidwani’s group obtained three derivatives of 2-amino-1,3,4-thiadiazole (**37**–**39**, Figure 36) [75]. The compounds were tested for antibacterial activity against Gram-positive (*Bacillus subtilis*) and Gram-negative (*Escherichia coli*) bacteria. Compounds **37** and **38** exhibited MIC values of 1000 μg/mL against *Bacillus subtilis*, whereas among the tested compounds only **38** showed activity against *Escherichia coli*, also with an MIC of 1000 μg/mL. None of the synthesized compounds demonstrated superior activity compared to the standard drug Ciprofloxacin (MIC = 25 μg/mL) against the tested microbial strains.

**Figure 37:**
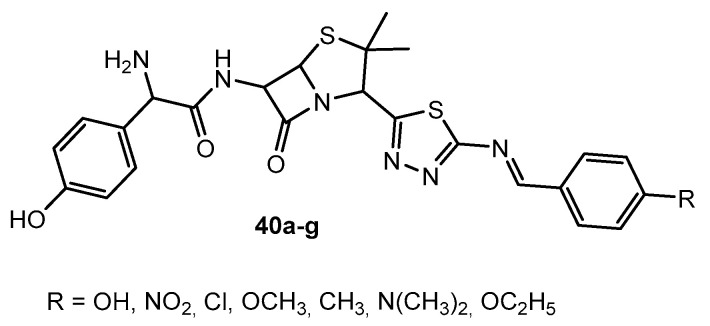
Amoxicillin derivatives containing a 1,3,4-thiadiazole moiety.

Nawar et al. synthesized 1,3,4-thiadiazole derivatives containing the Amoxicillin scaffold (**40a**–**g**, Figure 37) [76]. The compounds were tested for antibacterial activity against Gram-negative (*Proteus mirabilis* and *Escherichia coli*) and Gram-positive (*Mycobacterium tuberculosis*) bacteria (Table 22) and exhibited considerable zones of inhibition. The best result was observed for derivative **40a**, containing a hydroxyl group at the R site, which demonstrated greater activity than Amoxicillin.

**Figure 38:**
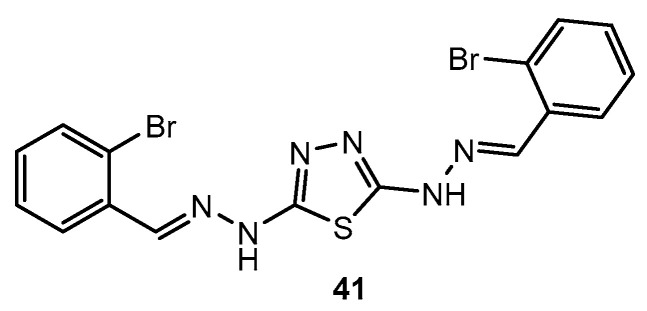
The structure of the obtained 1,3,4-thiadiazole ligand bearing 2-(2-bromobenzylidene)hydrazinyl groups.

Weaam also obtained metal complexes containing the 1,3,4-thiadiazole ligand (**41**, Figure 38) coordinated with iron, nickel, and copper atoms [77]. The complexes were tested for antibacterial activity against *Escherichia coli* and *Staphylococcus aureus*. The study data are presented in Table 23. The best activity against *Escherichia coli* was observed for the copper complex, while the iron and nickel complexes were most effective against *Staphylococcus aureus*. All of the obtained complexes exhibited greater antibacterial activity compared to the free 1,3,4-thiadiazole ligand.

**Figure 39:**
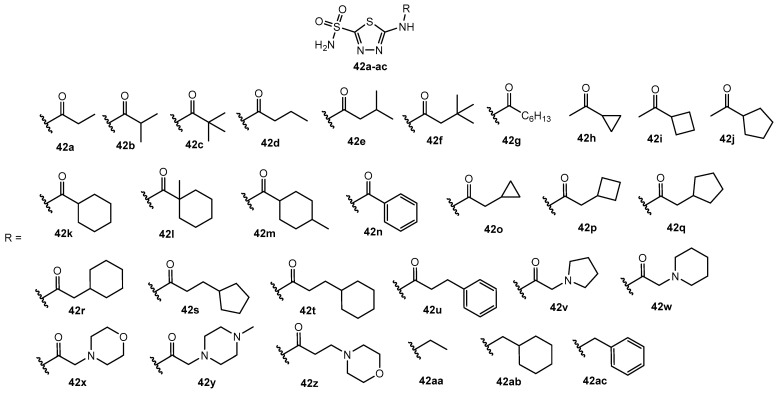
2-sulfonamide-1,3,4-thiadiazole derivatives.

Kaur et al. obtained a range of sulfonamides containing a 1,3,4-thiadiazole moiety (**42a**–**ac**, Figure 39) [78]. These compounds were screened for antibacterial activity against *Enterococcus faecium*. The MIC values for each of the tested derivatives are presented in Table 24. The authors noted a correlation between lipophilicity and activity; the greater the lipophilicity, the stronger the activity against *Enterococcus faecium*. Biological screening revealed that many of the synthesized compounds exhibited high antibacterial activity compared to the standard drug Acetazolamide. The most potent derivatives against *Enterococcus faecium* were **42t**, containing a 3-cyclohexylpropanoyl group at the R site, **42f**, bearing a 3-methylbutanoyl group at the same position, and **42g**, with a heptanoyl group, with MIC values of 0.007, 0.015 and 0.015 μg/mL, respectively. In the search for correlations between the structure of the synthesized sulfonamides and their biological activity, the authors found that in addition to lipophilic groups (alkyl and cycloalkyl), chain extension by a methylene linker can also dramatically increase potency. In contrast, the presence of heteroatoms in pendant groups was found to reduce activity.

**Figure 40:**
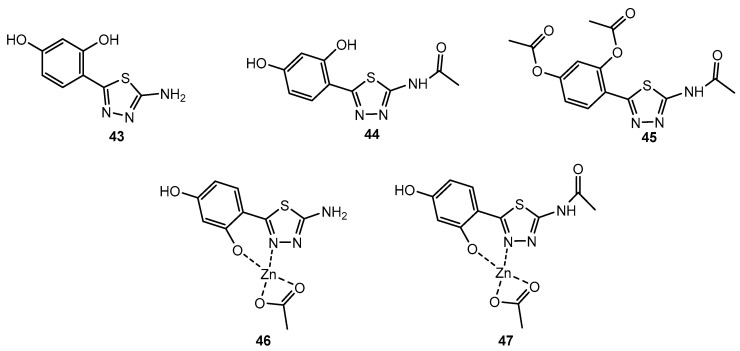
The structures of the obtained 1,3,4-thiadiazole ligands and their metal complexes.

Kracz et al. obtained derivatives of 1,3,4-thiadiazoles and their zinc complexes (**43**–**47**, Figure 40) [79]. The antibacterial properties against Gram-positive (*Staphylococcus aureus*) and Gram-negative (*Escherichia coli*, *Pseudomonas aeruginosa*) bacteria were evaluated by determining the MIC values (Table 25). The tested compounds exhibited low antibacterial efficacy. The study revealed that acylation of the amino and hydroxyl groups leads to a decrease in antibacterial activity.

**Figure 41:**
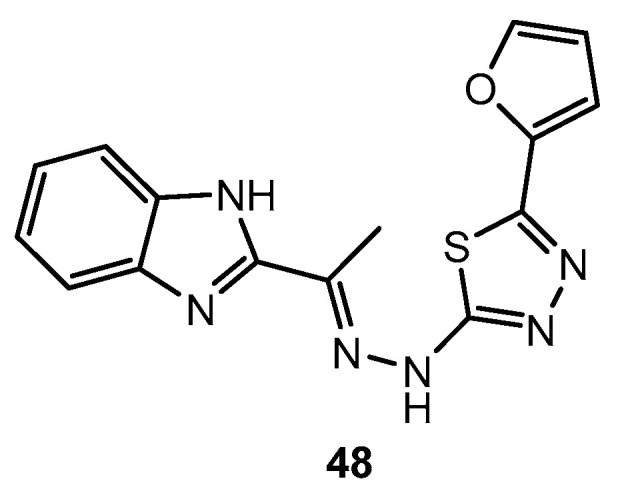
A 1,3,4-thiadiazole derivative containing furan and benzimidazole scaffolds.

Abdel-Motaal et al. obtained a benzimidazole-2-yl derivative of 1,3,4-thiadiazole containing a furan-2-yl substituent (**48**, Figure 41) [80]. The compound was tested for antibacterial activity against Gram-positive bacteria (*Staphylococcus aureus*), Gram-negative bacteria (*Escherichia coli*), and bacterial spores (*Bacillus pumilus*). The study demonstrated high activity of derivative **48** against the tested microorganisms, with zones of inhibition of 18.96 mm for *Staphylococcus aureus*, 18.20 mm for *Bacillus pumilus*, and 17.33 mm for *Escherichia coli*.

**Figure 42:**
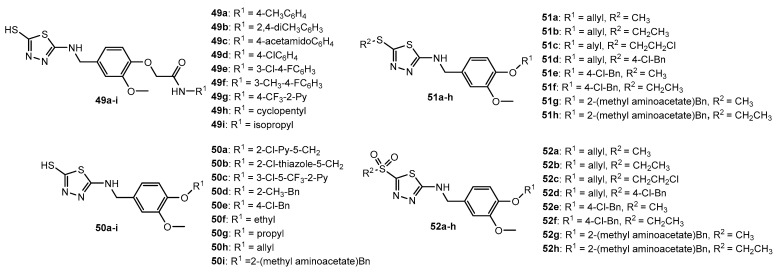
A series of vanillin derivatives containing a 1,3,4-thiadiazole moiety.

Scheme 1 thiadiazole moiety (**49a**–**i**, **50a**–**i**, **51a**–**h**, **52a**–**h**, Figure 42) [81]. The compounds were tested against two bacterial strains: *Xanthomonas oryzae pv. oryzae* and *Xanthomonas oryzae pv. oryzicola*. The EC_50_ values for the tested compounds are presented in Table 26. The strongest inhibitory activity against *Xanthomonas oryzae pv. oryzae* was exhibited by compounds **50c**, **50e**, **50h**, **50i**, **52a**, **52b**, and **52c**, with EC_50_ values of 38.74, 33.73, 33.25, 31.78, 16.03, 28.47, and 3.14 μg/mL, respectively. Against *Xanthomonas oryzae pv. oryzicola*, the most active compounds were **50h**, **50i**, **52a**, **52b**, and **52c**, with EC_50_ values of 35.49, 26.54, 27.69, 36.47, and 8.83 μg/mL, respectively. Among all tested compounds, **52c**, bearing an allyl group at the R^1^ site and a 2-chloroethyl group at the R^2^ site, exhibited the strongest inhibitory activity against both strains. The results indicated that in the case of thiol derivatives (**49a**–**i**, **50a**–**i**), modifications to the vanillin substituents (R^1^) had little impact on antibacterial activity. However, substituting hydrogen at the R^2^ position with an alkyl group significantly reduced activity, indicating that thiol derivatives (**50e**, **50h**, **50i**) are generally more potent than their corresponding thioether analogues (**51a**–**h**). Replacement of sulfur with a sulfone group enhanced activity for compounds bearing the same vanillin and R^1^ and R^2^ groups, suggesting that sulfone derivatives (**52a**–**h**) may interact covalently or through hydrogen bonding with protein targets, thereby leading to stronger antibacterial effects.

**Figure 43:**
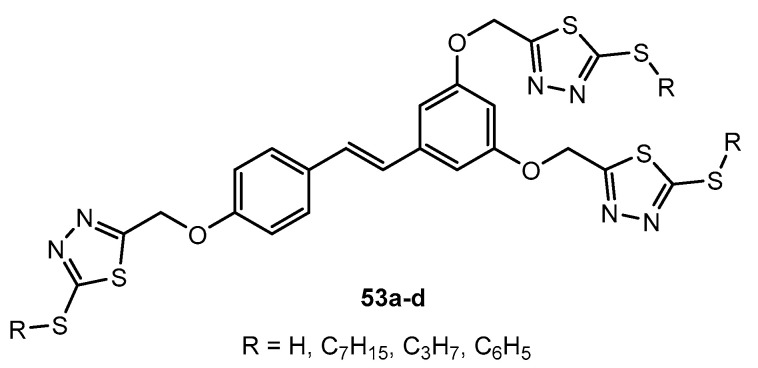
Resveratrol derivatives bearing a 1,3,4-thiadiazole moiety.

Ali et al. obtained 1,3,4-thiadiazole derivatives of Resveratrol (**53a**–**d**, Figure 43) [82]. The compounds were tested for antibacterial activity against Gram-positive (*Staphylococcus aureus*, *Streptococcus pyogenes*) and Gram-negative (*Escherichia coli*, *Klebsiella pneumoniae*) bacteria. The zones of inhibition for the compounds, measured at a concentration of 500 μg/mL, are presented in Table 27. The best results were obtained for compound **53c**, bearing propyl substituents, and **53d**, substituted with phenyl groups, which exhibited strong activity against *Staphylococcus aureus*. The unsubstituted derivative **53a** showed notable activity against *Escherichia coli*.

**Figure 44:**
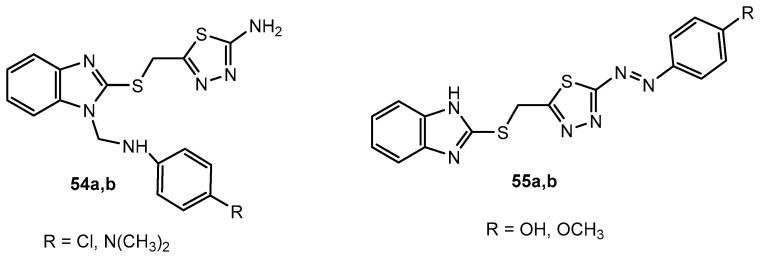
Benzimidazole-based compounds bearing a 1,3,4-thiadiazole moiety.

Mahmoud et al. synthesized a series of benzimidazole derivatives containing a 1,3,4-thiadiazole ring (**54a**, **54b**, **55a**, **55b**, Figure 44) [83]. The synthesized compounds were tested for antibacterial activity against *Staphylococcus aureus*, *Bacillus subtilis*, *Pseudomonas aeruginosa*, and *Escherichia coli*. Compounds **54a** and **54b** exhibited very good activity against all tested bacteria, with zones of inhibition ranging from 18 to 23 mm at a concentration of 100 mg/mL. Compound **55a** (R = OH) demonstrated excellent activity against *Escherichia coli* (25 mm) and good activity against *Staphylococcus aureus* (14 mm) and *Bacillus subtilis* (19 mm), also at a concentration of 100 mg/mL.

**Figure 45:**
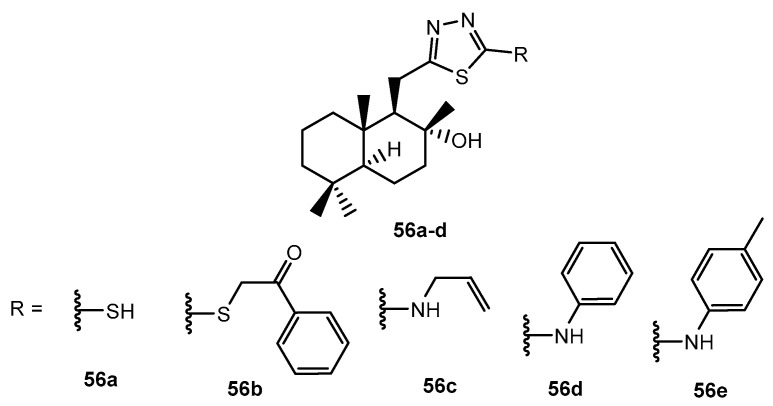
The structure of novel homodrimane sesquiterpenoids bearing 1,3,4-thiadiazole units.

Lungu et al. obtained a series of homodrimane sesquiterpene–thiadiazole hybrid compounds (**56a**–**e**, Figure 45) [84]. The synthesized derivatives were tested for antibacterial activity against Gram-positive *Bacillus subtilis* and Gram-negative *Pseudomonas aeruginosa* bacterial strains. The determined minimum inhibitory concentration (MIC) values revealed that compound **56a**, bearing a mercapto substituent at the 2 position of the 1,3,4-thiadiazole ring, and compound **56c**, containing an allylamino substituent, exhibited promising non-selective antibacterial activity, with MIC values of 0.094 and 0.5 µg/mL, respectively.

**Figure 46:**
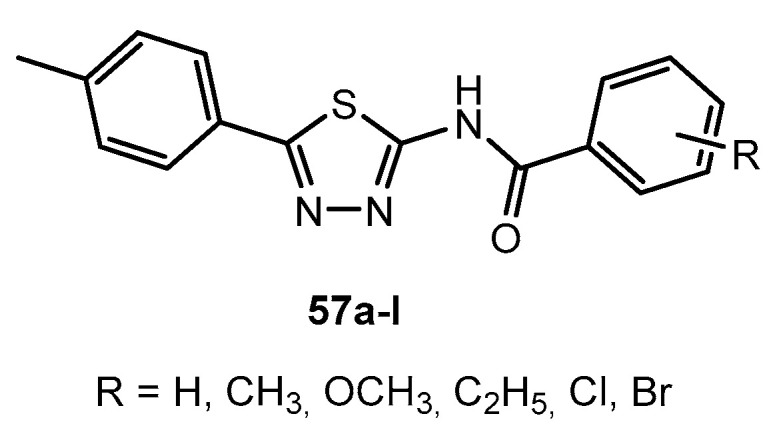
Structures of benzamide derivatives containing a 1,3,4-thiadiazole ring.

Pardeshi’s group synthesized a series of benzamide–thiadiazole-based derivatives substituted at the benzamide fragment with various aryl groups (**57a**–**l**, Figure 46) [85]. The compounds were tested for antibacterial activity against *Staphylococcus aureus*, *Bacillus subtilis*, *Escherichia coli*, and *Pseudomonas aeruginosa* (Table 28). The synthesized derivatives exhibited good antibacterial properties, and the standard drug Streptomycin was used for comparative purposes.

**Figure 47:**
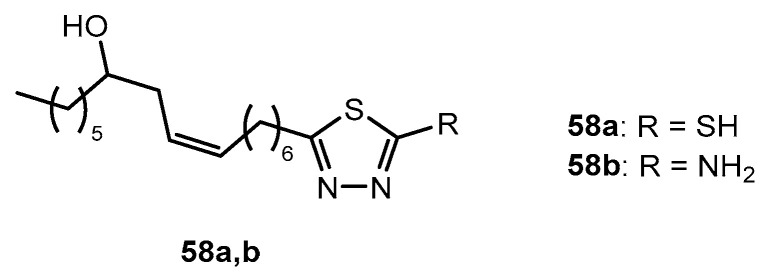
Ricinoleic acid derivatives bearing a 1,3,4-thiadiazole moiety.

Brahimi and coworkers obtained 1,3,4-thiadiazole derivatives of castor oil extract (**58a**, **58b**, Figure 47) [86]. The synthesized compounds were tested for their in vitro antimicrobial activity against Gram-positive bacteria (*Staphylococcus aureus*, *Enterococcus faecalis*, *Bacillus cereus*) and Gram-negative bacteria (*Pseudomonas aeruginosa*, *Escherichia coli*, *Klebsiella planticola*, *Salmonella*, *Proteus vulgaris*) using nutrient agar medium (Table 29). The compounds exhibited moderate antimicrobial properties, with the best result observed for derivative **58a**, containing a mercapto group adjacent to the 1,3,4-thiadiazole ring, which showed good activity against *Enterococcus faecalis* (12 mm) and outperformed the standard drug Ampicillin.

#### 2.1.2. Bicyclic 1,3,4-Thiadiazole Derivatives

Another notable group of derivatives exhibiting antibacterial activity comprises fused systems in which the 1,3,4-thiadiazole ring is incorporated into a bicyclic structure. Typical examples include fusion with aromatic or heteroaromatic rings, such as benzene, pyrazole, pyridine, or pyrrole.

**Figure 48:**
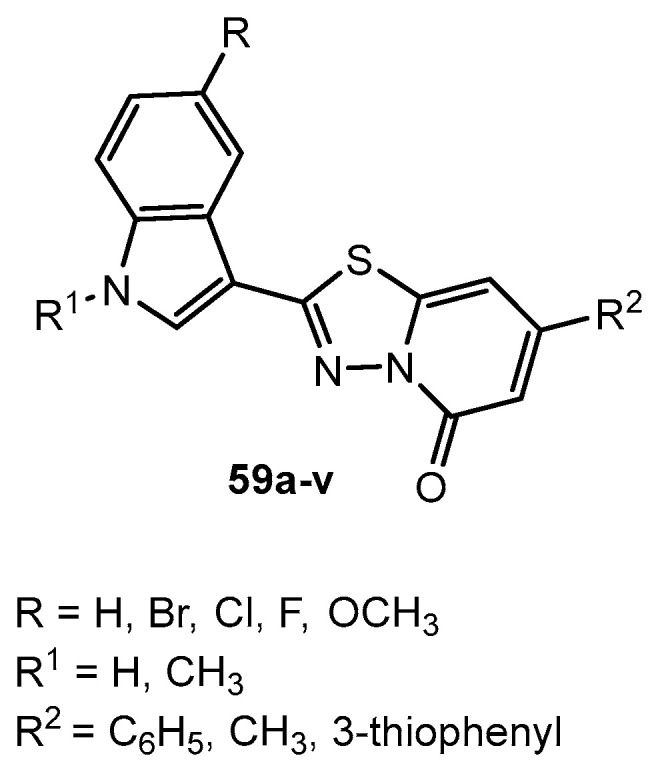
Derivatives of 1,3,4-thiadiazolo[3,2-*a*]pyrimidin-5-one.

Parrino’s group obtained a series of 1,3,4-thiadiazolo[3,2-*a*]pyrimidin-5-one derivatives (**59a**–**v**, Figure 48) [87]. The compounds were tested for biological activity against Gram-positive pathogens *Staphylococcus aureus* and *Enterococcus faecalis* and Gram-negative pathogens *Pseudomonas aeruginosa* and *Escherichia coli*. The best results against *Staphylococcus aureus* were observed for derivatives **59e** (R = F, R^1^ = H, R^2^ = C_6_H_5_, MIC = 50 μg/mL), **59f** (R = H, R^1^ = CH_3_, R^2^ = C_6_H_5_, MIC = 100 μg/mL), **59k** (R = H, R^1^ = H, R^2^ = CH_3_, MIC = 50 μg/mL), and **59l** (R = H, R^1^ = CH_3_, R^2^ = CH_3_, MIC = 50 μg/mL). Regarding activity against *Enterococcus faecalis*, the most promising compounds were **59j** (R = F, R^1^ = CH_3_, R^2^ = C_6_H_5_, MIC = 50 μg/mL) and **59k** (R = H, R^1^ = H, R^2^ = CH_3_, MIC = 25 μg/mL).

**Figure 49:**
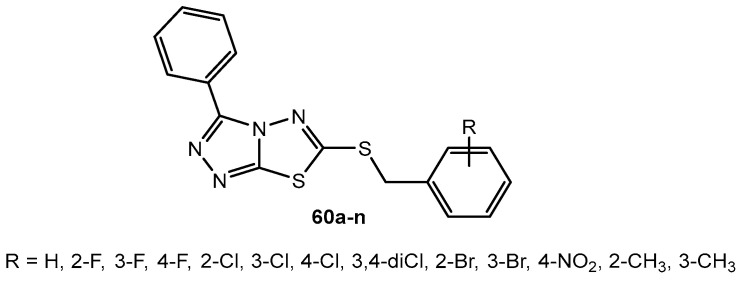
Derivatives of [1,2,4]triazolo[3,4-*b*][1,3,4]thiadiazole bearing aryl substituents.

Mahdavi’s group synthesized [1,2,4]triazolo[3,4-*b*][1,3,4]thiadiazole derivatives (**60a**–**n**, Figure 49) [88]. All compounds were tested against *Proteus mirabilis* and showed weak activity (MIC = 256 μg/mL) compared to the standard drug Ciprofloxacin (MIC = 0.25 μg/mL).

**Figure 50:**
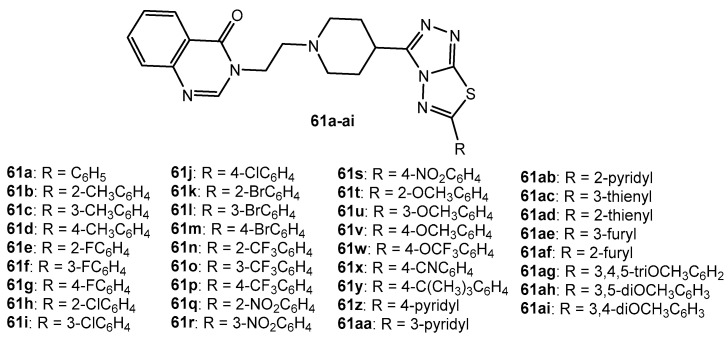
The structure of quinazolin-4(3*H*)-one 1,2,4-triazolo[3,4-*b*][1,3,4]thiadiazole hybrids.

Wu et al. synthesized a series of quinazolin-4(3*H*)-one derivatives containing the 1,2,4-triazolo[3,4-*b*][1,3,4]thiadiazole moiety (**61a**–**ai**, Figure 50) [89]. The compounds were tested for biological activity against four bacterial strains: *Xanthomonas axonopodis pv. citri*, *Xanthomonas oryzae pv. oryzicola*, *Xanthomonas oryzae pv. oryzae*, and *Pseudomonas syringae pv. actinidiae* at a concentration of 100 µg/mL, employing the commercial antibacterial agent BMT as the positive control (Table 30).

**Figure 51:**
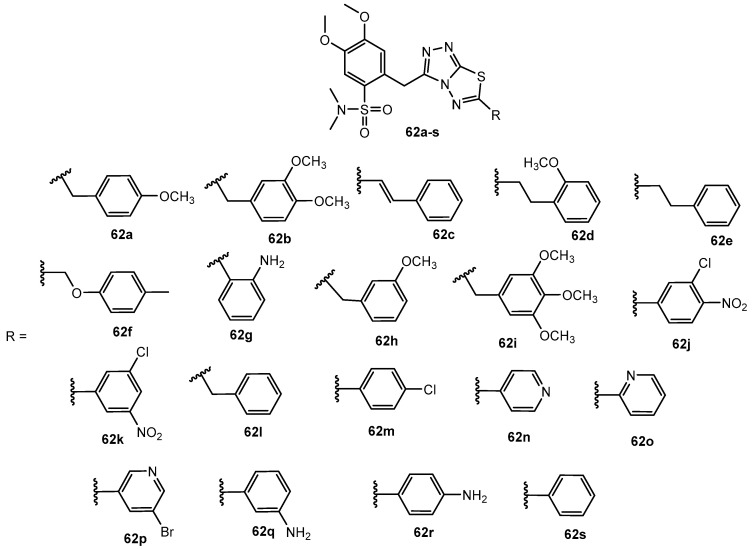
Triazolo-based thiadiazole derivatives.

Kamoutsis et al. obtained a range of fused 1,3,4-thiadiazole arrangements (**62a**–**s**, Figure 51) [90]. The obtained compounds were tested for activity against *Bacillus cereus*, *Staphylococcus aureus*, *Listeria monocytogenes*, *Pseudomonas aeruginosa*, *Escherichia coli*, and *Salmonella typhimurium*. The results of antibacterial analyses are presented in Table 31. The best results were obtained for the **62s** derivative, containing a phenyl group at the R site, characterized by MBC = 10–40 μg/mL and MIC = 5–20 μg/mL.

**Figure 52:**
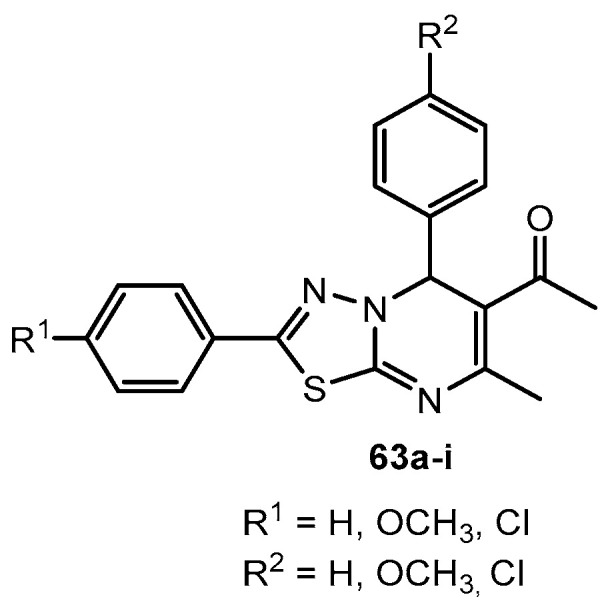
The structure of bicyclic 1,3,4-thiadiazole-pyrimidine derivatives.

Bhadraiah et al. obtained bicyclic 1,3,4-thiadiazolo[3,2-α]pyrimidine analogues (**63a**–**i**, Figure 52) [91]. The compounds were tested for bactericidal activity against Gram-positive (*Bacillus cereus*, *Staphylococcus aureus*) and Gram-negative (*Escherichia coli*, *Klebsiella pneumoniae*) bacterial strains. Biological tests revealed that derivative **63e**, containing two methoxy groups at the R^1^ and R^2^ sites, exhibited the best activity among the synthesized compounds, while derivatives **63b** and **63h** showed moderate properties (Table 32).

**Figure 53:**
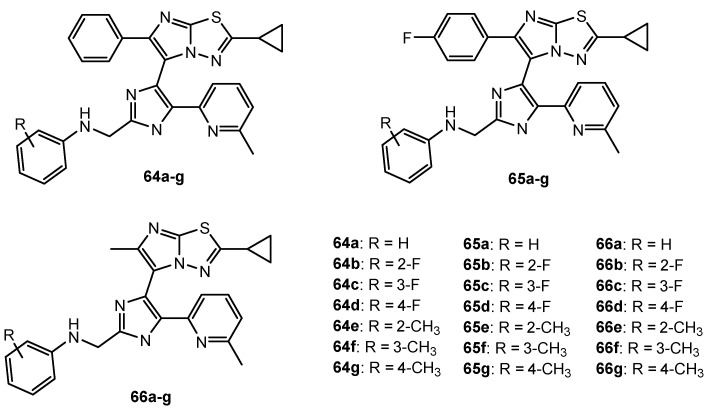
Compounds containing the imidazo[2,1-*b*][1,3,4]thiadiazole scaffold.

Jin’s group synthesized a series of fused imidazo[2,1-*b*][1,3,4]thiadiazole derivatives (**64a**–**g**, **65a**–**g**, **66a**–**g**, Figure 53) [92]. The obtained compounds were tested for antibacterial activity against *Staphylococcus aureus* (4220), *Staphylococcus aureus* (209), *Escherichia coli*, *Pseudomonas aeruginosa*, Methicillin-resistant *Staphylococcus aureus* (3167), and Quinolone-resistant *Staphylococcus aureus* (3505). The tested compounds exhibited no or low activity against Gram-positive and Gram-negative bacteria. However, selected derivatives demonstrated activity against multi-drug-resistant Gram-positive strains. Specifically, compounds **64c** (R = 3-F, MIC_50_ = 12.79 μg/mL), **64e** (R = 2-CH_3_, MIC_50_ = 17.84 μg/mL), **65e** (R = 2-CH_3_, MIC_50_ = 15.81 μg/mL), and **66a** (R = H, MIC_50_ = 18.49 μg/mL) showed moderate activity against Methicillin-resistant *Staphylococcus aureus*. In the case of Quinolone-resistant *Staphylococcus aureus*, moderate activity was observed for fluorine- or methyl-substituted compounds **64c**–**f** (MIC_50_ = 9.06–19.09 μg/mL), the unsubstituted compound **65a** (MIC_50_ = 16.69 μg/mL), the fluorine-substituted compound **65c** (MIC_50_ = 17.24 μg/mL), the methyl-substituted compound **65e** (MIC_50_ = 18.02 μg/mL), and fluorine- or methyl-substituted compounds **66c**–**g** (MIC_50_ = 12.98–17.44 μg/mL).

#### 2.1.3. Multi-Substituted 1,3,4-Thiadiazole Derivatives

Highly substituted 1,3,4-thiadiazole derivatives represent a less commonly explored class of compounds.

**Figure 54:**
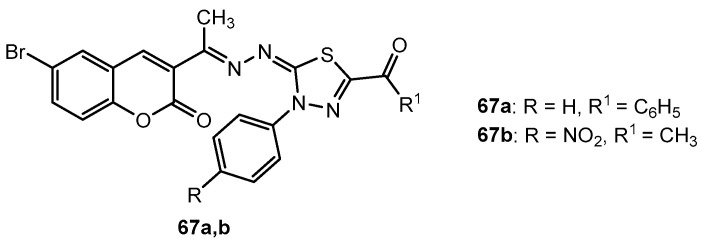
The structure of tri-substituted 1,3,4-thiadiazole derivatives bearing acyl, aryl, and 2*H*-chromen-2-one scaffolds.

Abdel-Aziem and coworkers obtained two coumarin-linked thiadiazole derivatives (**67a**,**b**, Figure 54) [93]. The newly synthesized derivatives were initially investigated for their antibacterial activity. Six pathogenic microbes were selected for assessment: *Bacillus pumilis* and *Streptococcus faecalis*, representing Gram-positive bacteria, and *Escherichia coli* and *Enterobacter cloacae*, representing Gram-negative bacteria. The standard antibacterial drugs Penicillin G (for Gram-positive bacteria) and Ciprofloxacin (for Gram-negative bacteria) were used as references (Table 33). The obtained compounds exhibited moderate biological activity in comparison with the standard drugs.

**Figure 55:**
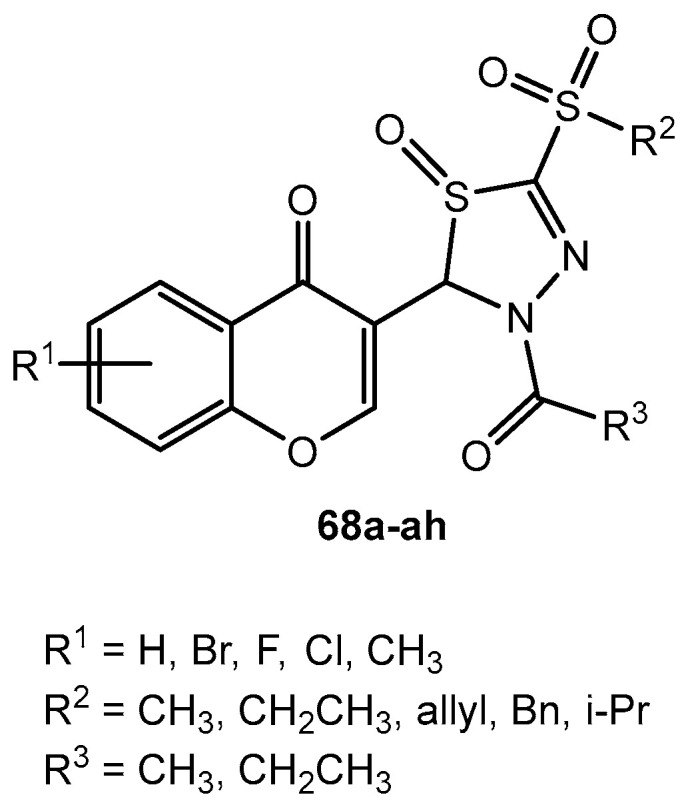
The structure of 5-sulfonyl-1,3,4-thiadiazole-substituted flavonoids.

Dai et al. obtained 5-sulfonyl-1,3,4-thiadiazole-substituted flavonoids (**68a**–**ah**, Figure 55) [94]. The compounds were tested for antibacterial activity against two bacterial strains: *Xanthomonas axonopodis pv. citri* and *Xanthomonas oryzae pv. oryzae*. The inhibition rates at a concentration of 50 μg/mL are presented in Table 34. Compounds **68a** (R^1^ = H, R^2^ = CH_3_, R^3^ = CH_3_), **68c** (R^1^ = 6-F, R^2^ = CH_3_, R^3^ = CH_3_), **68g** (R^1^ = H, R^2^ = CH_2_CH_3_, R^3^ = CH_3_), **68i** (R^1^ = 6-F, R^2^ = CH_2_CH_3_, R^3^ = CH_3_), **68m** (R^1^ = 6-Br, R^2^ = CH_2_CH_2_CH_3_, R^3^ = CH_3_), and **68n** (R^1^ = 6-F, R^2^ = CH_2_CH_2_CH_3_, R^3^ = CH_3_) exhibited remarkable antibacterial activity against *Xanthomonas oryzae pv. oryzae*, with EC_50_ values below 10 μg/mL, which were superior to that of Bismerthiazol (70.89 μg/mL). Preliminary structure–activity analysis indicated that the introduction of substituents at the 6 position of the flavonoid core significantly affected antibacterial activity. Introducing small groups, such as hydrogen or fluorine, at this position markedly increased potency. Alkyl substitutions on the sulfone group had minimal impact, whereas the presence of a benzyl group reduced antibacterial activity.

**Figure 56:**
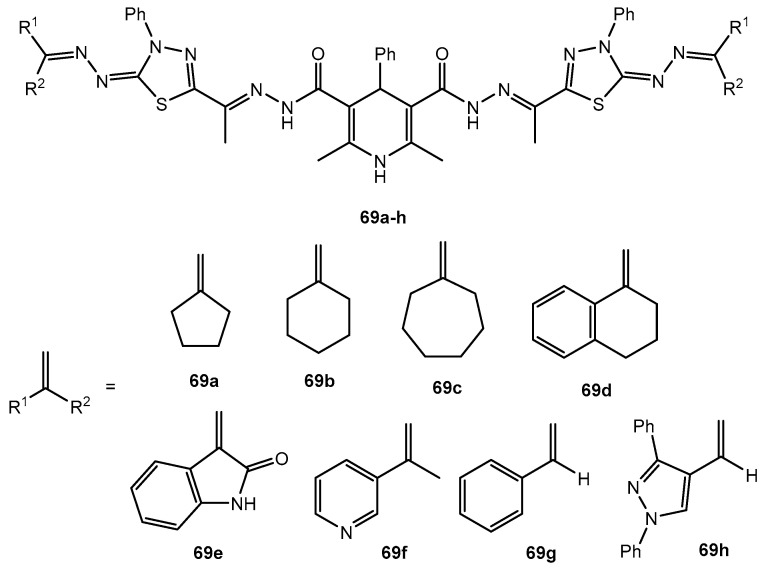
The structure of 1,4-dihydropyridine hybrids with 1,3,4-thiadiazole.

Gomha et al. obtained a series of 1,4-dihydropyridine hybrids with 1,3,4-thiadiazole (**69a**–**h**, Figure 56) [95]. The compounds were assayed in vitro for their antibacterial activity against Gram-positive bacteria (*Staphylococcus aureus*, *Staphylococcus epidermidis*, *Bacillus subtilis*, *Streptococcus pyogenes*) and Gram-negative bacteria (*Pseudomonas aeruginosa*, *Escherichia coli*, *Klebsiella pneumoniae*, *Salmonella typhimurium*) at a concentration of 30 μg/mL; Gentamicin and Ampicillin were used as reference drugs (Table 35). Derivative **69e**, bearing a 2-oxoindolin-3-ylidene moiety, indicated higher inhibitory activity against all of the examined bacteria than the reference standards.

**Figure 57:**
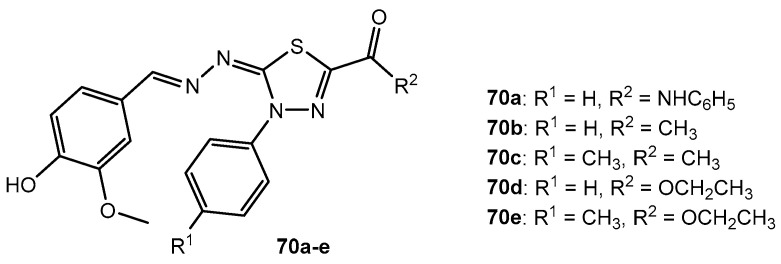
The structure of the obtained tri-substituted 1,3,4-thiadiazole derivatives.

Rashdan’s group synthesized a series of tri-substituted 1,3,4-thiadiazole derivatives (**70a**–**e**, Figure 57) [96]. The compounds were evaluated for antimicrobial activity against *Escherichia coli*, *Pseudomonas aeruginosa*, *Proteus vulgaris*, *Bacillus subtilis*, and *Staphylococcus aureus* (Table 36). Compound **70a**, containing phenylamino group at the R^2^ site, demonstrated notable broad-spectrum efficacy across all tested strains, with low effective concentrations ranging from 20 to 40 µg/mL.

**Figure 58:**
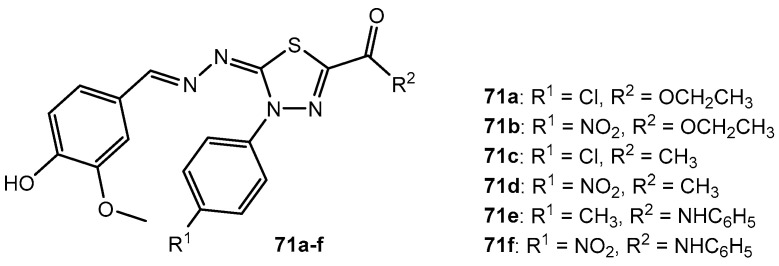
The structure of tri-substituted 1,3,4-thiadiazole derivatives.

The same research group also obtained another series of 1,3,4-thiadiazole derivatives (**71a**–**f**, Figure 58) [97]. These compounds were tested against four pathogenic bacteria: *Klebsiella pneumoniae*, *Pseudomonas aeruginosa*, *Staphylococcus aureus*, and *Bacillus subtilis* (Table 37). Compound **71f**, containing a nitro group at the R^1^ site and a phenylamino group at the R^2^ site, exhibited the strongest antibacterial activity against all evaluated strains.

### 2.2. Antifungal Activity

#### 2.2.1. Disubstituted 1,3,4-Thiadiazole Derivatives

As previously noted, Mao et al. synthesized a series of derivatives containing a 1,3,4-thiadiazole thione moiety (**6a**–**p**, Figure 7) [46]. In addition to antibacterial evaluation, the compounds were assessed for antifungal activity against *Sclerotinia sclerotiorum*, *Rhizoctonia solani*, *Magnaporthe oryzae*, and *Colletotrichum gloeosporioides* at a concentration of 50 μg/mL (Table 38). Compound **6b**, containing a fluoro substituent at the *ortho* position of the benzene ring, demonstrated excellent efficacy against *S. sclerotiorum* (EC_50_ = 0.51 µg/mL), comparable to that of the commercial fungicide Carbendazim (EC_50_ = 0.57 µg/mL). The authors concluded that derivatives containing electron-withdrawing substituents at the *ortho* position exhibited enhanced antifungal activity (e.g., **6b**, R = 2-F), whereas *meta*-substituted derivatives showed primarily antibacterial potency (e.g., **6k**, R = 3-OCF_3_).

Hafidh et al. investigated hybrid silica gels incorporating 1,3,4-thiadiazole rings (**7a**, **7b**, Figure 8) for their antimicrobial potential, including antifungal activity against *Candida albicans* [47]. The MIC values were 0.25 mg/mL for compound **7a**, containing a (5-amino-1,3,4-thiadiazol-2-yl)amino moiety, and 0.5 mg/mL for **7b**, containing a (5-mercapto-1,3,4-thiadiazol-2-yl)thio moiety, indicating relatively low antifungal efficacy in comparison with the reference agent Gentamicin (MIC = 7.81 µg/mL).

**Figure 59:**
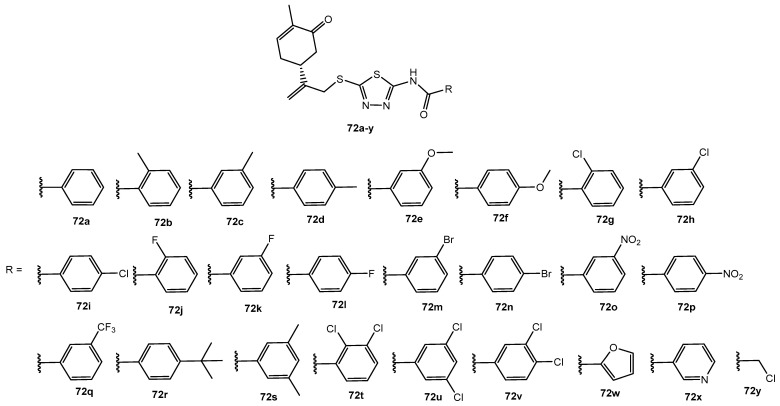
The structure of *L*-carvone-based 1,3,4-thiadiazole-amide derivatives.

Baoyu Li et al. synthesized a series of derivatives incorporating a 1,3,4-thiadiazole ring (**72a**–**y**, Figure 59) [98]. The compounds were evaluated for antifungal activity against *Physalospora piricola*, *Colletotrichum orbiculare*, *Cercospora arachidicola*, *Gibberella zeae*, *Alternaria solani*, *Rhizoctonia solani*, *Fusarium oxysporum*, and *Bipolaris maydis* at a concentration of 50 μg/mL (Table 39). Compound **72b**, containing a 2-methylphenyl group in the 1,3,4-thiadiazole-amide fragment, showed broad-spectrum activity with high inhibition rates ranging from 67% to 89% across all tested phytopathogens. Among the tested 1,3,4-thiadiazoles, compound **72d**, with a 4-methylphenyl substituent in the 1,3,4-thiadiazole-amide fragment, demonstrated particularly strong activity against *Cercospora arachidicola*, *Gibberella zeae*, and *Alternaria solani*, with inhibition rates of 85%, 83%, and 77%, respectively, all superior or comparable to those of the reference fungicide Boscalid. In the search for correlations between the structure of the synthesized compounds and their biological activity, the authors found that small groups at the *ortho* position of the terminal benzene ring (e.g., CH_3_ or F) enhanced activity. The same trend was observed for bulky (e.g., *tert*-butyl) and electron-donating groups (e.g., OCH_3_) at the *para* position. In contrast, halogens or electron-withdrawing groups (e.g., NO_2_) generally reduced antifungal activity.

A series of 1,3,4-thiadiazole derivatives synthesized by Mehta and coworkers (**8a**–**o**, Figure 9 [48], initially tested for antibacterial properties, was also evaluated for antifungal activity against *Candida albicans*, *Aspergillus niger*, and *Aspergillus clavatus*. Table 40 presents the minimum inhibitory concentration (MIC) values of the tested compounds. The strongest activity against *Candida albicans* was observed for compounds **8c** (R = 4-Br), **8e** (R = 4-Cl), **8f** (R = 2-F), **8i** (R = 2-OH), and **8l** (R = 4-OCH_3_), with MIC values of 250, 100, 250, 250, and 250 μg/mL, respectively. The two previously mentioned derivatives, **8c** and **8i**, as well as compounds **8j** (R = 4-OH) and **8o** (R = 4-NO_2_), showed activity against *Aspergillus niger* with MIC values of 100 μg/mL, while compound **8e** inhibited *Aspergillus clavatus* with an MIC of 100 μg/mL.

The previously discussed series of 1,3,4-thiadiazoles (**9a**–**d**, **10**, Figure 10), synthesized by Danilova’s group [49], was also tested for antifungal activity against *Fusarium oxysporum*, *Alternaria alternata*, and *Bipolaris sorokiniana*. Among the tested compounds, only 5,5’-methylenebis(1,3,4-thiadiazol-2-amine) (**9a**) exhibited activity, showing inhibition against *Alternaria alternata* at a concentration of 200 μg/mL.

**Figure 60:**
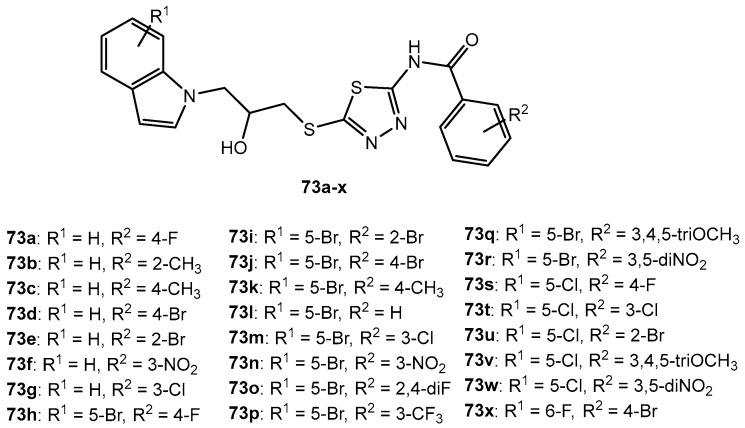
The structure of indole derivatives containing 1,3,4-thiadiazole.

He et al. synthesized a series of indole derivatives incorporating a 1,3,4-thiadiazole ring (**73a**–**x**, Figure 60) [99]. Selected compounds were evaluated for antifungal activity against *Botrytis cinerea*, *Tomato Botrytis cinerea*, and *Phomopsis* sp., with EC_50_ values presented in Table 41. Among them, compound **73b**, containing a 2-methylphenyl group in the amide fragment, exhibited the highest level of activity against *Botrytis cinerea*, with an EC_50_ of 2.7 μg/mL, surpassing the efficacy of the reference fungicide Azoxystrobin (EC_50_ = 14.5 μg/mL). The authors observed that compounds unsubstituted at the indole fragment (**73a**–**g**, R^1^ = H) generally exhibited stronger inhibition than those bearing halogen substituents (**73h**–**x**; R^1^ = Br, Cl, F). The presence of electron-donating R^2^ substituents on the benzene ring of the opposite amide group improved antifungal activity compared with electron-withdrawing substituents. Furthermore, it was noted that the parent indole structure displayed higher activity when the benzamide fragment carried an electron-donating substituent, particularly when R^2^ = CH_3_.

**Figure 61:**
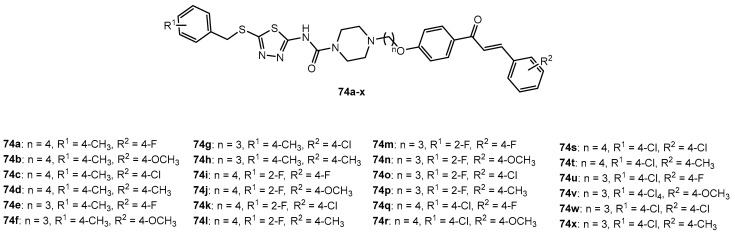
The structure of chalcone derivatives containing 1,3,4-thiadiazole.

Xue and coworkers synthesized a series of chalcone derivatives containing a 1,3,4-thiadiazole moiety (**74a**–**x**, Figure 61) [100]. The compounds were tested against *Rhizoctonia solani*, *Phomopsis* sp., and *Phytophthora capsici*. Inhibition rates at a concentration of 100 μg/mL are summarized in Table 42. Bioactivity screening revealed that several derivatives exhibited notable antifungal activity with high levels of inhibition. Further evaluation showed that compound **74d**, bearing 4-methylphenyl substituents at opposite terminal positions, had an EC_50_ value of 14.4 μg/mL against *Phomopsis* sp., significantly outperforming the reference fungicides Azoxystrobin (32.2 μg/mL) and Fluopyram (54.2 μg/mL). In general, it was found that compounds with electron-donating groups at the R^2^ or R^1^ positions exhibited stronger activity than those with electron-withdrawing groups. It was also observed that derivatives containing an alkyl linker composed of four methylene groups (n = 4) were more active than their counterparts with three methylene groups (n = 3).

The previously described series of 1,3,4-thiadiazole-containing Schiff bases synthesized by Zahoor and his team (**11a**–**l**, Figure 11) [50] was also evaluated for antifungal activity against *Alternaria alternata*. Inhibition rates are summarized in Table 43. Compounds **11a** (R = 2-NO_2_-4-CF_3_C_6_H_3_), **11d** (R = 4-Cl-3-OHC_6_H_3_), and **11i** (R = 3,5-diFC_6_H_3_) showed notable antifungal efficacy, with inhibition levels of 43.4%, 31.9%, and 34.3%, respectively, compared to the reference drug Terinafine (50.7%).

**Figure 62:**
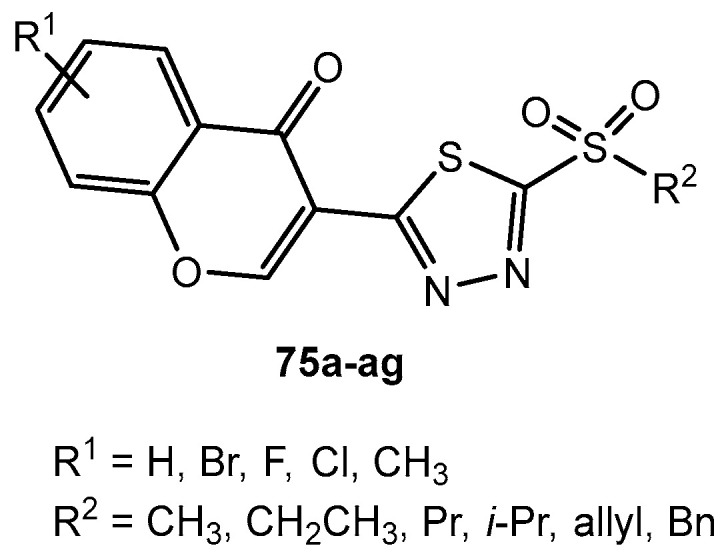
The structure of 5-sulfonyl-1,3,4-thiadiazole flavonoids.

Dai et al. synthesized a series of disubstituted 1,3,4-thiadiazole derivatives (**75a**–**ag**, Figure 62) [101]. The compounds were evaluated for antifungal activity against *Botrytis cinerea*, *Alternaria solani*, *Rhizoctonia solani*, *Fusarium graminearum*, and *Colletotrichum orbiculare*. In vitro inhibition rates at a concentration of 10 μg/mL are presented in Table 44. Most flavonoid-based derivatives exhibited excellent broad-spectrum antifungal activity. Notably, the EC_50_ values of several compounds against *Rhizoctonia solani* were below 4 μg/mL. Compounds **75m**, with bromine at the 6 position of the chromone fragment and a propyl group in the sulfonamide part (EC_50_ = 0.49 μg/mL), **75o**, with chlorine at the 6 position of the chromone fragment and a propyl group in the sulfonamide part (EC_50_ = 0.37 μg/mL), and **75s**, with bromine at the 6 position of the chromone fragment and an isopropyl group in the sulfonamide part (EC_50_ = 0.37 μg/mL), displayed the highest potency, surpassing that of the reference fungicide Carbendazim (EC_50_ = 0.52 μg/mL). Structure–activity relationship analysis revealed that electron-withdrawing groups, such as bromine or chlorine, at the 6 position of the chromone fragment improved activity compared with methyl groups. For substituents located on the sulfone moiety, it was observed that large or branched groups also enhanced antifungal activity. The length of the alkyl chain (n) in the sulfonamide part was also important; longer chains (n = 4) in brominated derivatives improved activity compared with shorter counterparts (n = 3).

A series of 2,5-disubstituted 1,3,4-thiadiazole derivatives synthesized by Li and coworkers (**12a**–**r**, Figure 12) [51] was also evaluated for antifungal activity against *Colletotrichum gloeosporioides*, *Nakazawaea ishiwadae*, *Gilbertella persicaria*, and *Fusarium* sp. Inhibition rates at a concentration of 100 μg/mL are summarized in Table 45. Compound **12b**, containing a methyl group at the R^1^ site and an ethyl group at the R^2^ site, exhibited remarkable activity against *Gilbertella persicaria*, with an EC_50_ value of 6.71 μg/mL, significantly exceeding that of the reference fungicide Prochloraz (EC_50_ = 22.03 μg/mL).

**Figure 63:**
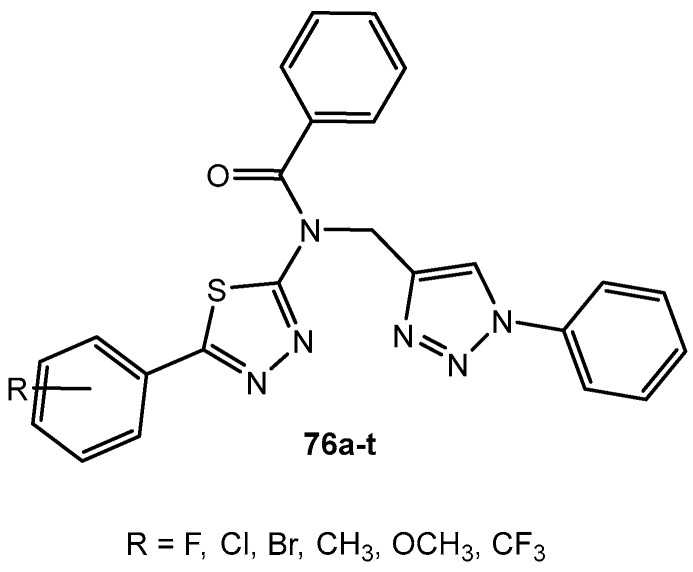
The structure of 1,3,4-thiadiazol-2-yl-benzamide derivatives.

Fu’s group synthesized a series of substituted 5-aryl-2-amino-1,3,4-thiadiazoles bearing a benzamide moiety (**76a**–**t**, Figure 63) [102]. The compounds were tested for antifungal activity against *Rhizoctonia solani*, *Botrytis cinerea*, *Stemphylium lycopersici*, *Curvularia lunata*, and *Pythium aphanidermatum* (Table 46). Most derivatives demonstrated excellent in vitro fungicidal efficacy. Notably, compound **76p**, containing a 4-trifluoromethylphenyl substituent, exhibited the highest activity against *Rhizoctonia solani* (EC_50_ = 0.0028 μmol/L), **76l**, with a 2-methoxyphenyl group, was most effective against *Botrytis cinerea* (EC_50_ = 0.0024 μmol/L), and **76e**, bearing a 3-chlorophenyl substituent, showed potent activity against both *Stemphylium lycopersici* and *Curvularia lunata* (EC_50_ = 0.0105 μmol/L and 0.005 μmol/L, respectively). The authors made general observations regarding the influence of structural features of the synthesized compounds on their antifungal activity against specific strains. As shown in the results, the order of activity against *Rhizoctonia solani* was as follows: 3-CF_3_ > 4-OCH_3_ > 4-CH_3_ > 2,6-diF > 4-CF_3_ > 2-F. In the case of *Botrytis cinerea*, it was observed that only the presence of methoxy OCH_3_ and bromine Br substituents exerted a notable antifungal effect. Against *Stemphylium lycopersici*, only the 4-chloro-substituted compound displayed the same activity as the positive control drug.

**Figure 64:**
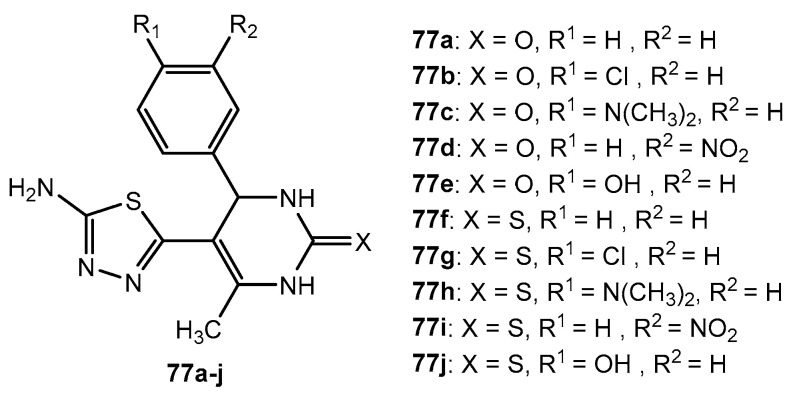
The structure of 1,3,4-thiadiazole-2-yl-pyrimidine derivatives.

Durairaj’s group synthesized a series of 1,3,4-thiadiazole derivatives directly linked to a pyrimidine scaffold (**77a**–**j**, Figure 64) [103]. The compounds were evaluated for antifungal activity against *Aspergillus niger*, *Penicillium* species, and *Candida albicans*. As shown in Table 47, 5-(1,3,4-thiadiazol-2-yl)-3,4-dihydropyrimidin-2(1*H*)-one containing a dimethylamino group at the R^1^ site (**77c**) and 5-(1,3,4-thiadiazol-2-yl)-3,4-dihydropyrimidine-2(1*H*)-thione containing chlorine at the R^1^ site (**77g**) demonstrated promising activity against all three fungal strains.

**Figure 65:**
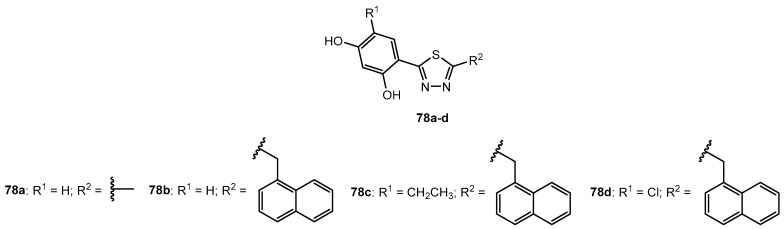
Derivatives of 1,3,4-thiadiazole containing resorcinol moiety.

Dróżdż et al. evaluated four 1,3,4-thiadiazole derivatives (**78a**–**d**, Figure 65) [104] for antifungal activity against *Candida albicans* and *Candida parapsilosis*. The determined minimum inhibitory concentrations (MIC) are summarized in Table 48. For compounds **78a**, containing a methyl group at the R^2^ site, and **78d**, containing chlorine at the R^1^ site and a naphthalen-1-ylmethyl group at the R^2^ site, the MIC_100_ values—defined as the minimum concentration required to completely inhibit fungal growth—ranged from 64 to 128 µg/mL, depending on the strain. As a further aspect of the study, selected thiadiazole derivatives were tested in combination with Amphotericin B, revealing strong synergistic antifungal interactions.

Panwar’s group synthesized a series of [2-phenyl-1-(*p*-tolyl)pyrido[3,2-*f*]quinazolin-4(1*H*)-yl]-1,3,4-thiadiazolyl]-4-piperazine derivatives (**13a**–**i**, Figure 13) [52]. In addition to antibacterial assessment, the compounds were tested for antifungal activity against *Aspergillus fumigatus*, *Candida albicans*, and *Candida krusei*. Table 49 presents the zones of inhibition recorded at a concentration of 250 μg/mL. Several derivatives exhibited antifungal efficacy against *Aspergillus fumigatus*, a strain intrinsically resistant to Fluconazole.

As previously mentioned, Blaja et al. synthesized tetranorlabdane derivatives bearing 1,3,4-thiadiazole units (**14a**–**c**, Figure 14) [53]. The compounds were evaluated for antifungal activity against five fungal strains: *Alternaria alternata*, *Aspergillus niger*, *Penicillium chrysogenum*, *Penicillium frequentans*, and *Fusarium solani*. The results, summarized in Table 50, indicate that only 5-(((8*aS*)-2,5,5,8*a*-tetramethyl-4*a*,5,6,7,8,8*a*-hexahydronaphthalen-1-yl)methyl)-1,3,4-thiadiazol-2-amine (**14a**) exhibited pronounced antifungal activity, with an MIC value of 0.125 μg/mL.

**Figure 66:**
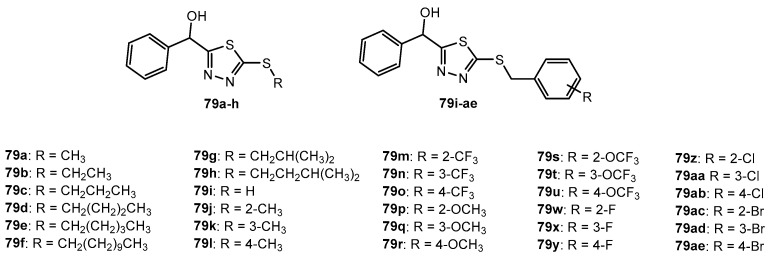
The structure of 1,3,4-thiadiazole thioether derivatives.

Shi et al. synthesized a series of phenylmethanol-linked 1,3,4-thiadiazole thioethers (**79a**–**ad**, Figure 66) [105]. The compounds were evaluated for antifungal activity against *Alternaria solani*, *Gibberella saubinetii*, *Verticillium dahliae*, *Gibberella zeae*, and *Thanatephorus cucumeris* (Table 51). Bioassay results revealed that compound **79j** exhibited excellent efficacy against *Thanatephorus cucumeris*, with an EC_50_ value of 9.7 μg/mL. Further studies demonstrated that (5-((2-methylbenzyl)thio)-1,3,4-thiadiazol-2-yl)(phenyl)methanol (**79j**) not only significantly inhibited *Thanatephorus cucumeris* mycelial growth but also suppressed sclerotia formation and exhibited substantial in vivo protective (61.1%) and curative (67.9%) effects at 200 μg/mL.

The modified chitosan–thiadiazole conjugate developed by Ibrahim et al. (**15**, Figure 15) [54], previously tested for antibacterial properties, was also evaluated for antifungal activity against *Candida albicans*. At a concentration of 50 μg/mL, the compound exhibited a zone of inhibition measuring 8 mm, indicating moderate antifungal potential.

**Figure 67:**
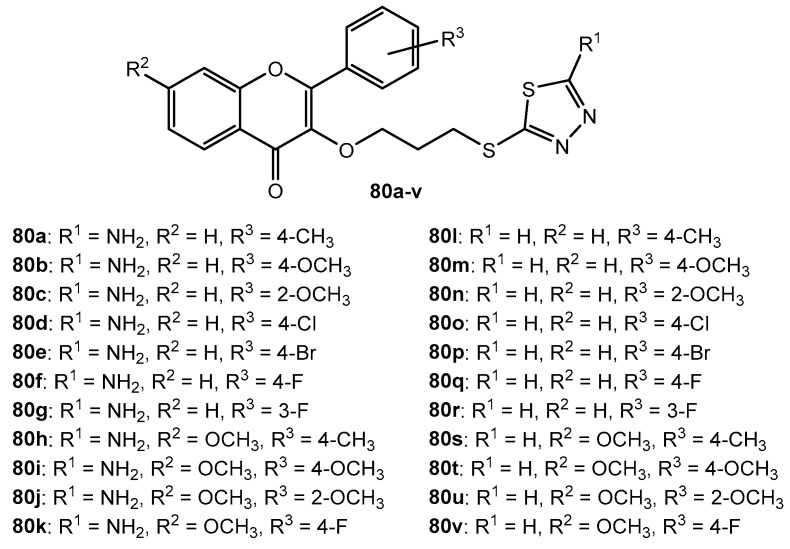
The structure of flavanol derivatives containing 1,3,4-thiadiazole scaffold.

Zhou et al. synthesized a series of flavanol derivatives containing a 1,3,4-thiadiazole moiety (**80a**–**v**, Figure 67) [106]. The compounds were screened for antifungal activity against *Rhizoctonia solani*, *Botrytis cinerea*, *Fusarium graminearum*, *Colletotrichum gloeosporioides*, *Sclerotinia sclerotiorum*, *Phytophthora capsici*, *Alternaria brassicae*, *Fusarium oxysporum f*. sp. *cucumerinum*, *Fusarium oxysporum f*. sp. *capsicum*, and *Phomopsis* sp. Inhibition rates obtained from biological assays are summarized in Table 52. Several derivatives demonstrated excellent antifungal efficacy. Notably, compounds **80l**, **80m**, **80q**, and **80r**, bearing 4-methylphenyl, 4-methoxyphenyl, 4-fluorophenyl, and 3-fluorophenyl substituents at the flavanol core, showed inhibition rates against *Botrytis cinerea* of 91.7%, 83.2%, 87.3%, and 96.2%, respectively, all exceeding the activity of the reference fungicide Azoxystrobin (80.7%). Additionally, compounds **80n** and **80u**, characterized by the presence of one or two methoxy groups, displayed superior activity against *Phomopsis* sp., with inhibition rates of 73.8% and 72.8% compared to 58.1% for Azoxystrobin. In summary, antifungal activity was higher for derivatives unsubstituted at the 5 position of the 1,3,4-thiadiazole ring (R^1^ = H) than for those bearing an amino group (R^1^ = NH_2_). Compounds with electron-withdrawing groups on the phenyl ring of the flavanol scaffold displayed stronger fungicidal effects, with an order of efficacy of **80r** (R^3^ = 3-F) > **80l** (R^3^ = 4-CH_3_) > **80m** (R^3^ = 4-OCH_3_). Furthermore, substitution at R^3^ with 3-F conferred a greater inhibitory effect than substitution with 4-F. Overall, compound **80r** (R^1^ = H, R^2^ = H, R^3^ = 3-F) demonstrated markedly superior performance relative to the other target compounds and Azoxystrobin.

Thanh et al. synthesized a series of 1,3,4-thiadiazole derivatives incorporating a thiourea scaffold (**16a**–**i**, Figure 16) [55]. In addition to antibacterial testing, the compounds were evaluated for antifungal activity against *Aspergillus niger*, *Aspergillus flavus*, *Candida albicans*, *Saccharomyces cerevisiae*, and *Fusarium oxysporum* (Table 53). Compounds **16b**, bearing a methyl substituent at the R site, and **16c**, with the ethyl substituent, selectively inhibited the growth of *Candida albicans*, exhibiting strong activity with MIC values of 0.78 and 1.56 μg/mL, respectively. More broadly, compound **16i**, bearing an isopentyl substituent at the R site, showed potent activity against *Aspergillus niger*, *Saccharomyces cerevisiae*, and *Fusarium oxysporum*, with MIC values of 0.78 μg/mL, superior to those of the reference drugs Miconazole and Fluconazole. The authors concluded that the presence of long chains (**16i**) at the R site and branching (**16g**, **16i**) significantly enhanced antifungal activity compared to the corresponding unbranched analogues (**16f**, **16h**).

The previously discussed series of thiadiazole azetidin-2-one derivatives synthesized by Kumar et al. (**20a**–**g**, Figure 19, Table 54) [58] was also evaluated for antifungal activity against *Trichoderma harzianum* and *Aspergillus niger*. Additional testing revealed that compound **20g**, containing bromine at the R^1^ site and a 4-chlorine substituent at the R^2^ site, exhibited remarkable efficacy against *Aspergillus niger*, with an MIC value of 3.42 µM, while derivative **20f**, containing chlorine at the R^1^ site and a 4-nitro group at the R^2^ site, was the most active against *Trichoderma harzianum*, outperforming the reference drug Fluconazole (MIC = 3.71 µM).

As previously mentioned, Alqahtani et al. synthesized a series of compounds bearing a benzothiazolotriazole scaffold linked to a 1,3,4-thiadiazole ring (**24a**–**c**, Figure 23) [62]. In addition to antibacterial evaluation, the compounds were tested for antifungal activity against *Candida albicans*, *Aspergillus fumigatus*, and *Penicillium chrysogenum* (Table 55). Among them, compound **24b**, bearing 4-bromophenylamino moiety on the 1,3,4-thiadiazole ring, exhibited the highest potency, with MIC values of 8 μg/mL against *Candida albicans* and *Penicillium chrysogenum* and 16 μg/mL against *Aspergillus fumigatus*.

**Figure 68:**
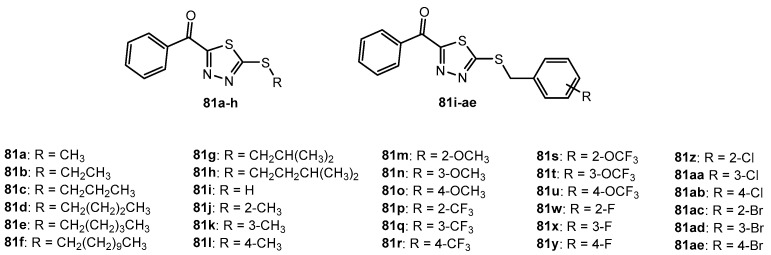
The structure of 1,3,4-thiadiazole-2-thioethers.

Dou et al. synthesized a series of acetophenone derivatives containing 1,3,4-thiadiazole-2-thioether moieties (**81a**–**ae**, Figure 68) [107]. The compounds were evaluated for antifungal activity against *Gibberella saubinetii*, *Verticillium dahliae*, *Alternaria solani*, *Gibberella zeae*, and *Thanatephorus cucumeris* (Table 56). Preliminary bioassay results indicated that compounds **81a**–**c** exhibited inhibitory effects against all five tested fungal strains. Notably, the EC_50_ value of (5-(ethylthio)-1,3,4-thiadiazol-2-yl)(phenyl)methanone (**81b**) against *Thanatephorus cucumeris* was 22.2 μg/mL, while **81c** showed an EC_50_ of 21.5 μg/mL against *Gibberella saubinetii*.

Pham and coworkers synthesized a series of 5-substituted-2-amino-1,3,4-thiadiazole derivatives (**25a**–**l**, Figure 24) [63]. In addition to the previously discussed antibacterial evaluation, the compounds were tested for antifungal activity against *Candida albicans* and *Aspergillus niger* (Table 57). Compound **25g**, bearing a 3,4-dichlorophenyl group on the 1,3,4-thiadiazole ring, exhibited the highest potency among the synthesized derivatives, with MIC values of 16 μg/mL against *Candida albicans* and 64 μg/mL against *Aspergillus niger*, outperforming Fluconazole in both cases.

**Figure 69:**
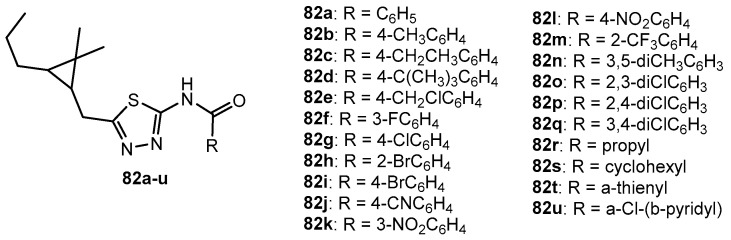
The structure of 1,3,4-thiadiazole-amide derivatives.

Zou et al. synthesized a series of 1,3,4-thiadiazole-amide derivatives incorporating a gem-dimethyl-cyclopropane ring (**82a**–**u**, Figure 69) [108]. The compounds were evaluated for antifungal activity against *Fusarium oxysporum f*. sp. *cucumerinum*, *Cercospora arachidicola*, *Physalospora piricola*, *Alternaria solani*, *Gibberella zeae*, *Rhizoctonia solani*, *Bipolaris maydis*, and *Colletotrichum orbiculare* (Table 58). Preliminary bioassay results indicated that compound **82i**, containing a 4-bromophenyl substituent at the amide moiety, exhibited broad-spectrum antifungal activity against the tested strains.

Sunitha et al. synthesized a series of azo-imine thiadiazole derivatives (**28a**–**e**, Figure 27) [66] and evaluated their antifungal activity against *Aspergillus niger*, *Aspergillus flavus*, and *Rhizopus stolonifera* using Fluconazole as the reference drug (Table 59). Overall, compound **28c**, containing a 3-hydroxy-4-methoxyphenyl substituent at the imine fragment, demonstrated moderate inhibitory activity against the tested fungal strains.

**Figure 70:**
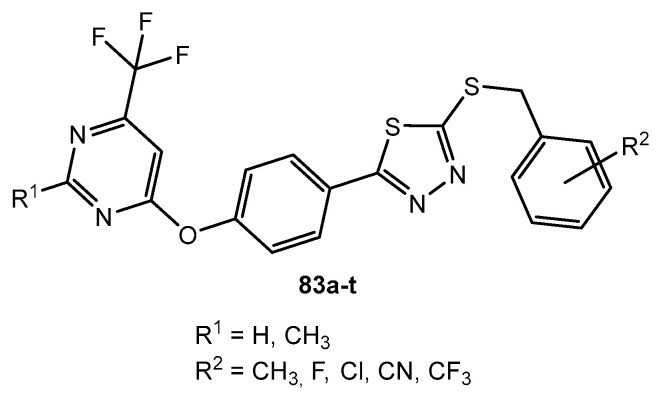
The structure of pyrimidine-1,3,4-thiadiazole derivatives.

Pan et al. synthesized a series of pyrimidine derivatives bearing a 1,3,4-thiadiazole core (**83a**–**t**, Figure 70) [109]. The compounds were tested for antifungal activity against *Botrytis cinerea*, *Botryosphaeria dothidea*, and *Phomopsis* sp. at a concentration of 50 μg/mL (Table 60). Biological assay results demonstrated that compound **83h** containing (4-fluoromethylbenzyl)thio moiety exhibited lower EC_50_ values (25.9 and 50.8 μg/mL) against *Phomopsis* sp. compared to the reference fungicide Pyrimethanil (32.1 and 62.8 μg/mL). Further structure–activity relationship analysis showed that more than 80% of the tested compounds exhibited excellent antifungal activity against *Phomopsis* sp. and *Botrytis cinerea*. Modification of the R^1^ substituent in the pyrimidine ring (H or CH_3_) did not significantly enhance antifungal potency; however, for *Phomopsis* sp., the number of compounds with R^1^ = H and activity above 80% was twice that observed for compounds with R^1^ = CH_3_. Moreover, introducing strong electron-withdrawing groups at R^2^ (e.g., CN or CF_3_) within the benzylthio fragment enhanced activity, whereas introduction of an alkyl group (CH_3_) had little effect.

Yu et al. synthesized a series of thiochroman-4-one derivatives incorporating carboxamide and 1,3,4-thiadiazole thioether moieties (**29a**–**o**, Figure 28) [67], previously reported for their antibacterial activity. The compounds were also evaluated for antifungal activity against *Botrytis cinerea*, *Verticillium dahliae*, and *Fusarium oxysporum* at a concentration of 50 μg/mL (Table 61). Notably, compound **29m**, containing a propyl group at the R^1^ site and a methyl group at the R^2^ site, exhibited superior activity against *Botrytis cinerea* compared to the reference fungicide Carbendazim.

As previously mentioned, Shu et al. synthesized a series of galactoside derivatives containing a 1,3,4-thiadiazole moiety (**30a**–**t**, Figure 29) [68]. The compounds were tested for antifungal activity against *Gibberella zeae*, *Botryosphaeria dothidea*, *Phytophthora infestans*, *Phomopsis* sp., and *Thanatephorus cucumeris* at a concentration of 50 μg/mL (Table 62). Among them, compound **30p**, containing a nitro substituent at the *meta* position, compound **30r**, containing a nitro substituent at the *para* position, and compound **30t**, with a trifluoromethyl group at the *meta* position, demonstrated satisfactory in vitro activity against *Phytophthora infestans*, with inhibition rates of 80.1%, 79.7%, and 79.3%, respectively. These results were comparable to the activity of the reference fungicide Dimethomorph (78.2%).

**Figure 71:**
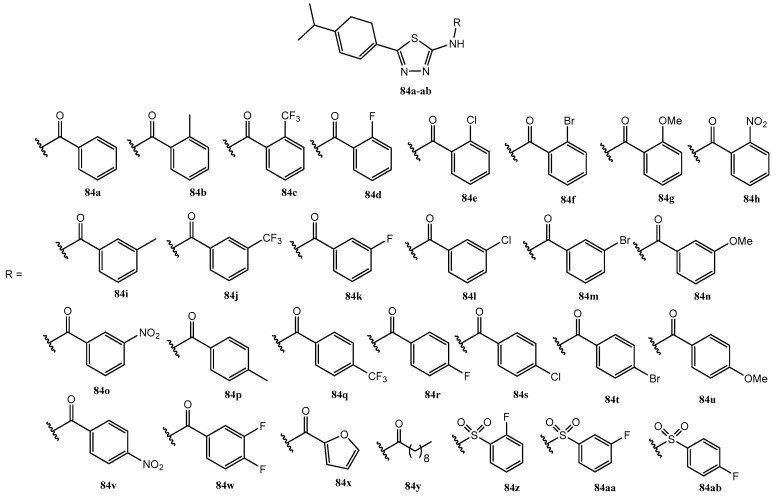
The structure of terpene-derived 1,3,4-thiadiazole derivatives.

Geng et al. synthesized a series of terpene-derived compounds incorporating a 1,3,4-thiadiazole moiety (**84a**–**ab**, Figure 71) [110]. The compounds were tested for antifungal activity against *Valsa mali* (Table 63). The results indicated that several derivatives exhibited satisfactory inhibitory effects, with some outperforming the commercial fungicide Boscalid. The most potent compound, **84aa**, containing a (3-fluorophenyl)sulfonyl group, demonstrated a favorable EC_50_ value of 3.785 μg/mL.

Prasad et al. synthesized a series of quinoline-bridged thiophene derivatives linked to a 1,3,4-thiadiazole ring (**31a**–**e**, Figure 30) [69]. In addition to antibacterial evaluation, the compounds were tested for antifungal activity against *Aspergillus niger* and *Aspergillus flavus* at a concentration of 10 μg/mL (Table 64). The study revealed that among the tested derivatives, only compound **31d**, bearing a nitro group at the R site, exhibited notable activity against *Aspergillus niger*.

Acar Çevik et al. synthesized a series of benzimidazole derivatives containing a 1,3,4-thiadiazole scaffold (**32a**–**k**, Figure 31) [70]. The antifungal activity of all compounds was assessed by determining their minimum inhibitory concentrations (MIC) against *Candida albicans*, *Candida krusei*, *Candida glabrata*, and *Candida parapsilosis* (Table 65). Screening results revealed that compound **32a**, containing a methyl group at the R site, and compound **32h**, containing an isopropyl group, exhibited the highest potency against *Candida albicans*, with MIC values of 1.95 μg/mL—twice as effective as the reference drug Voriconazole (3.90 μg/mL) and four times more effective than Fluconazole (7.81 μg/mL). The authors concluded that the enhanced antifungal activity was attributed to the presence of alkylamino groups (R) at the 5 position of the 1,3,4-thiadiazole ring, with small to medium alkyl chains (methyl, isopropyl) providing optimal activity.

**Figure 72:**
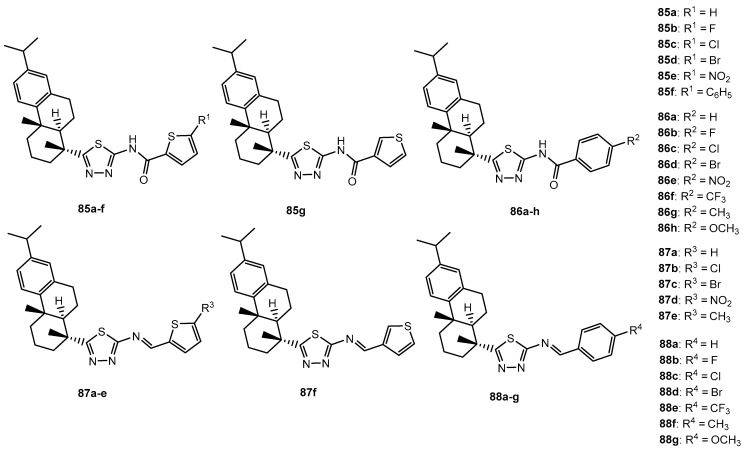
The structure of dehydroabietyl-1,3,4-thiadiazole-2-amide and dehydroabietyl-1,3,4-thiadiazole-2-imine derivatives.

Mao et al. synthesized four series of dehydroabietyl-1,3,4-thiadiazole-2-amide and dehydroabietyl-1,3,4-thiadiazole-2-imine derivatives (**85a**–**g**, **86a**–**h**, **87a**–**f**, **88a**–**g**, Figure 72) [111]. The antifungal activity of all synthesized compounds was evaluated against *Sclerotinia sclerotiorum*, *Botrytis cinerea*, *Magnaporthe oryzae*, and *Fusarium oxysporum*. At a concentration of 100 mg/L, the mycelial growth inhibition rates against *Sclerotinia sclerotiorum*, *Botrytis cinerea*, and *Magnaporthe oryzae* were below 20%, indicating weak activity. However, the activity against *Fusarium oxysporum* ranged from moderate to significant. Notably, compound **85e**, substituted with a nitro group on the thiophene ring, demonstrated excellent antifungal efficacy, with an EC_50_ value of 0.618 mg/L—lower than that of the reference fungicide Carbendazim (0.649 mg/L). In vivo studies further confirmed that **85e** provided a protective effect on cucumber plants.

As previously mentioned, Garg and coworkers synthesized a series of 1,3,4-thiadiazole derivatives bearing 2,3-disubstituted thiazolidinone moieties (**35a**–**j**, Figure 34) [73]. In addition to antibacterial evaluation, the compounds were tested for antifungal activity against *Candida albicans* and *Aspergillus niger* (Table 66). The most promising result was obtained for compound **35b**, bearing a dimethylamino substituent, which exhibited antifungal activity slightly superior to or comparable with that of the standard drug Miconazole.

**Figure 73:**
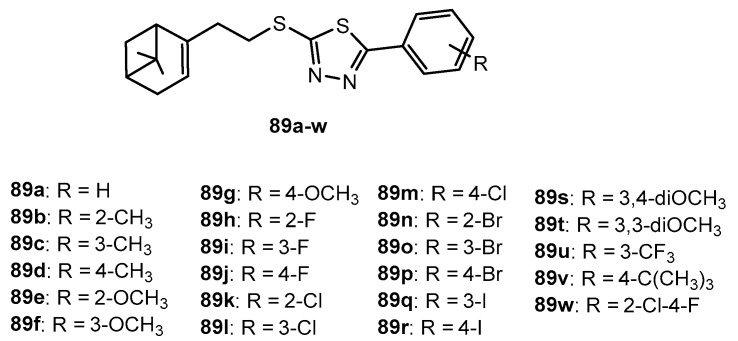
The structure of 1,3,4-thiadiazole-thioether compounds.

Wang et al. synthesized a series of novel nopol derivatives incorporating a 1,3,4-thiadiazole–thioether moiety (**89a**–**w**, Figure 73) [112]. The antifungal activity of all compounds was evaluated against *Fusarium oxysporum f*. sp. *cucumerinum*, *Cercospora arachidicola*, *Physalospora piricola*, *Alternaria solani*, *Gibberella zeae*, *Rhizoctonia solani*, *Bipolaris maydis*, and *Colletotrichum orbiculare* at a concentration of 50 μg/mL (Table 67). Compounds **89i** (R = 3-F), **89f** (R = 3-OCH_3_), and **89q** (R = 3-I) exhibited excellent inhibition rates of 88.9%, 77.8%, and 77.8%, respectively, against *Physalospora piricola*, surpassing the reference fungicide Chlorothalonil (75%). Additionally, compound **89m** (R = 4-Cl) showed strong activity against *Rhizoeotnia solani*, with an inhibition rate of 80.7%.

**Figure 74:**
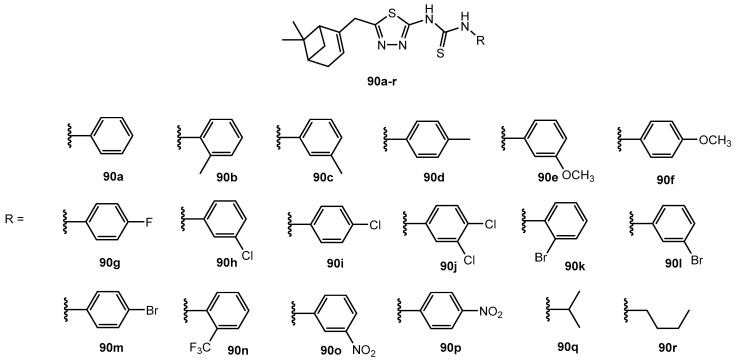
The structure of nopol-derived 1,3,4-thiadiazole–thiourea compounds.

Chen et al. synthesized a series of 1,3,4-thiadiazole–thiourea derivatives (**90a**–**r**, Figure 74) [113]. All compounds were evaluated for antifungal activity (Table 68). Several target derivatives demonstrated superior efficacy against *Physalospora piricola*, *Cercospora arachidicola*, and *Alternaria solani* compared to the commercial fungicide Chlorothalonil at a concentration of 50 μg/mL. Notably, compound **90c**, bearing a 3-methylphenyl substituent on the thiourea fragment, compound **90q**, with an isopropyl substituent, and compound **90i**, with a 4-chlorophenyl substituent, exhibited inhibition rates of 86.1%, 86.1%, and 80.2%, respectively, against *Physalospora piricola*, clearly outperforming the reference control.

**Figure 75:**
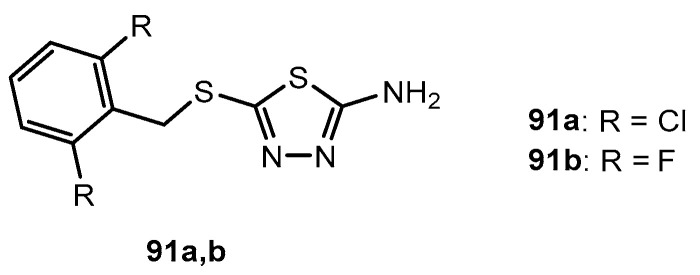
The structure of 2-amino-1,3,4-thiadiazole derivatives.

The Tahtacı group synthesized two 1,3,4-thiadiazole derivatives containing halogens (**91a**, **91b**, Figure 75) as intermediates in the development of their imidazothiadiazole analogues [114]. In vitro antifungal activity was evaluated by determining the minimum inhibitory concentration (MIC), minimum fungicidal concentration (MFC), and lethal dose (LD_50_) values against *Alternaria solani*, *Fusarium oxysporum f*. sp. *melonis*, and *Verticillium dahliae* (Table 69). Based on the test results, both compounds exhibited moderate antifungal activity against the tested fungal species.

**Figure 76:**
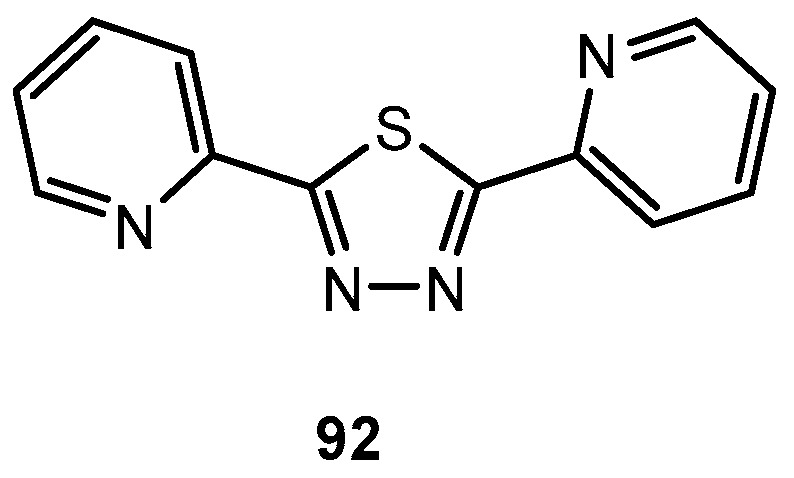
The structure of the obtained ligand of 1,3,4-thiadiazole.

Laachir et al. synthesized a copper(II) coordination polymer based on 2,5-bis(pyridine-2-yl)-1,3,4-thiadiazole (**92**, Figure 76) [115]. The antifungal activity of the [Cu**92**Cl_2_]ₙ complex was evaluated against three strains of the plant pathogenic fungus *Verticillium dahliae* (strains SJ, SH, and SE) and one strain of *Fusarium oxysporum f*. sp. *melonis*. At a concentration of 50 μg/mL, the complex exhibited moderate inhibitory activity against *Verticillium dahliae* strains SH and SE and *Fusarium oxysporum f*. sp. *melonis*.

#### 2.2.2. Bicyclic 1,3,4-Thiadiazole Derivatives

**Figure 77:**
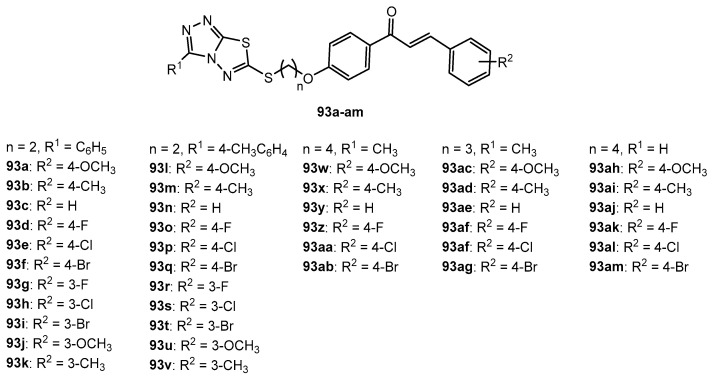
The structure of chalcone derivatives containing 1,2,4-triazolo[3,4-*b*]-1,3,4-thiadiazole.

Zhan et al. synthesized a series of derivatives containing a fused 1,3,4-thiadiazole ring (**93a**–**am**, Figure 77) [116]. The compounds were evaluated for antifungal activity against *Rhizoctonia solani*, *Sclerotinia sclerotiorum*, *Botryosphaeria dothidea*, *Fusarium graminearum*, *Colletotrichum capsici*, *Phytophthora capsici*, and *Phomopsis* sp. at a concentration of 100 μg/mL (Table 70). Compounds **93ae**, bearing a phenyl group at the R^2^ site, and **93af**, with a 4-fluorophenyl group at the same position, exhibited inhibition rates of 64–73% against *Phomopsis* sp., surpassing the activity of the reference fungicide Azoxystrobin. Structure–activity relationship analysis also revealed that the presence of a methyl group at the R^1^ site, together with a tetramethylene linker (n = 4), resulted in enhanced activity against *Colletotrichum capsici* compared to arrangements containing a shorter trimethylene linker (n = 3). In studies involving the *Phytophthora capsici* strain, the highest activity was observed for compounds carrying electron-donating groups (4-CH_3_, 4-OCH_3_) at the R^2^ site.

**Figure 78:**
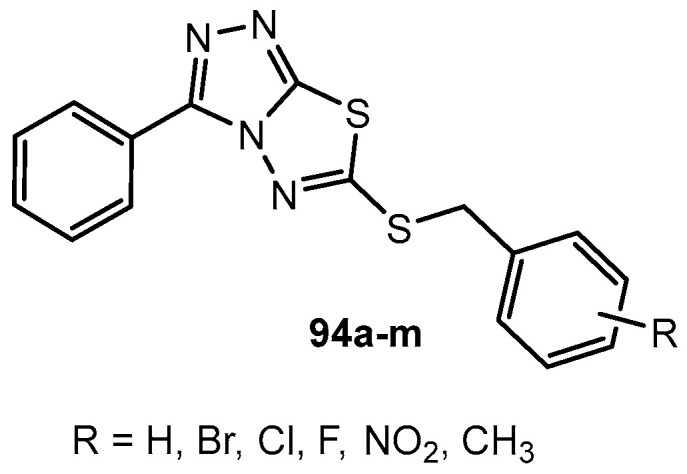
The structure of [1,2,4]-triazolo-[3,4-*b*]-[1,3,4]-thiadiazoles.

Singh et al. synthesized a series of derivatives bearing a 1,3,4-thiadiazole ring fused with a 1,2,4-triazole moiety (**94a**–**m**, Figure 78) [117]. The compounds were screened for antifungal activity against *Fusarium oxysporum* and *Penicillium citrinum*. The results obtained at a concentration of 100 ppm are presented in Table 71. Among the tested derivatives, compound **94c**, containing a (4-chlorobenzyl)thio moiety attached to the fused core, exhibited fungicidal activity comparable to that of the reference fungicides Griseofulvin and Dithane M-45.

Mahdavi’s group synthesized a series of [1,2,4]triazolo[3,4-*b*][1,3,4]thiadiazole derivatives (**60a**–**n**, Figure 49) [88]. All compounds were evaluated for antifungal activity against *Cryptococcus neoformans* (Table 72). Among them, the monochloro-substituted isomers **60f** (R = 3-Cl) and **60g** (R = 4-Cl), as well as the dichloro-substituted derivative **60h** (R = 3,4-diCl), exhibited significant activity, with MIC values of 1, 2, and 0.5 μg/mL, respectively, outperforming or matching the efficacy of the reference drug Fluconazole (MIC = 2 μg/mL).

Wu et al. synthesized a series of quinazolin-4(3*H*)-one derivatives incorporating a 1,2,4-triazolo[3,4-*b*][1,3,4]thiadiazole moiety (**61a**–**ai**, Figure 50) [89]. In addition to antibacterial evaluation, the compounds were tested for antifungal activity against *Phytophthora nicotianae*, *Gibberella zeae*, *Fusarium solani*, *Alternaria tenuissima*, *Colletotrichum gloeosporioides*, *Sclerotinia sclerotiorum*, and *Fusarium oxysporum* (Table 73). Several derivatives exhibited notable inhibitory effects against specific fungal strains. In particular, compounds **61o** (R = 3-CH_3_C_6_H_4_), **61s** (R = 4-NO_2_C_6_H_4_), **61w** (R = 4-OCF_3_C_6_H_4_), **61aa** (R = 3-pyridyl), and **61ab** (R = 2-pyridyl), demonstrated inhibition rates above 50% against *Phytophthora nicotianae*, with compound **61s**, bearing a 4-nitrophenyl substituent, showing the highest activity (67.2%).

As previously mentioned, Kamoutsis et al. synthesized a range of fused 1,3,4-thiadiazole derivatives (**62a**–**s**, Figure 51) [90]. The compounds were evaluated for antifungal activity against six fungal strains: *Aspergillus versicolor*, *Trichoderma viride*, *Aspergillus niger*, *Penicillium verrucosum var. cyclopium*, *Penicillium funiculosum*, and *Aspergillus fumigatus* (Table 74). Several derivatives exhibited antifungal activity up to 80 times greater than Ketoconazole and 3 to 40 times greater than Bifonazole, both used as reference drugs (MIC: 2–40 μg/mL; MFC: 5–67 μg/mL).

**Figure 79:**
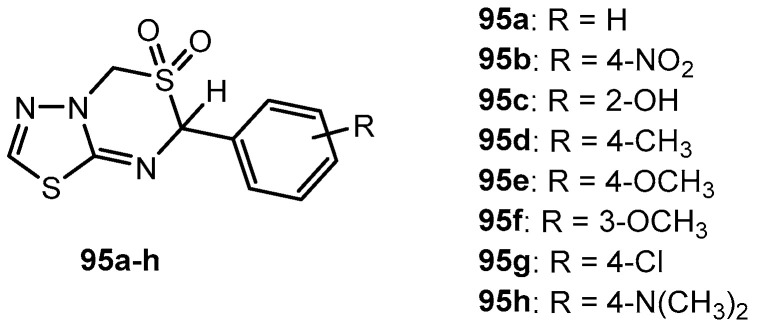
The structure of bicyclic 1,3,4-thiadiazole derivatives.

Borthakur et al. synthesized a series of thiadiazolo-thiadiazine derivatives (**95a**–**h**, Figure 79) [118]. All compounds were screened for antifungal activity against two fungal species, *Rhizoctonia solani* and *Drechslera oryzae*, using Carbendazim as the reference fungicide (Table 75). Among the tested derivatives, only compound **95b**, containing a nitro group at the 4 position, compound **95d**, containing a methyl group at the 4 position, and compound **95g**, containing chlorine at the 4 position, exhibited mild to moderate antifungal activity.

Bhadraiah et al. synthesized a series of bicyclic 1,3,4-thiadiazolo[3,2-α]pyrimidine analogues (**63a**–**i**, Figure 52) [91]. In addition to antibacterial evaluation, the compounds were tested for antifungal activity against *Aspergillus flavus*, *Aspergillus niger*, *Fusarium oxysporum*, and *Fusarium moniliforme*, using Nystatin as the reference drug (Table 76). Among the synthesized derivatives, compound **63c**, containing chlorine at the R^2^ site, and compound **63i**, bearing chlorines at the R^1^ and R^2^ sites, exhibited notable antifungal activity.

#### 2.2.3. Multi-Substituted 1,3,4-Thiadiazole Derivatives

Dai et al. synthesized a series of 5-sulfonyl-1,3,4-thiadiazole-substituted flavonoids (**68a**–**ah**, Figure 55) [94] initially evaluated for antibacterial activity. The compounds were also tested for antifungal activity against *Botrytis cinerea*, *Alternaria solani*, *Rhizoctonia solani*, *Gibberella zeae*, and *Colletotrichum orbiculare*. Inhibition rates at a concentration of 10 μg/mL are presented in Table 77. The results indicated that most of the target compounds exhibited significant antifungal activity. Against *Botrytis cinerea*, six halogen- and alkyl-containing compounds—**68b** (R^1^ = 6-Br, R^2^ = CH_3_, R^3^ = CH_3_), **68d** (R^1^ = 6-Cl, R^2^ = CH_3_, R^3^ = CH_3_), **68h** (R^1^ = 6-Br, R^2^ = CH_2_CH_3_, R^3^ = CH_3_), **68m** (R^1^ = 6-Br, R^2^ = CH_2_CH_2_CH_3_, R^3^ = CH_3_), **68o** (R^1^ = 6-Cl, R^2^ = CH_2_CH_2_CH_3_, R^3^ = CH_3_), and **68af** (R^1^ = 6-Cl, R^2^ = CH_3_, R^3^ = CH_2_CH_3_)—showed high inhibition rates exceeding 80%, with the 6-bromine-containing compound **68b** demonstrating the strongest effect (98%).

As previously mentioned, Gomha et al. synthesized a series of 1,4-dihydropyridine–1,3,4-thiadiazole hybrids (**69a**–**h**, Figure 56) [95]. In addition to antibacterial evaluation, the compounds were assayed in vitro for antifungal activity against *Aspergillus niger* and *Geotrichum candidum* at a concentration of 30 μg/mL (Table 78), using Amphotericin as the reference fungicide. All tested derivatives exhibited high antifungal activity against the selected fungal strains.

Rashdan and coworkers synthesized a series of 1,3,4-thiadiazole derivatives (**70a**–**e**, Figure 57) [96], which, in addition to antibacterial evaluation, were also tested for antifungal activity against *Candida albicans*. The compounds exhibited low activity, with MIC values of 20 μg/mL, in comparison to the reference drug Nystatin (MIC = 5 μg/mL).

Rashdan and coworkers also evaluated another previously reported series of 1,3,4-thiadiazole derivatives (**71a**–**f**, Figure 58) [97] for antifungal activity, alongside their antibacterial assessment. The compounds were screened against the black fungal strain *Rhizopus oryzae* at a concentration of 20 mg/mL (Table 79). The results indicated that compound **71f**, containing a nitro group at the R^1^ site and a phenylamino substituent at the R^2^ site, exhibited significantly higher inhibitory potency against the tested strain.

## 3. Conclusions

Over the last decade, 1,3,4-thiadiazole derivatives have emerged as key scaffolds in medicinal chemistry due to their broad-spectrum biological activities and structural versatility. This review highlights recent advancements (2020–2025) in the development of thiadiazole-based compounds with notable antibacterial and antifungal properties. The antimicrobial activity of these derivatives is often attributed to the presence of a toxophoric N–C–S moiety and their ability to interact with biological targets via hydrogen bonding or metal ion coordination. The mesoionic character and favorable physicochemical properties of the 1,3,4-thiadiazole ring, such as balanced lipophilicity, molecular weight, and hydrogen bonding capacity, contribute to enhanced membrane permeability and bioavailability. These features support their potential as effective enzyme inhibitors and receptor ligands. An important role in the modulation of antimicrobial activity is also played by substituents attached to the thiadiazole ring, either directly or indirectly through an appropriate aromatic or aliphatic linker. Studies have shown that the substituents enhancing biological activity in the analyzed systems include hydroxyl, methoxy, trifluoromethyl, nitro, alkyl groups, and halogens, particularly fluorine and bromine. Biological studies conducted on 10 Gram-negative bacterial strains revealed that 53 novel thiadiazole derivatives exhibited activity exceeding that of the reference drugs or demonstrated a high level of growth inhibition (90–100%). The highest susceptibility was observed for *Xanthomonas oryzae pv. oryzae* (*Xoo*), against which 19 compounds showed antibacterial efficacy above 90%. Significant activity was also noted against *Escherichia coli* (14 compounds) and *Pseudomonas aeruginosa* (7 compounds). Similarly, studies performed on nine Gram-positive bacterial strains demonstrated that 26 derivatives exhibited activity surpassing the reference drugs or achieving a high level of growth inhibition (90–100%). The highest susceptibility was observed for *Enterococcus faecium*, with 17 compounds displaying antibacterial efficacy exceeding 90%. Very high activity was also recorded against *Staphylococcus aureus*, where seven compounds produced excellent results.

In antifungal assays, thiadiazoles were evaluated against 25 fungal species belonging to 15 genera. Among the tested derivatives, 75 novel compounds exhibited growth inhibition surpassing that of the reference drugs or within the 90–100% range. The most susceptible fungi proved to be *Rhizoctonia solani*, against which 18 compounds displayed fungicidal activity above 90%, followed by *Botrytis cinerea* (17 derivatives), *Colletotrichum orbiculare* and *Colletotrichum gloeosporioides* (14 derivatives), and *Aspergillus niger* and *Aspergillus clavatus* (12 derivatives).

In conclusion, 1,3,4-thiadiazole derivatives represent promising candidates for the development of next-generation antimicrobial agents, and ongoing research continues to reinforce their value in both pharmaceutical and agrochemical applications. These results underscore the potential of thiadiazole derivatives as versatile scaffolds for the rational design of novel antimicrobial agents.

## Figures and Tables

**Table 1:** Antibacterial activity of compounds **3a**–**n**.

Entry	Compound	R^2^	R^3^	MIC (μM)			
				*S. epidermidis*	*S. aureus*	*E. faecalis*	*E. faecium*
1	**3a**	H	2-Cl	>100	>100	>100	>100
2	**3b**	4-OH	2-Cl	50	100	>100	>100
3	**3c**	2-OH	2-Cl	6.25	12.5	12.5	12.5
4	**3d**	3-OH	2-Cl	>100	>100	>100	>100
5	**3e**	3-Br-4-OH	2-Cl	>100	>100	>100	>100
6	**3f**	4-OCH_2_COOCH_3_	2-Cl	>100	>100	>100	>100
7	**3g**	4-OCH_2_COOCH_3_	2-Cl	>100	>100	>100	>100
8	**3h**	2-OH-5-OCH_3_	H	>100	>100	>100	>100
9	**3i**	2-OH	4-Cl	25	25	50	50
10	**3j**	2-OH	3-Cl	25	50	25	25
11	**3k**	2-OH-5-OCH_3_	2-Cl	50	50	100	100
12	**3l**	2,4-diOH	H	>100	>100	>100	>100
13	**3m**	3,4-diOH	H	>100	>100	>100	>100
14	**3n**	2,3-diOH	H	50	50	25	25
15	**3o**	2-OH	H	>100	>100	>100	>100
16	**3p**	2-OH	2-Cl	>100	>100	>100	>100
17	**3q**	2-OH	H	>100	>100	>100	>100
18	**3r**	2-OH	H	>100	>100	>100	>100
19	**3s**	2-OH	H	>100	>100	>100	>100
20	**3t**	2-OH	H	>100	>100	>100	>100
21	Daptomycin	-	-	2	2	2	2

**Table 2:** Antibacterial activity of compounds **6a**–**p**.

Entry	Compound	R	Inhibition Rate (%)			
			*R. solanacearum*		*Xoo*	
			200 μg/mL	100 μg/mL	200 μg/mL	100 μg/mL
1	**6a**	H	100	42.45	<10	17.24
2	**6b**	2-F	100	92.00	98.92	42.24
3	**6c**	2-Cl	100	87.04	34.30	<10
4	**6d**	2-Br	82.09	74.37	47.08	13.48
5	**6e**	2-CH_3_	78.63	45.96	32.33	<10
6	**6f**	2-OCH_3_	47.74	31.78	18.70	<10
7	**6g**	2-OCF_3_	80.08	48.26	28.65	<10
8	**6h**	3-F	94.87	93.81	76.17	54.66
9	**6i**	3-CH_3_	95.40	94.00	80.07	14.95
10	**6j**	3-OCH_3_	70.57	67.56	45.34	31.37
11	**6k**	3-OCF_3_	100	100	100	72.63
12	**6l**	4-F	53.19	33.59	28.59	12.09
13	**6m**	4-CH_3_	74.07	71.37	49.37	41.59
14	**6n**	4-OCH_3_	64.10	53.41	48.40	44.61
15	**6o**	4-OCF_3_	43.47	35.11	<10	<10
16	**6p**	4-NH_2_	71.88	60.60	53.00	41.26

**Table 3:** Antibacterial activity of compounds **7a**,**b**.

Entry	Compound	MIC			
		*E. coli*	*S. typhimurium*	*S. aureus*	*E. feacium*
1	**7a** ^a^	0.12	0.12	0.03	0.12
2	**7b** ^a^	0.5	0.25	0.06	0.25
3	Gentamicin ^b^	7.81	7.81	3.91	7.81

^a^ (MIC mg/mL); ^b^ (MIC µg/mL).

**Table 4:** Antibacterial activity of compounds **8a**–**o**.

Entry	Compound	R	MIC (μg/mL)			
			*E. coli*	*P. aeruginosa*	*S. aureus*	*S. pyogenes*
1	**8a**	H	100	12.5	125	125
2	**8b**	2-Br	250	250	100	250
3	**8c**	4-Br	100	62.5	125	125
4	**8d**	2-Cl	50	125	250	100
5	**8e**	4-Cl	100	50	12.5	100
6	**8f**	2-F	125	250	250	250
7	**8g**	3-F	125	250	100	100
8	**8h**	4-F	100	62.5	100	125
9	**8i**	2-OH	100	100	62.5	100
10	**8j**	4-OH	50	12.5	100	100
11	**8k**	4-CH_3_	100	125	250	100
12	**8l**	4-OCH_3_	250	250	62.5	100
13	**8m**	2-NO_2_	100	100	250	250
14	**8n**	3-NO_2_	125	250	125	250
15	**8o**	4-NO_2_	250	12.5	100	62.5
16	Chloramphenicol	-	50	50	50	50
17	Ciprofloxacin	-	25	25	50	50
18	Norfloxacin	-	10	10	10	10

**Table 5:** Antibacterial activity of compounds **11a**–**l**.

Compound	11a	11b	11c	11d	11e	11f	11g	11h	11i	11j	11k	11l	Streptomycin
Inhibition rate (%)	42.3	32.4	40.1	38.2	27.1	31	25.5	18.1	36.5	19.7	14.2	18.4	44

**Table 6:** Antibacterial activity of compounds **12a**–**r**.

Entry	Compound	R^1^	R^2^	Inhibition Rate (%)
1	**12a**	CH_3_	CH_3_	72
2	**12b**	CH_3_	CH_2_CH_3_	72
3	**12c**	CH_3_	CH_2_CH_2_CH_3_	79
4	**12d**	CH_3_	CH_2_C_6_H_5_	78
5	**12e**	CH_3_	CH_2_C_6_H_4_-4-F	63
6	**12f**	CH_3_	CH_2_C_6_H_4_-4-Cl	67
7	**12g**	CH_3_	CH_2_C_6_H_4_-3-Cl	61
8	**12h**	CH_3_	CH_2_C_6_H_4_-2-Cl	52
9	**12i**	CH_3_	CH_2_C_6_H_3_-2,4-diCl	76
10	**12j**	CF_3_	CH_3_	72
11	**12k**	CF_3_	CH_2_CH_3_	62
12	**12l**	CF_3_	CH_2_CH_2_CH_3_	75
13	**12m**	CF_3_	CH_2_C_6_H_5_	62
14	**12n**	CF_3_	CH_2_C_6_H_4_-4-F	68
15	**12o**	CF_3_	CH_2_C_6_H_4_-4-Cl	71
16	**12p**	CF_3_	CH_2_C_6_H_4_-3-Cl	83
17	**12q**	CF_3_	CH_2_C_6_H_4_-2-Cl	67
18	**12r**	CF_3_	CH_2_C_6_H_3_-2,4-diCl	76

**Table 7:** Antibacterial activity of compounds **13a**–**i**.

Entry	Compound	R	Zone of Inhibition (mm)		
			*S. aureus*	*E. coli*	*P. vulgaris*
1	**13a**	H	6	0	6
2	**13b**	2-Cl	14	16	14
3	**13c**	3-Cl	12	16	12
4	**13d**	4-Cl	14	18	14
5	**13e**	3-NO_2_	10	8	8
6	**13f**	4-NO_2_	8	10	12
7	**13g**	3-OCH_3_-4-OH	10	12	10
8	**13h**	4-CH_3_	10	12	12
9	**13i**	2-OH	12	14	14
10	Ampicillin trihydrate	-	16	20	20

**Table 8:** Antibacterial activity of compounds **14a**–**c**.

Entry	Compound	MIC (μg/mL)	
		*B. polymyxa*	*P. aeruginosa*
1	**14a**	2.5	2.5
2	**14b**	>256	>256
3	**14c**	>256	>256
4	Kanamycin	4	4

**Table 9:** Antibacterial activity of compound **15**.

Entry	Compound	Zone of Inhibition (mm)			
		*E. coli*	*P. aeruginosa*	*B. subtilis*	*S. aureus*
1	**15**	11	11.5	11	8

**Table 10:** Antibacterial activity of compounds **16a**–**i**.

Entry	Compound	MIC (μg/mL)								
		*BS*	*CD*	*SA*	*SE*	*SP*	*EC*	*KP*	*PA*	*ST*
1	**16a**	12.5	200	3.125	3.125	12.5	1.56	50	25	1.56
2	**16b**	6.25	6.25	25	100	12.5	50	6.25	1.56	12.5
3	**16c**	1.56	0.78	6.25	50	3.125	200	12.5	50	3.125
4	**16d**	3.125	25	50	25	1.56	12.5	25	100	25
5	**16e**	1.56	50	12.5	1.56	3.125	3.125	3.125	12.5	6.25
6	**16f**	400	400	400	400	400	400	400	400	400
7	**16g**	0.78	3.125	100	12.5	0.78	6.25	400	6.25	50
8	**16h**	200	12.5	0.78	0.78	200	0.78	1.56	0.78	100
9	**16i**	100	1.56	1.56	6.25	50	25	0.78	3.125	0.78
10	Ciprofloxacin	3.125	6.25	3.125	3.125	6.25	1.56	1.56	1.56	1.56
11	Vancomycin	1.56	1.56	1.56	0.78	1.56	-	-	-	-

*BS*: *Bacillus subtilis*; *CD*: *Clostridium difficile*; *SA*: *Staphylococcus aureus*; *SE*: *Staphylococcus epidermidis*; *SP*: *Streptococcus pneumoniae*; *EC*: *Escherichia coli*; *KP*: *Klebsiella pneumonia*; *PA*: *Pseudomonas aeruginosa*; *ST*: *Salmonella typhimurium*.

**Table 11:** Antibacterial activity of compounds **18** and **19** and their complexes.

Bacterial Strains	18 Complex MIC µM/L	19 Complex MIC µM/L	18 MIC µM/L	19 MIC µM/L
*S. aureus* MRSA	2	2	25	12.5
*S. aureus* MSSA	2	2	12.5	12.5
*E. coli*	2	2	25	25
*E. faecium*	n.a	0.2	n.a	12.5
*P. aeruginosa*	n.a	0.2	n.a	25
*S. lentus*	n.a	2	n.a	25

n.a—not active.

**Table 12:** Antibacterial activity of compounds **20a**–**g**.

Entry	Compound	R^1^	R^2^	MBC (µM)				
				*SA*	*EF*	*PA*	*EC*	*KP*
1	**20a**	Br	4-OH	14.30	28.60	14.30	14.30	14.30
2	**20b**	Br	4-NH_2_	14.33	28.67	14.33	43.10	14.33
3	**20c**	OCH_3_	3,5-diCl	14.17	28.34	28.34	28.34	28.34
4	**20d**	OCH_3_	4-NO_2_	14.99	14.99	29.98	29.98	14.99
5	**20e**	OCH_3_	4-Br	13.86	13.86	13.86	13.86	13.86
6	**20f**	Cl	4-NO_2_	14.85	14.85	29.69	14.85	14.85
7	**20g**	Br	4-Cl	6.87	6.87	6.87	13.74	13.74

*SA*: *Staphylococcus aureus*; *EF*: *Enterococcus faecalis*; *PA*: *Pseudomonas aeruginosa*; *EC*: *Escherichia coli*; *KP*: *Klebsiella pneumoniae*.

**Table 13:** Antibacterial activity of compounds **21a**–**i**.

Compound	21a	21b	21c	21d	21e	21f	21g	21h	21i	Streptomycin Sulfate
MIC (mg/mL)	0.0625	0.0313	0.0625	-	0.0625	0.0625	0.0625	-	-	44

**Table 14:** Antibacterial activity of compounds **22a**–**j**.

Entry	Compound	R	MIC [μg/mL]				
			*S. aureus*	*B. subtilis*	*E. coli*	*P. aeruginosa*	*M. tuberculosis*
1	**22a**	H	15.67	16.67	31.00	65.33	Resist
2	**22b**	Cl	1.62	1.33	8.67	8.67	3.12
3	**22c**	Br	2.00	2.00	3.33	5.00	6.25
4	**22d**	F	4.67	7.33	7.67	7.33	3.12
5	**22e**	NO_2_	1.67	1.67	4.67	2.67	Resist
6	**22f**	CH_3_	8.00	16.33	17.33	32.67	6.25
7	**22g**	OCH_3_	8.67	8.33	16.67	16.67	1.60
8	**22h**	NH_2_	5.33	4.00	16.00	8.00	1.60
9	**22i**	OH	7.33	9.00	15.33	16.33	Resist
10	**22j**	C_6_H_5_	1.33	2.00	4.00	9.00	0.8

**Table 15:** Antibacterial activity of compounds **28a**–**e**.

Entry	Compound	Zone of Inhibition (mm)			
		*S. aureus*	*B. subtilis*	*K. pneumonia*	*E. coli*
1	**28a**	10	9	12	8
2	**28b**	14	15	12	13
3	**28c**	10	11	-	8
4	**28d**	-	11	9	12
5	**28e**	8	13	-	14
6	Streptomycin	17	16	15	15

**Table 16:** Antibacterial activity of compounds **29a**–**o**.

Entry	Compound	R^1^	R^2^	Inhibition Rate (%)			
				Xoo		Xac	
				200 μg/mL	100 μg/mL	200 μg/mL	100 μg/mL
1	**29a**	CH_3_	Cl	100	94	90	78
2	**29b**	CH_2_CH_3_	Cl	92	78	86	70
3	**29c**	CH_2_CH_2_CH_3_	Cl	81	70	74	61
4	**29d**	CH_2_C_6_H_5_	Cl	60	44	51	40
5	**29e**	CH_2_C_6_H_4_-4-F	Cl	64	50	57	45
6	**29f**	CH_3_	F	90	75	80	61
7	**29g**	CH_2_CH_3_	F	74	60	68	54
8	**29h**	CH_2_CH_2_CH_3_	F	68	54	60	48
9	**29i**	CH_2_C_6_H_5_	F	51	38	47	31
10	**29j**	CH_2_C_6_H_4_-4-F	F	54	41	51	39
11	**29k**	CH_3_	CH_3_	45	32	40	30
12	**29l**	CH_2_CH_3_	CH_3_	37	28	32	20
13	**29m**	CH_2_CH_2_CH_3_	CH_3_	30	20	25	14
14	**29n**	CH_2_C_6_H_5_	CH_3_	24	16	16	8
15	**29o**	CH_2_C_6_H_4_-4-F	CH_3_	28	18	21	12
16	Bismerthiazol	-	-	70	52	57	35
17	Thiodiazole copper	-	-	63	45	35	15

**Table 17:** Antibacterial activity of compounds **32a**–**k**.

Entry	Compound	MIC (μg/mL)					
		*EC*	*KP*	*PA*	*EF*	*BS*	*SA*
1	**32a**	125	125	250	7.81	15.63	125
2	**32b**	125	125	125	7.81	31.25	125
3	**32c**	125	125	125	31.25	125	125
4	**32d**	62.5	62.5	125	3.9	125	125
5	**32e**	125	62.5	125	7.81	125	125
6	**32f**	<0.97	31.25	125	7.81	125	31.25
7	**32g**	7.81	62.5	62.5	7.81	62.5	31.25
8	**32h**	1.95	15.63	31.25	7.81	31.25	7.81
9	**32i**	<0.97	125	125	3.9	125	250
10	**32j**	3.9	125	125	7.81	125	125
11	**32k**	3.9	125	125	3.9	125	62.5
12	Azithromycin	<0.97	<0.97	<0.97	<0.97	<0.97	<0.97

*EC*: *Escherichia coli*; *KP*: *Klebsiella pneumoniae*; *PA*: *Pseudomonas aeruginosa*; *EF*: *Enterococcus faecalis*; *BS*: *Bacillus subtilis*; *SA*: *Staphylococcus aureus*.

**Table 18:** Antibacterial activity of compounds **33a**–**g**.

Entry	Compound	R	Zone of Inhibition (mm)			
			*S. aureus*	*S. epidermidis*	*P. aeruginosa*	*E. coli*
1	**33a**	H	18	19	11	22
2	**33b**	2-F	20	19	11	18
3	**33c**	2-I	16	15	13	-
4	**33d**	2-Cl	20	19	-	-
5	**33e**	3-Cl	16	15	-	15
6	**33f**	4-Cl	20	18	-	17
7	**33g**	4-NO_2_	24	23	-	15

**Table 19:** Antibacterial activity of compounds **34a**–**e**.

Entry	Compound	R	Zone of Inhibition (mm)			
			*S. aureus*	*S. epidermidis*	*P. aeruginosa*	*E. coli*
1	**34a**	H	14	12	17	15
2	**34b**	2-Br	13	12	14	15
3	**34c**	2-Cl	11	13	20	16
4	**34d**	3-NO_2_	14	0	11	11
5	**34e**	3,5-diNO_2_	21	11	27	18

**Table 20:** Antibacterial activity of compounds **35a**–**j**.

Entry	Compound	R	Zone of Inhibition (mm)			
			*S. aureus*	*S. pyogenes*	*P. aeruginosa*	*E. coli*
1	**35a**	H	21	20	22	20
2	**35b**	N(CH_3_)_2_	23	24	26	22
3	**35c**	(OCH_3_)_2_	20	21	22	19
4	**35d**	Cl	17	17	21	18
5	**35e**	NO_2_	14	15	16	15
6	**35f**	OH	21	23	23	22
7	**35g**	OCH_3_	19	21	20	20
8	**35h**	CH_3_	20	18	19	19
9	**35i**	NH_2_	24	25	26	26
10	**35j**	F	20	19	19	18
11	Ampicillin	-	25	25	26	24

**Table 21:** Antibacterial activity of compound **36** and its complexes.

Entry	Compound	Zone of Inhibition (mm)	
		*E. coli*	*S. aureus*
1	**36**	11	22
2	Cr(**36**)_2_Cl_2_]Cl]	21	26
3	[Co(**36**)_2_Cl_2_]Cl	28	15
4	[Cu(**36**)Cl_2_]	32	27
5	Ciprofloxacin	30	25

**Table 22:** Antibacterial activity of compounds **40a**–**g**.

Entry	Compound	R	Zone of Inhibition in (mm)		
			*E. coli*	*M. tuberculosis*	*P. mirabilis*
1	**40a**	OH	24	18	19
2	**40b**	NO_2_	18	12	17
3	**40c**	Cl	22	16	15
4	**40d**	OCH_3_	17	15	18
5	**40e**	CH_3_	12	10	11
6	**40f**	N(CH_3_)_2_	16	14	12
7	**40g**	OC_2_H_5_	21	19	14
8	Amoxicillin	-	20	17	18

**Table 23:** Antibacterial activity of compound **41** and its complexes.

Entry	Compound	Zone of Inhibition (mm)	
		*E. coli*	*S. aureus*
1	**41**	13	15
2	[Fe(**41**)_2_Cl_2_]Cl	31	29
3	[Ni(**41**)Cl_2_]	20	27
4	[Cu(**41**)Cl_2_]	33	23
5	Ciprofloxacin	30	25

**Table 24:** Antibacterial activity of compounds **42a**–**ac**.

Entry	Compound	MIC (μg/mL)	Entry	Compound	MIC (μg/mL)
1	**42a**	1	16	**42p**	0.25
2	**42b**	1	17	**42q**	0.06
3	**42c**	0.25	18	**42r**	0.06
4	**42d**	0.5	19	**42s**	0.06
5	**42e**	0.125	20	**42t**	0.007
6	**42f**	0.015	21	**42u**	0.06
7	**42g**	0.015	22	**42v**	1
8	**42h**	2	23	**42w**	1
9	**42i**	0.5	24	**42x**	1
10	**42j**	0.25	25	**42y**	8
11	**42k**	0.25	26	**42z**	2
12	**42l**	0.25	27	**42aa**	>64
13	**42m**	0.25	28	**42ab**	>64
14	**42n**	2	29	**42ac**	>64
15	**42o**	0.25	30	Acetazolamide	2

**Table 25:** Antibacterial activity of compounds **43**–**45** and their complexes **46** and **47**.

Entry	Compound	MIC (μg/mL)		
		*S. aureus*	*P. aeruginosa*	*E. coli*
1	**43**	500	-	1000
2	**44**	-	-	-
3	**45**	-	-	-
4	**46**	500	-	1000
5	**47**	500	-	1000

**Table 26:** Antibacterial activity of compounds **49a**–**i**, **50a**–**i**, **51a**–**h**, and **52a**–**h**.

Entry	Compound	EC_50_ (μg/mL)		Entry	Compound	EC_50_ (μg/mL)	
		*Xoo*	*Xoc*			*Xoo*	*Xoc*
1	**49a**	51.02	51.81	18	**50i**	31.78	26.54
2	**49b**	55.9	63.50	19	**51a**	100.39	107.15
3	**49c**	54.78	62.12	20	**51b**	107.8	118.91
4	**49d**	40.6	42.53	21	**51c**	61.66	61.20
5	**49e**	47.6	86.29	22	**51d**	190.41	163.17
6	**49f**	95.37	64.08	23	**51e**	182.31	167.98
7	**49g**	51.73	72.13	24	**51f**	175.29	176.67
8	**49h**	48.63	47.62	25	**51g**	138.08	140.29
9	**494i**	44.45	45.80	26	**51h**	143.93	147.33
10	**50a**	51.34	60.72	27	**52a**	16.03	27.69
11	**50b**	78.94	77.23	28	**52b**	28.47	36.47
12	**50c**	38.74	46.97	29	**52c**	3.14	8.83
13	**50d**	40.52	48.17	30	**52d**	51.83	70.02
14	**50e**	33.73	40.23	31	**52e**	31.13	44.20
15	**50f**	88.08	89.66	32	**52f**	41.31	66.12
16	**50g**	80.52	65.66	33	**52g**	44.66	44.59
17	**50h**	33.25	35.49	34	**52h**	59.56	61.33

**Table 27:** Antibacterial activity of compounds **53a**–**d**.

Entry	Compound	R	Zone of Inhibition (mm)			
			*S. aureus*	*S. pyougenes*	*K. pneumoniae*	*E. coli*
1	**53a**	H	15	6	8	17
2	**53b**	C_7_H_15_	10	10	10	12
3	**53c**	C_3_H_7_	15	8	10	13
4	**53d**	C_6_H_5_	19	0	0	12

**Table 28:** Antibacterial activity of compounds **57a**–**l**.

Entry	Compound	R	Zone of Inhibition (mm)			
			*S. aureus*	*B. subtilis*	*E. coli*	*P. aeruginosa*
1	**57a**	H	14	13	15	14
2	**57b**	4-CH_3_	12	13	12	15
3	**57c**	4-OCH_3_	11	12	11	10
4	**57d**	3-CH_3_	15	14	16	14
5	**57e**	3-OCH_3_	15	16	15	14
6	**57f**	4-C_2_H_5_	12	11	12	14
7	**57g**	3-CH_3_-4-OCH_3_	14	13	14	12
8	**57h**	4-Cl-3-CH_3_	11	11	13	12
9	**57i**	4-Cl	12	12	11	12
10	**57j**	4-Br	10	11	12	11
11	**57k**	4-Cl-2-CH_3_	13	14	15	13
12	**57l**	4-Br-2-CH_3_	15	13	14	12
13	Streptomycin	-	17	18	20	18

**Table 29:** Antibacterial activity of compounds **58a**,**b**.

Entry	Compound	Zone of Inhibition in mm and (MIC) in µg/mL							
		*SA*	*EF*	*BC*	*PA*	*EC*	*KP*	*S*	*PV*
1	**58a**	10 (6.25)	12 (25)	-	-	8 (25)	10 (25)	10 (6.25)	8 (25)
2	**58b**	8 (50)	8 (50)	7 (100)	7 (100)	-	10 (25)	10 (25)	-
3	Ampicillin	25	8	32	15	20	32	18	22

*SA*: *Staphylococcus aureus*; *EF*: *Enterococcus faecalis*; *BC*: *Bacillus cereus*; *PA*: *Pseudomonas aeruginosa*; *EC*: *Escherichia coli*; *KP*: *Klebsiella planticola*; *S*: *Salmonella*; *PV*: *Proteus vulgarus*.

**Table 30:** Antibacterial activity of compounds **61a**–**ai**.

Entry	Compound	Inhibition Rate (%)			
		*Xac*	*Xoc*	*Xoo*	*Psa*
1	**61a**	77.3	27.1	30.3	87.5
2	**61b**	78.3	51.1	39.5	42.0
3	**61c**	69.1	52.7	37.8	41.5
4	**61d**	66.0	42.4	53.1	49.9
5	**61e**	63.9	98.4	87.6	32.1
6	**61f**	74.9	99.3	100	38.6
7	**61g**	78.9	93.0	100	47.7
8	**61h**	72.9	44.0	92.8	34.1
9	**61i**	76.9	23.3	31.7	27.1
10	**61j**	78.8	21.9	34.9	55.0
11	**61k**	69.4	47.2	32.2	24.6
12	**61l**	71.3	49.1	15.7	43.9
13	**61m**	80.9	15.1	26.0	52.3
14	**61n**	83.9	47.1	28.4	27.7
15	**61o**	74.6	58.6	20.9	32.1
16	**61p**	72.8	53.3	96.1	34.9
17	**61q**	79.4	13.8	6.3	57.7
18	**61r**	59.6	40.3	13.7	35.4
19	**61s**	68.5	33.2	21.5	28.2
20	**61t**	69.2	72.8	74.9	28.7
21	**61u**	66.7	18.6	34.0	33.2
22	**61v**	48.9	98.6	90.9	28.4
23	**61w**	53.2	73.1	17.5	51.0
24	**61x**	71.2	60.7	10.5	47.9
25	**61y**	70.7	46.3	100	34.2
26	**61z**	76.9	26.6	18.8	24.2
27	**61aa**	75.2	43.9	13.7	34.3
28	**61ab**	70.1	42.9	17.0	38.1
29	**61ac**	76.3	38.1	6.7	31.1
30	**61ad**	70.4	99.9	97.6	31.0
31	**61ae**	67.6	32.5	25.4	25.0
32	**61af**	21.9	100	98.1	38.4
33	**61ag**	53.1	99.7	95.1	45.4
34	**61ah**	48.7	87.2	82.5	37.5
35	**61ai**	74.9	77.7	93.3	47.0
36	**BMT**	58.8	56.6	60.0	64.5

**Table 31:** Antibacterial activity of compounds **62a**–**s**.

Entry	Compound	MBC (μg/mL)					
		*BC*	*SA*	*LM*	*PA*	*EC*	*ST*
1	**62a**	20	40	20	200	10	20
2	**62b**	20	20	20	40	10	20
3	**62c**	20	40	10	20	10	20
4	**62d**	20	0.20	0.020	0.040	0.020	0.020
5	**62e**	20	36	40	20	10	40
6	**62f**	20	20	36	40	10	10
7	**62g**	20	40	20	20	20	20
8	**62h**	36	40	40	73	20	20
9	**62i**	40	40	20	40	10	20
10	**62j**	20	20	40	60	10	10
11	**62k**	23	40	20	67	10	20
12	**62l**	40	37	20	150	10	10
13	**62m**	20	40	20	200	47	20
14	**62n**	20	60	80	80	13	20
15	**62o**	10	40	20	60	10	20
16	**62p**	20	80	20	80	10	20
17	**62q**	20	40	20	80	10	20
18	**62r**	20	40	20	60	10	20
19	**62s**	10	20	20	40	10	10

*BC*: *Bacillus cereus*; *SA*: *Staphylococcus aureus*; *LM*: *Listeria monocytogenes*; *PA*: *Pseudomonas aeruginosa*; *EC*: *Escherichia coli*; *ST*: *Salmonella typhimurium*.

**Table 32:** Antibacterial activity of compounds **63a**–**i**.

Entry	Compound	R^1^	R^2^	MBC (μg/mL)			
				*B. cereus*	*S. aureus*	*E. coli*	*K. pneumoniae*
1	**63a**	H	H	280	260	250	265
2	**63b**	H	OCH_3_	130	140	145	135
3	**63c**	H	Cl	250	255	260	240
4	**63d**	OCH_3_	H	150	165	210	195
5	**63e**	OCH_3_	OCH_3_	130	145	120	135
6	**63f**	OCH_3_	Cl	150	140	160	210
7	**63g**	Cl	H	280	290	275	270
8	**63h**	Cl	OCH_3_	185	215	200	210
9	**63i**	Cl	Cl	240	260	250	250

**Table 33:** Antibacterial activity of compounds **67a**,**b**.

Entry	Compound	Zone of Inhibition (mm)			
		*B. pumilis*	*S. faecalis*	*E. coli*	*E. cloacae*
1	**67a**	19	21	20	17
2	**67b**	21	16	19	18
3	Penicillin G	25	19	-	-
4	Ciprofloxacin	-	-	30	27

**Table 34:** Antibacterial activity of compounds **68a**–**ah**.

Entry	Compound	R^1^	R^2^	R^3^	Inhibition Rate (%)	
					*Xac*	*Xoo*
1	**68a**	H	CH_3_	CH_3_	0	93.91
2	**68b**	6-Br	CH_3_	CH_3_	48.28	70.30
3	**68c**	6-F	CH_3_	CH_3_	22.99	88.34
4	**68d**	6-Cl	CH_3_	CH_3_	43.03	91.57
5	**68e**	6-CH_3_	CH_3_	CH_3_	43.10	82.61
6	**68f**	7-Br	CH_3_	CH_3_	46.51	80.95
7	**68g**	H	CH_2_CH_3_	CH_3_	27.30	97.01
8	**68h**	6-Br	CH_2_CH_3_	CH_3_	67.96	83.62
9	**68i**	6-F	CH_2_CH_3_	CH_3_	46.98	98.99
10	**68j**	6-Cl	CH_2_CH_3_	CH_3_	59.99	93.42
11	**68k**	6-CH_3_	CH_2_CH_3_	CH_3_	63.43	97.62
12	**68l**	H	CH_2_CH_2_CH_3_	CH_3_	69.68	65.17
13	**68m**	6-Br	CH_2_CH_2_CH_3_	CH_3_	31.39	90.31
14	**68n**	6-F	CH_2_CH_2_CH_3_	CH_3_	29.60	98.14
15	**68o**	6-Cl	CH_2_CH_2_CH_3_	CH_3_	57.04	62.75
16	**68p**	6-CH_3_	CH_2_CH_2_CH_3_	CH_3_	36.71	61.30
17	**68q**	7-Br	CH_2_CH_2_CH_3_	CH_3_	40.52	48.02
18	**68r**	H	CH_3_CHCH_3_	CH_3_	44.11	97.22
19	**68s**	6-Br	CH_3_CHCH_3_	CH_3_	48.99	37.73
20	**68t**	6-F	CH_3_CHCH_3_	CH_3_	18.75	98.06
21	**68u**	6-Cl	CH_3_CHCH_3_	CH_3_	37.57.	45.04
22	**68v**	7-Br	CH_3_CHCH_3_	CH_3_	57.26	9.64
23	**68w**	6-F	allyl	CH_3_	57.40	99.39
24	**68x**	6-CH_3_	allyl	CH_3_	39.94	1.49
25	**68y**	H	Bn	CH_3_	45.19	36.92
26	**68z**	6-Br	Bn	CH_3_	34.34	47.66
27	**68aa**	6-F	Bn	CH_3_	29.02	46.53
28	**68ab**	6-Cl	Bn	CH_3_	66.52	83.21
29	**68ac**	6-CH_3_	Bn	CH_3_	29.09.	38.58
30	**68ad**	7-Br	Bn	CH_3_	42.31	54.08
31	**68ae**	6-F	CH_3_	CH_2_CH_3_	55.82	70.22
32	**68af**	6-Cl	CH_3_	CH_2_CH_3_	87.43	78.29
33	**68ag**	H	*i*-Pr	CH_2_CH_3_	38.43	96.81
34	**68ah**	6-CH_3_	*i*-Pr	CH_2_CH_3_	46.48	8.80
35	Bismerthiazole	-	-	-	35.85	31.6

**Table 35:** Antibacterial activity of compounds **69a**–**h**.

Entry	Compound	Zone of Inhibition (mm)							
		*SA*	*SE*	*BS*	*SP*	*PA*	*EC*	*KP*	*ST*
1	**69a**	19.3	19.4	22.7	-	-	22.3	18.2	20.4
2	**69b**	18.1	18.7	20.1	-	-	19.3	19.2	18.4
3	**69c**	19.4	22.7	18.3	-	-	25.2	21.2	20.6
4	**69d**	19.9	22.0	17.6	-	-	23.1	22.4	23.0
5	**69e**	23.6	22.4	25.5	-	-	26.9	26.3	27.2
6	**69f**	22.4	20.9	23.8	-	-	24.3	22.5	26.3
7	**69g**	20.1	19.2	24.3	-	-	23.7	19.3	21.6
8	**69h**	22.1	19.8	24.3	-	-	24.2	21.3	23.3
9	Ampicillin	23.7	22.4	32.4	24.5	-	-	-	-
10	Gentamicin	-	-	-	-	22.3	25.4	22.6	23.3

*SA*: *Staphylococcus aureus*; *SE*: *Staphylococcus epidermidis*; *BS*: *Bacillus subtilis*; *SP*: *Streptococcus pyogenes*; *PA*: *Pseudomonas aeruginosa*; *EC*: *Escherichia coli*; *KP*: *Klebsiella pneumoniae*; *ST*: *Salmonella typhimurium*.

**Table 36:** Antibacterial activity of compounds **70a**–**e**.

Entry	Compound	MIC (μg/mL)				
		*E. coli*	*P. aeruginosa*	*P. vulgaris*	*B. subtilis*	*S. aureus*
1	**70a**	20	40	20	10	20
2	**70b**	-	-	-	40	80
3	**70c**	40	160	80	40	20
4	**70d**	160	-	-	80	160
5	**70e**	-	-	-	-	-
6	Ciprofloxacin	5	7	1.25	2.5	1.25

**Table 37:** Antibacterial activity of compounds **71a**–**f**.

Entry	Compound	Zone of Inhibition (mm)			
		*K. pneumoniae*	*P. aeruginosa*	*S. aureus*	*B. subtilis*
1	**71a**	19	20	13	15
2	**71b**	13	15	10	12
3	**71c**	16	18	13	15
4	**71d**	15	17	12	14
5	**71e**	20	22	15	17
6	**71f**	22	25	18	20
7	Ciprofloxacin	18	20	15	17

**Table 38:** Antifungal activity of compounds **6a**–**p**.

Entry	Compound	R	Inhibition Rate (%)			
			*S. sclerotiorum*	*R. solani*	*M. oryzae*	*C. gloeosporioides*
1	**6a**	H	32.14	30.65	28.57	28.27
2	**6b**	2-F	90.48	72.32	55.36	52.98
3	**6c**	2-Cl	67.85	40.78	15.70	16.12
4	**6d**	2-Br	33.04	23.21	29.46	26.19
5	**6e**	2-CH_3_	17.81	52.85	19.11	33.22
6	**6f**	2-OCH_3_	50.00	38.69	31.25	25.89
7	**6g**	2-OCF_3_	<10	31.78	34.13	24.34
8	**6h**	3-F	82.14	57.44	38.99	45.83
9	**6i**	3-CH_3_	83.63	52.98	31.85	38.69
10	**6j**	3-OCH_3_	69.05	54.76	30.95	42.56
11	**6k**	3-OCF_3_	59.52	40.48	41.96	43.15
12	**6l**	4-F	32.14	39.88	26.49	35.42
13	**6m**	4-CH_3_	50.00	27.98	22.02	33.04
14	**6n**	4-OCH_3_	55.36	49.40	33.63	33.93
15	**6o**	4-OCF_3_	23.51	33.63	48.51	41.37
16	**6p**	4-NH_2_	80.06	31.55	22.62	31.85
17	Thifluzamide	-	72.92	98.51	25.89	26.49
18	Carbendazim	-	98.21	100	100	96.13

**Table 39:** Antifungal activity of compounds **72a**–**y**.

Entry	Compound	Inhibition Rate (%)							
		*PP*	*CO*	*CA*	*GZ*	*AS*	*RS*	*FO*	*BM*
1	**72a**	43	7	45	46	47	45	3	0
2	**72b**	89	80	70	67	70	74	69	74
3	**72c**	53	10	45	40	33	21	11	18
4	**72d**	42	0	85	83	77	18	6	3
5	**72e**	51	13	60	60	63	68	17	18
6	**72f**	74	50	55	62	57	39	40	41
7	**72g**	68	30	10	30	47	17	23	27
8	**72h**	42	7	70	25	43	13	14	21
9	**72i**	49	3	20	25	20	11	11	9
10	**72j**	74	27	45	49	57	45	26	24
11	**72k**	79	57	30	37	47	27	43	41
12	**72l**	30	0	25	19	17	16	9	0
13	**72m**	47	40	40	25	37	12	31	35
14	**72n**	79	47	45	43	33	16	31	32
15	**72o**	49	13	30	27	33	16	20	21
16	**72p**	70	40	45	57	53	62	34	32
17	**72q**	42	0	50	37	27	16	11	9
18	**72r**	70	53	70	49	47	19	46	41
19	**72s**	77	53	45	32	33	12	43	47
20	**72t**	42	13	35	35	33	12	11	24
21	**72u**	15	0	55	48	23	12	0	12
22	**72v**	42	7	55	40	63	33	9	18
23	**72w**	57	0	55	24	40	10	14	9
24	**72x**	51	17	45	27	27	10	11	24
25	**72y**	79	57	55	35	60	31	46	38
26	Boscalid	89	50	90	18	67	87	40	38

*PP*: *Physalospora piricola*; *CO*: *Colletotrichum orbiculare*; *CA*: *Cercospora arachidicola*; *GZ*: *Gibberella zeae*; *AS*: *Alternaria solani*; *RS*: *Rhizoctonia solani*; *FO*: *Fusarium oxysporum*; *BM*: *Bipolaris maydis*.

**Table 40:** Antifungal activity of compounds **8a**–**o**.

Entry	Compound	R	MIC (μg/mL)		
			*C. albicans*	*A. niger*	*A. clavatus*
1	**8a**	H	500	500	500
2	**8b**	2-Br	1000	>1000	>1000
3	**8c**	4-Br	250	100	500
4	**8d**	2-Cl	1000	1000	1000
5	**8e**	4-Cl	100	>1000	100
6	**8f**	2-F	250	500	500
7	**8g**	3-F	500	1000	>1000
8	**8h**	4-F	1000	1000	1000
9	**8i**	2-OH	250	100	500
10	**8j**	4-OH	1000	100	1000
11	**8k**	4-CH_3_	500	1000	1000
12	**8l**	4-OCH_3_	250	>1000	>1000
13	**8m**	2-NO_2_	1000	500	500
14	**8n**	3-NO_2_	>1000	500	500
15	**8o**	4-NO_2_	1000	100	250
16	Nystatin	-	100	100	100
17	Griseofulvin	-	500	100	100

**Table 41:** Antifungal activity of selected compounds **73a**–**x**.

Entry	Compound	Pathogens	EC_50_ (μg/mL)
1	**73b**	*Botrytis cinerea*	2.7
2	**73g**	*Botrytis cinerea*	2.9
3	**73o**	*Botrytis cinerea*	5.2
4	Azoxystrobin	*Botrytis cinerea*	14.5
5	**73d**	*Tomato Botrytis cinerea*	3.5
6	**73e**	*Tomato Botrytis cinerea*	14.1
7	**73v**	*Tomato Botrytis cinerea*	25.9
8	Azoxystrobin	*Tomato Botrytis cinerea*	26.5
9	**73b**	*Phomopsis* sp.	6.4
10	**73d**	*Phomopsis* sp.	5.3
11	**73o**	*Phomopsis* sp.	7.8
12	Azoxystrobin	*Phomopsis* sp.	10.4

**Table 42:** Antifungal activity of compounds **74a**–**x**.

Entry	Compound	Inhibition Rate (%)		
		*R. solani*	*Phomopsis* sp.	*P. capsici*
1	**74a**	73.2	83.9	63.1
2	**74b**	59.4	82.6	39.5
3	**74c**	76.2	71.3	83.3
4	**74d**	83.7	91.3	73.0
5	**74e**	73.6	71.3	74.7
6	**74f**	78.2	81.3	41.2
7	**74g**	35.6	15.7	21.9
8	**74h**	72.8	60.9	82.8
9	**74i**	61.5	70.0	68.2
10	**74j**	54.0	59.1	50.6
11	**74k**	60.3	57.4	59.7
12	**74l**	67.4	80.0	75.5
13	**74m**	58.6	56.1	85.0
14	**74n**	46.4	60.9	84.1
15	**74o**	59.4	30.4	75.1
16	**74p**	71.6	82.6	77.7
17	**74q**	32.2	29.6	30.5
18	**74r**	53.1	75.2	60.1
19	**74s**	71.6	67.2	84.5
20	**74t**	73.2	39.1	85.0
21	**74u**	67.8	47.8	41.2
22	**74v**	52.7	39.1	71.7
23	**74w**	40.2	35.7	55.8
24	**74x**	73.6	75.7	82.4

**Table 43:** Antifungal activity of compounds **11a**–**l**.

Compound	11a	11b	11c	11d	11e	11f	11g	11h	11i	11j	11k	11l	Terinafine
Inhibition rate (%)	43.4	3.4	25.8	31.9	4.3	7.3	11.1	14.2	34.3	21.2	9.4	0	50.7

**Table 44:** Antifungal activity of compounds **75a**–**ag**.

Entry	Compound	R^1^	R^2^	Inhibition Rate (%)				
				*B. cinerea*	*A. solani*	*R. solani*	*F. graminearum*	*C. orbiculare*
1	**75a**	H	CH_3_	62.1	43.4	64.8	32.3	51.1
2	**75b**	6-Br	CH_3_	100	87.0	92.4	70.0	87.4
3	**75c**	6-F	CH_3_	34.4	43.7	50.4	28.7	43.8
4	**75d**	6-Cl	CH_3_	100	81.7	83.0	44.5	75.1
5	**75e**	6-CH_3_	CH_3_	59.3	41.3	53.7	28.7	48.6
6	**75f**	7-Br	CH_3_	11.0	32.3	75.4	38.3	13.3
7	**75g**	H	CH_2_CH_3_	100	65.6	85.6	48.7	100
8	**75h**	6-Br	CH_2_CH_3_	100	84.9	91.8	71.8	100
9	**75i**	6-F	CH_2_CH_3_	100	100	100	49.4	100
10	**75j**	6-Cl	CH_2_CH_3_	100	100	100	73.9	100
11	**75k**	6-CH_3_	CH_2_CH_3_	59.9	50.4	62.6	41.1	61.2
12	**75l**	H	Pr	64.3	53.9	76.3	36.1	77.4
13	**75m**	6-Br	Pr	100	71.4	91.2	79.0	93.2
14	**75n**	6-F	Pr	84.9	63.6	86.8	76.1	98.9
15	**75o**	6-Cl	Pr	82.7	69.2	100	59.4	95.4
16	**75p**	6-CH_3_	Pr	39.8	36.6	38.9	28.3	48.8
17	**75q**	7-Br	Pr	16.1	23.9	23.9	14.7	16.9
18	**75r**	H	*i*-Pr	49.1	79.1	89.1	40.6	100
19	**75s**	6-Br	*i*-Pr	100	98.5	100	65.6	100
20	**75t**	6-F	*i*-Pr	44.8	84.9	93.5	51.0	100
21	**75u**	6-Cl	*i*-Pr	100	66.2	100	75.6	100
22	**75v**	6-CH_3_	*i*-Pr	51.8	46.3	45.8	40.0	68.6
23	**75w**	7-Br	*i*-Pr	22.0	36.1	62.1	26.1	27.1
24	**75x**	H	allyl	85.3	59.0	93.9	35.0	83.3
25	**75y**	6-Cl	allyl	100	84.0	100	21.4	30.2
26	**75z**	6-CH_3_	allyl	24.9	19.0	57.4	36.2	32.8
27	**75aa**	7-Br	allyl	33.9	41.7	70.7	17.5	20.0
28	**75ab**	H	Bn	13.9	27.0	23.7	17.3	16.7
29	**75ac**	6-Br	Bn	13.6	39.2	73.6	54.6	39.0
30	**75ad**	6-F	Bn	20.2	25.4	31.7	40.1	33.1
31	**75ae**	6-Cl	Bn	13.7	5.3	12.9	14.7	5.1
32	**75af**	6-CH_3_	Bn	10.7	0	24.0	19.6	11.9
33	**75ag**	7-Br	Bn	6.8	0	14.1	9.5	5.1
34	Carbendazim	-	-	97.1	65.3	100	100	84.3
35	Boscalid	-	-	83.7	35.7	81.9	17.5	-

**Table 45:** Antifungal activity of compounds **12a**–**r**.

Entry	Compound	R^1^	R^2^	Inhibition Rate (%)			
				*C. gloeosporioides*	*N. ishiwadae*	*G. persicaria*	*Fusarium* sp.
1	**12a**	CH_3_	CH_3_	62	74	85	65
2	**12b**	CH_3_	CH_2_CH_3_	83	74	81	59
3	**12c**	CH_3_	CH_2_CH_2_CH_3_	87	66	77	56
4	**12d**	CH_3_	CH_2_C_6_H_5_	85	75	79	74
5	**12e**	CH_3_	CH_2_C_6_H_4_-4-F	60	60	65	45
6	**12f**	CH_3_	CH_2_C_6_H_4_-4-Cl	76	62	72	49
7	**12g**	CH_3_	CH_2_C_6_H_4_-3-Cl	70	51	74	35
8	**12h**	CH_3_	CH_2_C_6_H_4_-2-Cl	56	46	64	34
9	**12i**	CH_3_	CH_2_C_6_H_3_-2,4-diCl	63	70	59	59
10	**12j**	CF_3_	CH_3_	64	74	75	57
11	**12k**	CF_3_	CH_2_CH_3_	79	55	65	43
12	**12l**	CF_3_	CH_2_CH_2_CH_3_	66	63	77	51
13	**12m**	CF_3_	CH_2_C_6_H_5_	68	34	60	23
14	**12n**	CF_3_	CH_2_C_6_H_4_-4-F	78	61	74	51
15	**12o**	CF_3_	CH_2_C_6_H_4_-4-Cl	74	71	72	63
16	**12p**	CF_3_	CH_2_C_6_H_4_-3-Cl	90	37	41	54
17	**12q**	CF_3_	CH_2_C_6_H_4_-2-Cl	77	51	74	39
18	**12r**	CF_3_	CH_2_C_6_H_3_-2,4-diCl	73	66	70	49
19	Prochloraz	-	-	87	98	95	91

**Table 46:** Antifungal activity of compounds **76a**–**t**.

Entry	Compound	R	EC_50_ (95% Cl, μmol/L)				
			*R. solani*	*B. cinerea*	*S. lycopersici*	*C. lunata*	*P. aphanidermatum*
1	**76a**	2-F	0.0285	0.8424	0.1432	0.4226	---
2	**76b**	3-F	2.4311	---	0.0374	0.0237	---
3	**76c**	4-F	0.1297	7.7911	1.2300	---	---
4	**76d**	2-Cl	0.1586	1.2954	---	---	---
5	**76e**	3-Cl	0.0466	---	0.0105	0.0051	0.1603
6	**76f**	4-Cl	0.0415	0.1422	1.0919	1.1636	0.7236
7	**76g**	2-Br	---	0.0045	---	---	0.2273
8	**76h**	4-Br	---	---	0.0590	0.0359	0.2717
9	**76i**	2-CH_3_	0.1303	0.1169	1.8938	0.2065	---
10	**76j**	3-CH_3_	0.0107	---	3.6669	0.3369	---
11	**76k**	4-CH_3_	0.0328	0.9588	---	---	0.0334
12	**76l**	2-OCH_3_	1.5904	0.0024	2.9342	0.4719	-
13	**76m**	3-OCH_3_	0.0079	0.9994	0.0993	0.0119	0.0355
14	**76n**	4-OCH_3_	0.0857	---	---	-	0.0180
15	**76o**	3-CF_3_	0.0160	0.1465	0.0481	0.3058	0.0270
16	**76p**	4-CF_3_	0.0028	---	0.0359	1.0790	1.9811
17	**76q**	2,4-diCl	0.0547	0.0347	0.2990	0.2019	-
18	**76r**	2,4-diF	0.0155	0.1419	8.2794	-	0.4482
19	**76s**	3,4-diOCH_3_	0.0189	0.1708	0.2725	0.0318	3.5046
20	**76t**	3,4,5-triOCH_3_	1.8869	1.1614	0.1054	0.0198	1.1971
21	Difenoconazole	-	0.0019	0.0042	0.0124	0.0014	0.0001

**Table 47:** Antifungal activity of compounds **77a**–**j**.

Entry	Compound	Zone of Inhibition (mm)		
		*C. albicans*	*P*. species	*A. Niger*
1	**77a**	-	-	-
2	**77b**	-	9	5
3	**77c**	6	5	6
4	**77d**	-	6	-
5	**77e**	-	8	-
6	**77f**	-	8	-
7	**77g**	7	10	8
8	**77h**	-	5	-
9	**77i**	-	-	-
10	**77j**	-	8	-

**Table 48:** Antifungal activity of compounds **78a**–**d**.

Entry	Compound	MIC (μg/mL)	
		*C. albicans*	*C. parapsilosis*
1	**78a**	128	64
2	**78b**	>128	>128
3	**78c**	>128	16
4	**78d**	128	64

**Table 49:** Antifungal activity of compounds **13a**–**i**.

Entry	Compound	R	Zone of Inhibition (mm)		
			*A. fumigatus*	*C. albicans*	*C. krusei*
1	**13a**	H	6	0	0
2	**13b**	2-Cl	6	16	16
3	**13c**	3-Cl	10	12	14
4	**13d**	4-Cl	12	16	18
5	**13e**	3-NO_2_	10	12	12
6	**13f**	4-NO_2_	0	10	10
7	**13g**	3-OCH_3_-4-OH	0	10	8
8	**13h**	4-CH_3_	6	6	8
9	**13i**	2-OH	8	12	16
10	Fluconazole	-	0	29	20

**Table 50:** Antifungal activity of compounds **14a**–**c**.

Entry	Compound	MIC (μg/mL)				
		*A. niger*	*F. solani*	*P. chrysogenum*	*P. frequentans*	*A. alternata*
1	**14a**	0.125	0.125	0.125	0.125	0.125
2	**14b**	>32	>32	>32	>32	>32
3	**14c**	>32	>32	>32	>32	>32
4	Caspofungin	0.25	0.25	0.25	0.25	0.25

**Table 51:** Antifungal activity of selected compounds **79a**–**ae**.

Entry	Compound	MIC (μg/mL)				
		*G. saubinetii*	*A. solani*	*V. dahliae*	*G. zeae*	*T. cucumeris*
1	**79c**	102.8	91.9	-	-	31.3
2	**79d**	84.2	119.0	-	-	15.3
3	**79i**	86.8	62.5	-	87.3	11.8
4	**79j**	23.3	50.0	88.4	63.0	9.7
5	**79k**	28.0	45.9	89.2	54.9	18.7
6	**79m**	43.3	42.3	58.7	104.0	35.4
7	**79n**	52.2	37.1	73.4	112.7	27.7
8	**79o**	11.4	36.6	69.6	167.5	46.1
9	**79p**	92.7	37.0	-	-	-
10	**79q**	90.1	63.8	-	108.1	25.7
11	**79s**	47.6	56.4	66.2	55.3	51.2
12	**79t**	37.6	42.1	72.1	49.1	20.0
13	**79u**	23.6	41.2	72.9	33.7	24.3
14	**79w**	69.2	70.5	-	-	-
15	**79x**	37.1	49.6	110.6	81.4	34.9
16	**79y**	47.7	76.4	102.9	119.9	32.7
17	**79z**	43.9	35.8	-	93.8	60.6
18	**79aa**	31.4	47.1	95.8	86.3	32.3
19	**79ac**	49.2	53.2	175.8	92.3	116.4
20	**79ad**	91.0	48.2	-	112.9	53.9
21	Triadimefon	14.8	45.3	2.9	16.9	11.0

**Table 52:** Antifungal activity of compounds **80a**–**v**.

Entry	Compound	Inhibition Rate (%)									
		*BC*	*PS*	*CG*	*RS*	*PC*	*SS*	*FcU*	*FG*	*AB*	*FcA*
1	**80a**	55.5	32.2	47.7	54.0	30.0	40.6	20.5	33.2	44.4	44.4
2	**80b**	61.3	40.4	46.0	55.5	38.4	38.2	18.9	36.5	46.0	33.9
3	**80c**	65.1	37.2	47.7	59.3	34.5	63.6	32.3	24.9	51.7	48.8
4	**80d**	36.6	34.1	42.7	38.6	41.8	43.8	35.0	38.5	51.0	40.3
5	**80e**	38.7	60.2	41.8	43.8	36.3	44.6	32.7	31.6	49.8	42.3
6	**80f**	60.1	51.7	32.2	48.5	28.7	10.7	41.7	32.8	35.6	42.3
7	**80g**	74.6	36.8	30.1	55.3	32.1	51.2	29.1	35.7	32.6	39.1
8	**80h**	62.2	53.5	35.6	51.5	31.2	52.6	30.7	28.3	42.9	42.7
9	**80i**	54.4	55.0	36.0	52.2	49.4	53.8	31.1	31.6	39.8	47.6
10	**80j**	56.7	37.2	38.9	47.1	51.9	45.0	30.3	33.2	28.7	41.5
11	**80k**	33.3	27.1	24.3	57.4	35.7	45.7	46.5	33.5	36.8	47.6
12	**80l**	91.7	59.6	36.0	55.5	24.5	54.2	37.8	35.7	39.5	45.6
13	**80m**	83.2	42.8	41.8	54.0	34.2	47.0	46.5	35.7	39.8	52.4
14	**80n**	69.1	73.8	54.0	65.6	16.8	71.5	40.9	20.2	47.9	50.8
15	**80o**	58.8	59.0	36.0	40.1	32.9	57.4	39.4	51.6	34.5	47.6
16	**80p**	50.4	50.2	23.9	40.8	32.5	48.8	39.4	31.2	30.3	51.6
17	**80q**	87.3	66.1	46.4	65.2	22.4	65.3	34.3	28.4	48.3	50.4
18	**80r**	96.2	65.3	45.2	63.4	22.4	58.7	29.5	18.0	47.1	51.2
19	**80s**	60.1	49.4	41.0	45.6	28.3	47.0	41.3	32.0	40.2	46.4
20	**80t**	65.9	42.8	34.3	45.2	49.8	45.0	26.0	26.6	31.4	46.4
21	**80u**	58.4	72.8	45.2	46.3	46.8	66.9	35.4	44.3	42.5	48.4
22	**80v**	52.7	38.0	37.7	52.8	52.7	41.7	28.7	37.7	36.8	44.4
23	Azoxystrobin	80.7	58.1	56.1	75.7	75.5	71.9	48.0	36.6	24.5	60.9

*RS*: *Rhizoctonia solani*; *BC*: *Botrytis cinereal*; *FG*: *Fusarium graminearum*; *CG*: *Colletotrichum gloeosporioides*; *SS*: *Sclerotinia sclerotiorum*; *PC*: *Phytophthora capsica*; *AB*: *Alternaria brassicae*; *FcU*: *Fusarium oxysporum f*. sp. *Cucumerinum*; *FcA*: *Fusarium oxysporum f*. sp. *Capsicum*; *PS*: *Phomopsis* sp.

**Table 53:** Antifungal activity of compounds **16a**–**i**.

Entry	Compound	MIC (μg/mL)				
		*A. niger*	*A. flavus*	*C. albicans*	*S. cerevisiae*	*F. oxysporum*
1	**16a**	200	50	25	12.5	25
2	**16b**	50	25	0.78	6.25	25
3	**16c**	100	6.25	1.56	25	6.25
4	**16d**	12.5	1.56	3.125	1.56	200
5	**16e**	6.25	200	6.25	100	6.25
6	**16f**	400	400	400	400	400
7	**16g**	50	0.78	3.125	3.125	1.56
8	**16h**	3.125	12.5	200	400	1.56
9	**16i**	0.78	3.125	12.5	0.78	0.78
10	Miconazole	1.56	1.56	3.125	3.125	3.125
11	Fluconazole	1.56	0.78	0.78	0.78	0.78

**Table 54:** Antifungal activity of compounds **20a**–**g**.

Entry	Compound	R^1^	R^2^	MIC (µM)	
				*T. harzianum*	*A. niger*
1	**20a**	Br	4-OH	7.15	28.60
2	**20b**	Br	4-NH_2_	28.67	28.67
3	**20c**	OCH_3_	3,5-diCl	14.17	28.34
4	**20d**	OCH_3_	4-NO_2_	14.99	14.99
5	**20e**	OCH_3_	4-Br	6.93	13.86
6	**20f**	Cl	4-NO_2_	3.71	29.69
7	**20g**	Br	4-Cl	6.87	3.43
8	Fluconazole	-	-	5.10	5.10

**Table 55:** Antifungal activity of compounds **24a**–**c**.

Entry	Compound	R	MIC (μg/mL)		
			*C. albicans*	*A. fumigatus*	*P. chrysogenum*
1	**24a**	H	64	128	64
2	**24b**	Br	8	16	8
3	**24c**	CH_3_	16	32	16
4	Fluconazole	-	4	8	8

**Table 56:** Antifungal activity of selected compounds **81a**–**ae**.

Entry	Compound	EC_50_ (100 μg/mL)				
		*G. saubinetii*	*V. dahliae*	*A. solani*	*G. zeae*	*T. cucumeris*
1	**81a**	30.5	48.1	61.7	59.8	32.8
2	**81b**	21.9	45.4	67.5	42.8	22.2
3	**81c**	21.5	41.6	63.4	37.3	39.6
4	Triadimefon	14.8	2.9	45.3	16.9	11.0
5	Tebuconazole	0.4	0.1	1.3	0.4	0.6

**Table 57:** Antifungal activity of compounds **25a**–**l**.

Entry	Compound	MIC (μg/mL)	
		*C. albicans*	*A. niger*
1	**25a**	-	-
2	**25b**	1024	512
3	**25c**	512	>1024
4	**25d**	128	128
5	**25e**	-	-
6	**25f**	-	-
7	**25g**	8	64
8	**25h**	-	-
9	**25i**	-	-
10	**25j**	128	128
11	**25k**	1024	>1024
12	**25l**	512	512
13	Fluconazole	4	256

**Table 58:** Antifungal activity of compounds **82a**–**u**.

Entry	Compound	Inhibition Rate (%)							
		*FO*	*CA*	*PP*	*AS*	*GZ*	*RS*	*BM*	*CO*
1	**82a**	19.1	23.0	33.1	57.6	31.2	12.2	19.5	14.5
2	**82b**	23.6	31.7	48.5	43.3	28.2	12.2	19.5	14.5
3	**82c**	34.1	64.3	94.8	56.2	56.6	19.8	31.3	31.6
4	**82d**	28.2	14.3	48.5	57.6	25.2	38.3	19.5	14.5
5	**82e**	14.5	14.3	25.4	48.1	40.3	14.3	19.5	14.5
6	**82f**	28.2	31.7	33.1	24.3	25.2	12.2	33.8	28.2
7	**82g**	23.6	14.3	17.7	52.9	31.2	31.7	19.5	14.5
8	**82h**	32.7	40.4	33.1	67.1	31.2	47.0	33.8	37.3
9	**82i**	69.1	83.9	79.2	76.7	70.6	86.1	71.9	73.6
10	**82j**	23.6	31.7	17.7	57.6	34.2	62.2	29.0	32.7
11	**82k**	28.2	23.0	17.7	52.9	25.2	53.5	24.3	23.6
12	**82l**	28.2	14.3	17.7	57.6	25.2	31.7	19.5	23.6
13	**82m**	23.6	23.0	17.7	52.9	28.2	31.7	29.0	37.3
14	**82n**	35.9	64.3	33.9	45.9	51.4	17.3	35.5	33.5
15	**82o**	28.2	57.8	48.5	52.9	52.4	53.5	33.8	32.7
16	**82p**	14.5	23.0	17.7	33.8	40.3	31.7	19.5	23.6
17	**82q**	23.6	14.3	17.7	43.3	31.2	42.6	15.5	23.6
18	**82r**	46.4	66.5	48.5	67.1	52.4	70.9	43.3	46.4
19	**82s**	18.2	14.3	33.1	43.3	43.3	62.2	33.8	32.7
20	**82t**	18.2	14.3	33.1	57.6	31.2	25.2	33.8	23.6
21	**82u**	13.6	14.3	17.7	38.6	43.3	42.6	29.0	23.6
23	Chlorothalonil	100	73.3	75.0	73.9	73.1	96.1	90.4	91.3

*FO*: *Fusarium oxysporum f*. sp. *Cucumerinum*; *CA*: *Cercospora arachidicola*; *PP*: *Physalospora piricola*; *AS*: *Alternaria solani*; *GZ*: *Gibberella zeae*; *RS*: *Rhzioeotnia solani*; *BM*: *Bipolaris maydis*; *CO*: *Colleterichum orbicalare*.

**Table 59:** Antifungal activity of compounds **28a**–**e**.

Entry	Compound	Zone of Inhibition (mm)		
		*A. flavus*	*A. niger*	*R. stolonifera*
1	**28a**	8	-	-
2	**28b**	8	7	8
3	**28c**	10	16	12
4	**28d**	8	-	-
5	**28e**	-	-	-
6	Fluconazole	20	19	21

**Table 60:** Antifungal activity of compounds **83a**–**t**.

Entry	Compound	R^1^	R^2^	Inhibition Rate (%)		
				*B. dothidea*	*Phomopsis* sp.	*B. cinerea*
1	**83a**	H	2-CH_3_	41.8	50.6	73.2
2	**83b**	H	2-F	63.0	83.2	78.7
3	**83c**	H	4-F	75.6	89.6	85.1
4	**83d**	H	2-Cl	57.4	74.6	71.1
5	**83e**	H	3-Cl	65.9	79.4	79.2
6	**83f**	H	4-Cl	72.4	84.5	84.9
7	**83g**	H	2-CN	80.0	88.7	86.1
8	**83h**	H	4-CF_3_	82.6	89.2	90.7
9	**83i**	H	3,4-diCl	70.8	84.6	85.4
10	**83j**	CH_3_	2-CH_3_	36.2	42.9	65.3
11	**83k**	CH_3_	4-F	59.0	71.6	74.0
12	**83l**	CH_3_	2-Cl	51.5	64.5	65.7
13	**83m**	CH_3_	3-Cl	57.4	71.9	73.3
14	**83n**	CH_3_	4-Cl	65.4	78.4	80.4
15	**83o**	CH_3_	2-CN	73.7	76.7	78.8
16	**83p**	CH_3_	2-CF_3_	68.4	80.3	81.8
17	**83q**	CH_3_	4-CF_3_	75.7	86.8	88.3
18	**83r**	CH_3_	2,3-diCl	58.2	69.0	66.5
19	**83s**	CH_3_	2,4-diCl	75.6	82.4	83.9
20	**83t**	CH_3_	3,4-diCl	65.7	78.0	80.8
21	Pyrimethanil	-	-	84.4	85.1	82.8

**Table 61:** Antifungal activity of compounds **29a**–**o**.

Entry	Compound	R^1^	R^2^	Inhibition Rate (%)		
				*B. cinerea*	*V. dahliae*	*F. oxysporum*
1	**29a**	CH_3_	Cl	0	0	2
2	**29b**	CH_2_CH_3_	Cl	17	8	5
3	**29c**	CH_2_CH_2_CH_3_	Cl	21	15	12
4	**29d**	CH_2_C_6_H_5_	Cl	0	2	0
5	**29e**	CH_2_C_6_H_4_-4-F	Cl	0	0	0
6	**29f**	CH_3_	F	0	0	0
7	**29g**	CH_2_CH_3_	F	48	24	16
8	**29h**	CH_2_CH_2_CH_3_	F	55	36	26
9	**29i**	CH_2_C_6_H_5_	F	0	0	0
10	**29j**	CH_2_C_6_H_4_-4-F	F	0	0	0
11	**29k**	CH_3_	CH_3_	0	12	0
12	**29l**	CH_2_CH_3_	CH_3_	61	45	32
13	**29m**	CH_2_CH_2_CH_3_	CH_3_	69	54	40
14	**29n**	CH_2_C_6_H_5_	CH_3_	0	0	0
15	**29o**	CH_2_C_6_H_4_-4-F	CH_3_	9	6	0
16	Carbendazim	-	-	57	79	100

**Table 62:** Antifungal activity of compounds **30a**–**t**.

Entry	Compound	Inhibition Rate (%)				
		*G. zeae*	*B. dothidea*	*P. infestans*	*Phompsis* sp.	*T. cucumeris*
1	**30a**	28.8	24.5	23.6	49.2	45.2
2	**30b**	34.5	32.0	28.1	36.3	33.4
3	**30c**	37.6	31.0	25.6	32.5	56.3
4	**30d**	45.4	25.8	24.7	36.2	42.6
5	**30e**	40.1	26.8	25.3	47.5	47.5
6	**30f**	36.2	21.6	56.4	34.2	43.4
7	**30g**	47.0	33.2	56.7	55.6	46.3
8	**30h**	34.2	48.5	56.1	35.2	35.7
9	**30i**	38.6	54.8	59.8	33.5	45.6
10	**30j**	43.0	51.6	57.5	37.3	55.4
11	**30k**	45.4	50.7	57.6	45.1	43.0
12	**30l**	63.4	50.5	73.5	34.5	59.7
13	**30m**	53.8	46.4	73.1	48.1	68.3
14	**30n**	52.3	66.4	77.5	42.6	56.5
15	**30o**	61.0	65.3	75.1	45.2	56.3
16	**30p**	52.2	66.0	80.1	58.1	56.5
17	**30r**	45.2	54.3	79.7	43.2	58.7
18	**30s**	55.4	55.2	78.0	44.5	65.3
19	**30t**	57.2	54.7	79.3	48.2	68.4
20	Dimethomorph	74.3	72.3	78.2	69.3	68.3

**Table 63:** Antifungal activity of compounds **84a**–**ab**.

Entry	Compound	EC_50_ (μg/mL)	Entry	Compound	EC_50_ (μg/mL)
1	**84a**	83.450	16	**84p**	114.00
2	**84b**	70.725	17	**84q**	82.085
3	**84c**	29.924	18	**84r**	89.406
4	**84d**	41.398	19	**84s**	107.633
5	**84e**	45.246	20	**84t**	90.859
6	**84f**	50.608	21	**84u**	52.089
7	**84g**	43.570	22	**84v**	126.441
8	**84h**	84.770	23	**84w**	101.486
9	**84i**	79.635	24	**84x**	66.656
10	**84j**	40.969	25	**84y**	205.857
11	**84k**	55.469	26	**84z**	9.911
12	**84l**	58.441	27	**84aa**	3.785
13	**84m**	50.201	28	**84ab**	6.049
14	**84n**	43.448	29	Boscalid	47.356
15	**84o**	62.553			

**Table 64:** Antifungal activity of compounds **31a**–**e**.

Entry	Compound	R	Zone of Inhibition (mm)	
			*A. niger*	*A. flavus*
1	**31a**	H	6	6
2	**31b**	Cl	6	10
3	**31c**	F	12	6
4	**31d**	NO_2_	20.5	6
5	**31e**	Br	9	6
6	Fluconazole	-	19.5	25

**Table 65:** Antifungal activity of compounds **32a**–**k**.

Entry	Compound	MIC (μg/mL)			
		*C. albicans*	*C. krusei*	*C. glabrata*	*C. parapsilosis*
1	**32a**	1.95	62.5	3.9	62.5
2	**32b**	15.625	62.5	3.9	62.5
3	**32c**	15.625	62.5	3.9	125
4	**32d**	15.625	125	125	125
5	**32e**	31.25	125	7.81	125
6	**32f**	15.625	125	31.25	125
7	**32g**	7.81	62.5	3.9	62.5
8	**32h**	1.95	7.81	1.95	7.81
9	**32i**	7.81	125	125	62.5
10	**32j**	15.625	125	62.5	62.5
11	**32k**	3.9	125	62.5	62.5
12	Voriconazole	3.9	3.9	1.95	3.9
13	Fluconazole	7.81	7.81	3.9	3.9

**Table 66:** Antifungal activity of compounds **35a**–**j**.

Entry	Compound	R	Zone of Inhibition (mm)			
			*A. niger*		*C. albicans*	
			250 μg/mL	25 μg/mL	250 μg/mL	25 μg/mL
1	**35a**	H	16	11	17	13
2	**35b**	N(CH_3_)_2_	20	15	18	12
3	**35c**	(OCH_3_)_2_	15	-	14	10
4	**35d**	Cl	15	-	14	10
5	**35e**	NO_2_	15	10	16	11
6	**35f**	OH	16	12	15	12
7	**35g**	OCH_3_	18	11	17	13
8	**35h**	CH_3_	17	12	14	10
9	**35i**	NH_2_	19	13	17	12
10	**35j**	F	14	10	17	11
11	Miconazole	-	19	14	17	14

**Table 67:** Antifungal activity of compounds **89a**–**w**.

Entry	Compound	Inhibition Rate (%)							
		*FC*	*CA*	*PP*	*AS*	*GZ*	*RS*	*BM*	*CO*
1	**89a**	62.2	54.2	59.3	40.9	23.8	50.6	31.6	21.6
2	**89b**	8.1	20.8	33.3	0	23.8	50.6	31.6	29.7
3	**89c**	29.7	25.0	59.3	22.7	33.3	44.6	28.9	21.6
4	**89d**	45.9	54.2	40.7	36.4	14.3	62.7	57.9	21.6
5	**89e**	21.6	29.2	59.3	9.1	23.8	53.0	34.2	35.1
6	**89f**	54.1	54.2	77.8	63.6	33.3	32.5	23.7	16.2
7	**89g**	51.4	50.0	29.6	18.2	33.3	53.0	31.6	27.0
8	**89h**	43.2	45.8	59.3	45.5	19.0	44.6	34.2	32.4
9	**89i**	37.8	33.3	88.9	27.3	38.1	8.4	13.2	29.7
10	**89j**	24.3	25.0	51.9	4.5	28.6	8.4	18.4	18.9
11	**89k**	29.7	33.3	25.9	22.7	33.3	38.6	18.4	21.6
12	**89l**	21.6	54.2	59.3	0	14.3	14.5	42.1	21.6
13	**89m**	16.2	25.0	22.2	0	52.4	80.7	60.5	62.2
14	**89n**	40.5	41.7	28.5	40.9	38.1	38.6	31.6	40.5
15	**89o**	56.8	54.2	29.6	27.3	19.0	62.7	39.5	45.9
16	**89p**	56.8	54.2	33.3	40.9	47.6	68.7	50.0	35.1
17	**89q**	54.1	62.5	77.8	50.0	23.8	36.1	21.1	10.8
18	**89r**	10.8	16.7	40.7	9.1	47.6	60.2	36.8	29.7
19	**89s**	27.1	35.7	50.1	24.1	22.8	16.8	24.9	46.7
20	**89t**	28.6	51.6	47.3	28.6	27.4	46.2	39.5	44.3
21	**89u**	18.9	45.8	59.3	0	23.8	26.5	21.1	10.8
22	**89v**	51.4	54.2	29.6	50.0	47.6	62.7	52.6	29.7
23	**89w**	43.2	66.7	31.1	45.5	33.3	62.7	34.2	29.7
24	Chlorothalonil	100	73.3	75.0	73.9	73.1	96.1	90.4	91.3

*FC*: *Fusarium oxysporum f*. sp. *Cucumerinum*; *CA*: *Cercospora arachidicola*; *PP*: *Physalospora piricola*; *AS*: *Alternaria solani*; *GZ*: *Gibberella zeae*; *RS*: *Rhizoeotnia solani*; *BM*: *Bipolaris maydis*; *CO*: *Colleterichum orbicalare*.

**Table 68:** Antifungal activity of compounds **90a**–**r**.

Entry	Compound	Inhibition Rate (%)							
		*FC*	*CA*	*PP*	*AS*	*GZ*	*RS*	*BM*	*CO*
1	**90a**	34.1	64.3	64.3	60.6	54.8	19.8	37.7	35.5
2	**90b**	37.8	67.1	75.2	72.3	47.9	19.8	29.1	35.5
3	**90c**	28.5	52.9	86.1	61.3	56.6	17.3	33.4	25.7
4	**90d**	30.4	50.0	66.5	33.1	47.9	53.9	24.9	29.6
5	**90e**	28.5	64.3	55.7	65.0	58.3	14.9	24.9	27.6
6	**90f**	21.1	50.5	68.7	50.9	39.3	17.3	22.8	25.7
7	**90g**	21.1	70.0	64.3	77.3	56.6	17.3	27.0	27.6
8	**90h**	30.5	80.6	55.5	69.7	56.8	19.3	36.5	33.6
9	**90i**	29.8	69.3	80.2	61.3	58.1	22.3	38.4	28.7
10	**90j**	36.1	66.3	66.3	70.3	65.4	21.8	39.7	49.3
11	**90k**	32.2	64.3	75.2	69.0	47.9	14.9	35.5	33.5
12	**90l**	26.7	52.9	51.3	71.0	30.7	19.8	35.5	29.6
13	**90m**	34.1	67.1	64.3	61.3	27.2	17.3	29.1	31.6
14	**90n**	34.1	61.4	57.8	72.8	79.0	16.1	31.3	37.5
15	**90o**	26.7	72.9	53.5	70.9	56.6	17.3	29.1	25.7
16	**90p**	35.9	72.9	36.1	63.8	51.4	17.3	35.5	37.5
17	**90q**	34.1	70.0	86.1	60.6	58.3	17.3	22.8	29.6
18	**90r**	28.5	61.4	75.2	63.8	51.4	14.9	24.9	27.6
19	Chlorothalonil	100	73.3	75.0	73.9	73.1	96.1	90.4	91.3

*FC*: *Fusarium oxysporum f*. sp. *Cucumerinum*; *CA*: *Cercospora arachidicola*; *PP*: *Physalospora piricola*; *AS*: *Alternaria solani*; *GZ*: *Gibberella zeae*; *RS*: *Rhizoeotnia solani*; *BM*: *Bipolaris maydis*; *CO*: *Colleterichum orbicalare*.

**Table 69:** Antifungal activity of compounds **91a**,**b**.

Entry	Compound	(LD_50_^a^/MIC ^b^/MFC ^c^ μg/mL)		
		*A. solani*	*F. melonis*	*V. dahliae*
1	**91a**	28.7/<1.25/<40	80.3/0.625/>40	14.3/<0.625/20
2	**91b**	26.1/<1.25/<40	27.9/<1.25/>40	23.5/<0.625/>20
3	Thiram 80% ^d^	11.9/>0.625/<3000	11.3/>0.625/<3000	14.34/<1.25/<3000

^a^ LD50: The amount of a compound that causes the death of 50% (one half) of test fungi; ^b^ MIC: Minimum inhibitory concentration; ^c^ MFC: Minimum fungicidal concentration; ^d^ positive control.

**Table 70:** Antifungal activity of compounds **93a**–**am**.

Entry	Compound	Inhibition Rate (%)						
		*RS*	*PS*	*FG*	*CC*	*PC*	*BD*	*SS*
1	**93a**	53.7	27.0	16.4	19.3	24.6	14.2	34.1
2	**93b**	6.3	21.8	16.2	24.1	30.6	19.8	33.7
3	**93c**	10.7	27.4	15.6	26.6	35.8	26.3	27.2
4	**93d**	20.0	12.9	9.3	12.9	6.9	12.6	12.2
5	**93e**	11.5	19.8	16.0	21.4	36.2	5.7	39.0
6	**93f**	11.1	12.5	17.7	31.5	30.6	3.2	35.7
7	**93g**	22.9	30.2	31.5	30.2	34.9	5.2	48.3
8	**93h**	27.0	34.7	24.8	25.4	36.6	14.9	41.8
9	**93i**	22.6	29.4	28.6	19.3	37.1	3.6	36.9
10	**93j**	28.5	32.7	38.2	24.6	31.0	4.8	49.5
11	**93k**	26.3	23.0	19.8	25.4	32.0	6.0	35.7
12	**93l**	21.1	12.5	27.3	21.4	27.6	3.6	33.3
13	**93m**	27.0	12.9	5.9	22.2	28.5	2.4	41.6
14	**93n**	21.5	14.1	24.4	17.7	35.4	2.8	36.5
15	**93o**	26.3	13.7	26.9	19.7	34.	3.6	33.3
16	**93p**	18.9	5.2	17.7	19.4	30.6	3.5	31.7
17	**93q**	27.0	6.8	21.0	16.5	25.9	26.7	33.7
18	**93r**	21.9	23.0	47.5	26.6	25.4	3.2	36.5
19	**93s**	19.2	15.7	34.5	25.4	31.0	9.7	40.2
20	**93t**	17.4	18.2	26.5	29.4	31.5	8.1	34.9
21	**93u**	16.3	14.5	32.4	14.1	28.5	15.3	34.9
22	**93v**	15.2	20.2	34.9	13.3	26.7	6.0	34.5
23	**93w**	22.2	20.3	42.4	14.9	31.5	17.0	27.6
24	**93x**	22.6	19.1	31.1	25.0	32.3	22.2	36.1
25	**93y**	25.2	12.0	25.6	12.9	33.2	3.2	37.4
26	**93z**	6.1	10.4	9.3	12.9	6.9	12.5	46.7
27	**93aa**	23.5	24.5	34.5	37.1	35.4	16.2	12.2
28	**93ab**	21.3	21.6	30.7	11.7	39.2	27.9	33.3
29	**93ac**	19.1	27.8	41.2	33.9	53.5	21.4	41.4
30	**93ad**	22.6	49.0	26.9	29.4	55.1	15.8	47.5
31	**93ae**	28.3	73.1	15.5	48.4	25.9	28.7	49.5
32	**93af**	30.4	64.4	27.7	48.4	19.5	17.4	49.9
33	**93ag**	22.2	29.1	28.6	32.3	34.3	10.1	39.0
34	**93ah**	19.9	21.6	27.3	20.2	28.7	3.6	47.9
35	**93ai**	24.3	10.8	27.3	30.5	26.7	3.8	41.8
36	**93aj**	21.3	17.9	23.1	25.7	36.3	3.8	45.9
37	**93ak**	19.5	14.1	28.6	27.8	35.8	16.2	47.9
38	**93al**	23.5	36.1	46.2	27.8	31.9	12.4	50.8
39	**93am**	22.6	15.4	16.5	32.8	19.5	4.2	50.4
40	Azoxystrobin	58.2	55.6	56.6	52.6	64.7	71.9	73.5

*RS*: *Rhizoctonia solani*; *SS*: *Sclerotinia sclerotiorum*; *BD*: *Botryosphaeria dothidea*; *FG*: *Fusarium graminearum*; *CC*: *Colletotrichum capsica*; *PC*: *Phytophthora capsica*; *PS*: *Phomopsis* sp.

**Table 71:** Antifungal activity of compounds **94a**–**m**.

Entry	Compounds	R	Inhibition Rate (%)	
			*F. oxysporum*	*P. citrinum*
1	**94a**	H	42	33
2	**94b**	4-Br	56	44
3	**94c**	4-Cl	90	95
4	**94d**	4-F	51	40
5	**94e**	4-NO_2_	48	38
6	**94f**	3,4-diCl	38	28
7	**94g**	3-Br	50	38
8	**94h**	3-Cl	67	64
9	**94i**	3-F	68	62
10	**94j**	3-CH_3_	52	48
11	**94k**	2-Br	35	26
12	**94l**	2-Cl	59	61
13	**94m**	2-CH_3_	63	42
14	Dithane M-45	-	96	97
15	Griseofulvin		99	98

**Table 72:** Antifungal activity of compounds **60-n**.

Entry	Compound	R	MIC (µg/mL)
1	**60a**	H	>256
2	**60b**	2-F	>256
3	**60c**	3-F	>256
4	**60d**	4-F	>256
5	**60e**	2-Cl	>256
6	**60f**	3-Cl	1
7	**60g**	4-Cl	2
8	**60h**	3,4-diCl	0.5
9	**60i**	2-Br	>256
10	**60j**	3-Br	>256
11	**60k**	4-Br	>256
12	**60l**	4-NO_2_	>256
13	**60m**	2-CH_3_	>256
14	**60n**	3-CH_3_	>256
15	Fluconazole	-	2

**Table 73:** Antifungal activity of compounds **61a**–**ai**.

Entry	Compound	Inhibition Rate (%)						
		*PN*	*GZ*	*VD*	*FS*	*AT*	*SS*	*FO*
1	**61a**	26.3	37.2	18.1	10.9	13.1	7.2	23.0
2	**61b**	33.7	32.6	18.3	0	25.3	20.1	10.0
3	**61c**	33.5	35.7	26.5	8.3	19.6	25.4	14.3
4	**61d**	24.7	20.9	15.8	0	12.0	7.6	16.8
5	**61e**	11.7	45.0	6.0	3.6	15.5	4.8	11.7
6	**61f**	31.8	37.8	19.0	9.6	14.6	17.9	8.9
7	**61g**	34.8	41.5	24.2	7.8	16.4	8.7	30.1
8	**61h**	35.2	17.4	10.4	6.9	19.9	9.8	11.7
9	**61i**	29.6	24.3	13.6	5.1	18.2	16.1	17.4
10	**61j**	23.6	25.7	14.5	8.6	16.5	14.8	28.9
11	**61k**	31.2	23.8	21.4	0	14.2	10.4	0
12	**61l**	36.4	48.3	24.8	12.6	13.6	9.3	33.5
13	**61m**	23.5	35.0	14.8	8.1	13.8	21.5	15.7
14	**61n**	44.0	21.7	8.5	8.8	12.8	23.4	11.5
15	**61o**	62.7	19.6	41.0	12.1	21.2	24.5	17.9
16	**61p**	39.0	43.4	33.6	6.6	15.9	26.8	21.9
17	**61q**	18.8	18.4	11.4	0	9.6	0	11.5
18	**61r**	26.7	16.5	0	10.4	9.8	8.2	9.7
19	**61s**	67.2	15.7	15.5	8.9	10.3	5.0	8.3
20	**61t**	0	12.7	0	9.6	0	7.1	11.3
21	**61u**	19.9	36.1	13.1	0	12.0	12.6	0
22	**61v**	47.2	4.4	5.8	6.4	8.3	5.3	13.1
23	**61w**	52.8	47.0	40.6	11.2	20.5	28.4	35.8
24	**61x**	6.1	9.4	6.8	8.6	11.9	0	0
25	**61y**	41.6	19.6	36.2	21.7	33.6	50.4	17.6
26	**61z**	41.1	16.5	0	7.8	10.5	0	10.7
27	**61aa**	51.2	22.2	4.8	8.8	18.1	38.6	20.7
28	**61ab**	50.1	23.9	1.8	10.2	10.1	0	8.3
29	**61ac**	29.3	25.0	8.5	12.3	13.4	6.2	13.3
30	**61ad**	48.8	19.6	7.5	8.3	14.0	7.9	10.0
31	**61ae**	34.4	12.9	0	6.1	8.5	0	11.4
32	**61af**	11.6	20.2	8.5	7.8	5.9	8.3	8.6
33	**61ag**	0	12.8	4.4	0	0	0	12.3
34	**61ah**	0	11.9	5.6	7.7	0	4.9	12.1
35	**61ai**	0	12.4	3.8	7.4	0	5.2	15.5
36	Chlorothalonil	84.5	75.1	67.1	64.7	73.5	100	67.5
37	Carbendazim	81.1	100	73.5	100	100	100	100

*PN*: *Phytophthora nicotianae*; *GZ*: *Gibberella zeae*; *FS*: *Fusarium solani*; *AT*: *Alternaria tenuissima*; *VD*: *Colletotrichum gloeosporioides*; *SS*: *Sclerotinia sclerotiorum*; *FO*: *Fusarium oxysporum*.

**Table 74:** Antifungal activity of compounds **62a**–**s**.

Entry	Compound	MIC/MFC (μg/mL)					
		*AF*	*AV*	*AN*	*TV*	*PF*	*PC*
1	**62a**	5/10	2/5	10/20	2/5	20/40	10/20
2	**62b**	10/20	5/10	10/20	2/5	10/20	10/20
3	**62c**	20/40	10/20	15/20	2/5	20/36	20/36
4	**62d**	2/5	2/5	5/1	5/1	10/20	10/20
5	**62e**	20/36	10/20	15/20	8/10	33/40	20/40
6	**62f**	5/10	5/10	5/10	2/5	10/20	10/20
7	**62g**	10/20	10/20	5/10	5/10	5/10	20/36
8	**62h**	10/20	10/20	15/20	5/10	10/20	15/20
9	**62i**	5/10	5/10	10/20	5/10	36/80	30/40
10	**62j**	32/40	20/40	10/20	5/10	10/20	20/40
11	**62k**	20/40	5/10	20/32	2/5	2/5	2/5
12	**62l**	5/20	10/20	15/20	5/10	40/67	20/40
13	**62m**	40/67	20/36	20/40	2/5	40/80	32/40
14	**62n**	10/36	10/20	10/20	5/10	30/40	32/40
15	**62o**	15/20	10/20	20/37	5/10	30/40	30/36
16	**62p**	20/40	10/20	5/1	5/8	10/20	20/40
17	**62q**	20/40	20/40	20/32	5/10	30/40	30/40
18	**62r**	20/32	10/20	10/20	8/10	20/40	30/36
19	**62s**	20/40	20/36	10/20	8/10	20/40	20/40
20	Ketoconazole	20/500	200/500	200/500	1000/1500	200/500	200/300
21	Bifonazole	150/200	100/200	150/200	150/200	200/250	100/200

*AV*: *Aspergillus versicolor*; *TV*: *Trichoderma viride*; *AN*: *Aspergillus niger*; *PC*: *Penicillium verrucosum var. cyclopium*; *PF*: *Penicillium funiculosum*; *AF*: *Aspergillus fumigatus*.

**Table 75:** Antifungal activity of compounds **95a**–**h**.

Entry	Compound	Inhibition Rate (%)			
		*R. solani*		*D. orazae*	
		Concentration 50 (ppm)	Concentration 100 (ppm)	Concentration 50 (ppm)	Concentration 100 (ppm)
1	**95a**	33.25	50.15	36.12	52.19
2	**95b**	46.21	74.80	42.14	76.20
3	**95c**	31.46	50.12	42.09	57.20
4	**95d**	35.34	59.84	30.86	68.76
5	**95e**	40.74	59.35	36.75	49.45
6	**95f**	46.75	52.76	46.54	61.95
7	**95g**	47.37	75.38	38.45	55.20
8	**95h**	38.56	62.24	42.24	60.24
9	carbendazim	96.67	98.56	95.45	98.26

**Table 76:** Antifungal activity of compounds **63a**–**i**.

Entry	Compound	R^1^	R^2^	MFC (μg/mL)			
				*A. flavus*	*A. niger*	*F. oxysporum*	*F. monaliforme*
1	**63a**	H	H	250	265	270	280
2	**63b**	H	OCH_3_	290	285	280	275
3	**63c**	H	Cl	135	120	125	120
4	**63d**	OCH_3_	H	250	220	270	230
5	**63e**	OCH_3_	OCH_3_	135	155	150	165
6	**63f**	OCH_3_	Cl	285	275	280	260
7	**63g**	Cl	H	135	155	140	165
8	**63h**	Cl	OCH_3_	215	220	210	215
9	**63i**	Cl	Cl	120	125	130	130
10	Nystatin	-	-	100	100	100	100

**Table 77:** Antifungal activity of compounds **68a**–**ah**.

Entry	Compound	R^1^	R^2^	R^3^	Inhibition Rate (%)				
					*B. cinerea*	*A. solani*	*R. solani*	*G. zeae*	*C. orbiculare*
1	**68a**	H	CH_3_	CH_3_	69.36	47.71	82.53	54.59	46.88
2	**68b**	6-Br	CH_3_	CH_3_	98.57	100.00	100.00	77.32	92.90
3	**68c**	6-F	CH_3_	CH_3_	69.84	70.00	91.67	71.46	86.53
4	**68d**	6-Cl	CH_3_	CH_3_	89.07	100.00	100.00	81.41	94.67
5	**68e**	6-CH_3_	CH_3_	CH_3_	29.82	48.02	79.13	51.13	65.43
6	**68f**	7-Br	CH_3_	CH_3_	38.37	89.17	100.00	33.37	59.62
7	**68g**	H	CH_2_CH_3_	CH_3_	43.00	59.94	95.57	54.08	92.21
8	**68h**	6-Br	CH_2_CH_3_	CH_3_	84.56	100.00	100.00	81.60	100.00
9	**68i**	6-F	CH_2_CH_3_	CH_3_	42.52	74.01	90.08	67.02	100.00
10	**68j**	6-Cl	CH_2_CH_3_	CH_3_	78.29	100.00	100.00	68.22	100.00
11	**68k**	6-CH_3_	CH_2_CH_3_	CH_3_	31.66	62.44	84.33	46.91	90.87
12	**68l**	H	CH_2_CH_2_CH_3_	CH_3_	60.38	56.88	87.48	38.13	78.14
13	**68m**	6-Br	CH_2_CH_2_CH_3_	CH_3_	80.82	95.72	100.00	51.11	84.84
14	**68n**	6-F	CH_2_CH_2_CH_3_	CH_3_	42.51	90.70	100.00	39.56	97.73
15	**68o**	6-Cl	CH_2_CH_2_CH_3_	CH_3_	81.33	92.68	100.00	50.52	92.56
16	**68p**	6-CH_3_	CH_2_CH_2_CH_3_	CH_3_	32.72	25.73	47.28	20.89	32.22
17	**68q**	7-Br	CH_2_CH_2_CH_3_	CH_3_	32.51	35.03	60.62	17.83	33.09
18	**68r**	H	CH_3_CHCH_3_	CH_3_	33.55	65.77	87.77	47.81	67.93
19	**68s**	6-Br	CH_3_CHCH_3_	CH_3_	72.86	100.00	100.00	50.03	100.00
20	**68t**	6-F	CH_3_CHCH_3_	CH_3_	5.44	94.47	89.17	50.33	79.39
21	**68u**	6-Cl	CH_3_CHCH_3_	CH_3_	72.31	100.00	100.00	55.77	97.06
22	**68v**	7-Br	CH_3_CHCH_3_	CH_3_	22.18	64.92	100.00	70.02	21.12
23	**68w**	6-F	allyl	CH_3_	59.09	39.18	77.34	38.37	91.38
24	**68x**	6-CH_3_	allyl	CH_3_	35.77	17.69	61.59	14.74	37.90
25	**68y**	H	Bn	CH_3_	14.52	26.04	47.78	21.93	11.03
26	**68z**	6-Br	Bn	CH_3_	5.50	39.18	60.28	22.48	13.01
27	**68aa**	6-F	Bn	CH_3_	19.13	18.54	62.60	22.51	20.27
28	**68ab**	6-Cl	Bn	CH_3_	13.61	18.78	1.54	5.89	0.80
29	**68ac**	6-CH_3_	Bn	CH_3_	14.33	0.00	47.46	7.54	6.88
30	**68ad**	7-Br	Bn	CH_3_	11.82	0.00	16.52	3.93	1.38
31	**68ae**	6-F	CH_3_	CH_2_CH_3_	16.04	71.83	100.00	50.47	87.39
32	**68af**	6-Cl	CH_3_	CH_2_CH_3_	85.61	100.00	100.00	66.48	94.80
33	**68ag**	H	*i*-Pr	CH_2_CH_3_	27.38	94.17	94.08	10.62	89.66
34	**68ah**	6-CH_3_	*i*-Pr	CH_2_CH_3_	67.48	83.44	69.72	31.83	82.86

**Table 78:** Antifungal activity of compounds **69a**–**h**.

Entry	Compound	Zone of Inhibition (mm)	
		*A. niger*	*G. candidum*
1	**69a**	21.1	19.8
2	**69b**	22.3	23.4
3	**69c**	18.5	23.2
4	**69d**	19.9	24.1
5	**69e**	25.3	24.2
6	**69f**	24.1	23.6
7	**69g**	23.3	22.3
8	**69h**	25.5	23.2
9	Amphotericin B	23.3	25.2

**Table 79:** Antifungal activity of compounds **71a**–**f**.

Compound	71a	71b	71c	71d	71e	71f	Amphotericin
Zone of inhibition (mm)	9.7	10	12	11	14	17	21

## Data Availability

Not applicable.
